# A disulfide-based linker for thiol–norbornene conjugation: formation and cleavage of hydrogels by the use of light†

**DOI:** 10.1039/d1py00914a

**Published:** 2022-01-10

**Authors:** Markus Lunzer, Boris Maryasin, Tommaso Zandrini, Stefan Baudis, Aleksandr Ovsianikov, Robert Liska

**Affiliations:** Institute of Applied Synthetic Chemistry, Technische Universität Wien Getreidemarkt 9/E163 1060 Vienna Austria robert.liska@tuwien.ac.at; Institute of Materials Science and Technology, Technische Universität Wien Getreidemarkt 9/E308 1060 Vienna Austria; Institute of Organic Chemistry, University of Vienna Währinger Strasse 38 1090 Vienna Austria; Institute of Theoretical Chemistry, University of Vienna Währinger Strasse 17 1090 Vienna Austria

## Abstract

Photolabile groups are the key components of photo-responsive polymers, dynamically tunable materials with multiple applications in materials and life sciences. They usually consist of a chromophore and a labile bond and are inherently light sensitive. An exception are disulfides, simple reversible linkages, which become photocleavable upon addition of a photoinitiator. Despite their practical features, disulfides are rarely utilized due to their impractical formation. Here, we report a disulfide-based linker series bearing norbornene terminals for facile crosslinking of thiol-functionalized macromers *via* light-triggered thiol–ene conjugation (TEC). Besides finding a highly reactive lead compound, we also identify an unexpected TEC-retardation, strongly dependent on the molecular linker structure and affecting hydrogel stability. Finally, we present a useful method for localized disulfide cleavage by two-photon irradiation permitting micropatterning of disulfide-crosslinked networks.

## Abstract 2

A photolinker for formation of disulfide-crosslinked hydrogels *via* light-triggered thiol–ene conjugation has been developed. Hydrogels can be patterned *via* two-photon cleavage of disulfide-linkages in the presence of a two-photon active compound.

## Graphical abstract

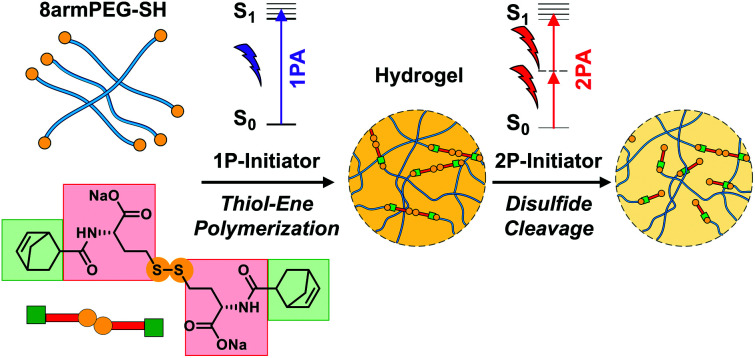

## Introduction

Photo-responsive polymers are highly useful “smart” materials, as their properties can be locally tuned by irradiation with light. Applications include tissue engineering and regenerative medicine,^1^ advanced cell culture^2^ and drug delivery^3^ as well as technical utilizations in self-healing materials,^4^ recycling,^5^ thin films,^6^ and as potential key components for sensors or hydrogel machines.^7^ Hydrogels are especially often modified with photosensitive groups, due to the high transparency of water for light in the visible to near-infra red (VIS-NIR) region.^8–10^ Longer wavelengths allow for deeper penetration and are generally less harmful for living tissue. Light-induced mechanisms include stiffness modifications such as secondary photo-crosslinking,^11,12^ reversible softening^13–16^ or complete degradation^17–20^ of the hydrogel backbone, as well as reversible patterning of biomolecules and proteins^21–24^ or activation of biological tethers by uncaging.^25^ By now, a considerable tool box of light-controllable chemical reactions has been developed.^3,8,26–28^ One of the most commonly used and studied photocleavable triggers is the *o*-nitrobenzyl (*o*NB) group.^19,29–31^ Alternatives are coumarin-derivatives,^32,33^*o*-nitrobiphenylpropyl-derivatives^25,34^ or ruthenium-complexes.^35,36^ All of these phototriggers consist of chromophores as light harvesting units and labile bonds, which can be photocleaved by irradiation with UV-VIS or multiphoton excitation using NIR-light. Due to the inherent light sensitivity of photocleavable groups, handling under light protection is required during all experimental steps including synthesis, analysis and application. Furthermore, the preparation of such chromophores can be quite laborious and usually involves multistep synthetic modifications with often moderate yields.

An interesting but rarely utilized alternative are disulfide linkages. These molecularly quite simple building blocks can be reversibly formed and cleaved by various triggers, but are not photosensitive at ambient light conditions.^37^ Generally, cleavage and remodeling of disulfide-networks relies on the thiol-disulfide metathesis reaction, in which a sulfur-species attacks a disulfide linkage by forming a new disulfide bond and releasing another sulfur-species. The metathesis can be induced chemically *via* a nucleophilic attack of a thiol anion^38^ or photochemically in a radical-mediated reaction.^39^ Due to their reversible character, disulfide networks are dynamic and exhibit self-healing properties.^39–42^ For the mild chemical reduction of disulfides, usually an excess of a small molecule thiol or dithiol reagent is used.^43–45^ Other common reducing agents for disulfides are TCEP^46^ and NaBH_4_.^47^

Photoscission of disulfides in polymers has been directly induced by irradiation with high intensity UV light, resulting in rearrangement and self-healing of materials.^42,48^ Apart from such harsh conditions, disulfide networks are stable at ambient light. Yet, in presence of a photoinitiator, disulfide linkages in hydrogels can be cleaved using UV-VIS light at low intensities through a radical-mediated fragmentation reaction.^39^ Radicals, formed from the scission of the photoinitiator, attack and cleave disulfides, liberating thiyl radicals, which again undergo fragmentation and exchange reactions. Depending on the ratio of radicals to disulfides either photodeformation, photowelding, or photodegradation can be induced. This approach is quite practical, as the weak bond is already present in the network, while the photosensitive trigger is added when required.

Disulfide-based networks are usually directly formed from thiol-terminated precursors by oxidative coupling of thiols to disulfides mediated by oxygen,^49^ hydrogen peroxide,^39,50^ or enzymatically.^51^ However, in oxidative coupling the gelation rate depends on the concentrations of thiol and redox partner, making handling challenging and procedures have to be optimized for each formulation to get controllable results. On the contrary, horseradish peroxidase-mediated crosslinking proceeds slowly.^51^ Hence, it is advantageous to introduce disulfide linkages *via* simple linkers into networks. Disulfide-containing linkers have been studied bearing acrylate or acrylamide terminals, and were either incorporated into polymer networks *via* thiol-Michael addition with thiol-terminated macromers^45^ or by free radical copolymerization with acrylates.^52–54^ However, formulations based on Michael-acceptors and thiols have to be handled very skilled and quickly, as gelation sets in fast upon combining of the reactive components,^18^ which can be especially challenging when it comes to delicate procedures such as cell encapsulation or molding of complex geometries. Besides, acrylates are cytotoxic.^55^

For the ease of handling, an externally triggered gelation-reaction such as photo-induced step growth polymerization would be highly beneficial. As disulfides can be cleaved by increased concentrations of radicals,^39^ a highly efficient coupling reaction is required. The thiol–norbornene addition is exceptionally reactive, but yet well tolerated in cell encapsulation.^56–58^ Hence, to facilitate the fast and mild formation of disulfide-based hydrogels and the general application of this useful linkage, we developed a water-soluble disulfide-containing crosslinker for the light-induced formation of such networks from thiol-terminated precursors by thiol–ene coupling (TEC) reaction. To keep the approach simple and convenient, we aimed at designing the linker to be synthesized from cheap and readily available starting materials.

## Results and discussion

### Design and synthesis of disulfide crosslinkers

**Scheme 1:**
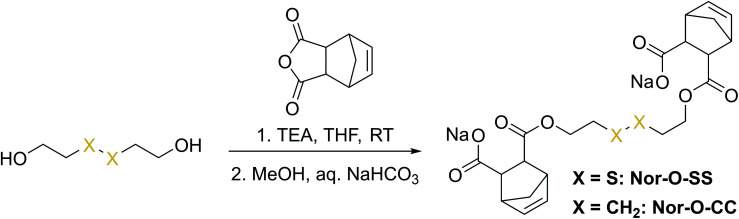
Synthesis of Nor-O-SS and the analog non-cleavable reference compound Nor-O-CC.

The first synthetic approach to such a disulfide-containing thiol–ene-crosslinker was based on the esterification of the readily available chemicals 2-hydroxyethyl disulfide and carbic anhydride, leading to the simple symmetric target molecule Nor-O-SS (Scheme 1), bearing carboxylate groups to facilitate water-solubility. A non-cleavable aliphatic reference linker Nor-O-CC was synthesized as well. However, Nor-O-SS appeared to be self-immolating and thus could not be purified from its decay products (Fig. S20–S22†). The synthesis of the corresponding amide from cystamine seemed unpromising as well.^59^

**Scheme 2:**
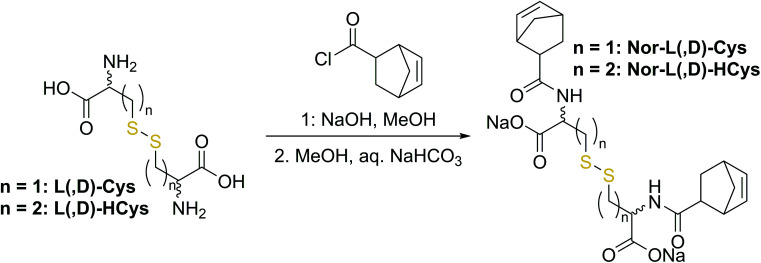
Synthesis of cystine- and homocysteine-based linkers.

In a second approach, the photo-crosslinker was synthesised from l-cystine, the readily available dimer of the amino acid l-cysteine, by coupling with 5-norbornene-carboxylic acid chloride in a Schotten–Baumann-type reaction (Scheme 2).^52^ However, in using cystine as core unit the complexity of the stereo-chemistry increases, since two more stereocenters are integrated into the molecule. For practical and economic reasons, the acid chloride was synthesized from 5-norbornene-2-carboxylic acid in its most common commercial form, a racemic mixture of *exo*- and *endo*-isomers with predominantly *endo*-molecules. Thus, the linker **Nor-****l****-Cys** (Scheme 2, Fig. S23†) was obtained as a mixture of diastereomers, which was used as received without applying complex purification protocols.

Next, a preliminary ^1^H-NMR conversion test with 2-mercaptoethanol was performed to test the reactivity of **Nor-****l****-Cys** (Fig. S22b). Whereas the double bonds of Nor-O-SS were readily consumed within 5 min of irradiation, full conversion of **Nor-****l****-Cys** was surprisingly retarded. Even with a SH : ene ration of 2 : 1, full conversion of **Nor-****l****-Cys** could not be observed within 10 min.

**Fig. 1:**
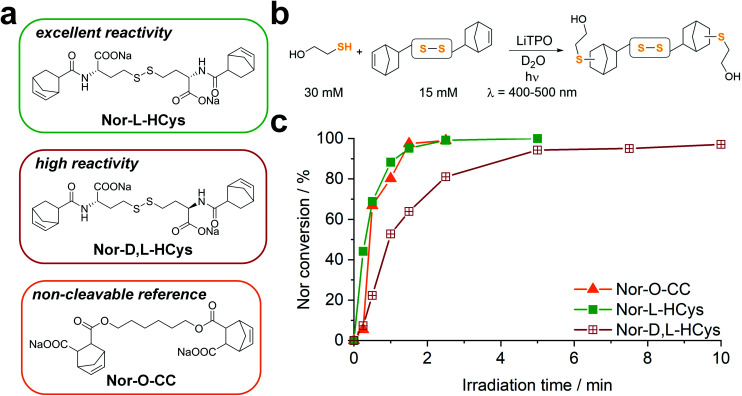
(a) The water-soluble disulfide linkers **Nor-****l****-HCys** and **Nor-****d**,**l****-HCys** for thiol–norbornene conjugation (TEC) were synthesized from l- and d,l-homocysteine. The aliphatic linker Nor-O-CC served as non-cleavable reference. (b) The linker reactivity was investigated in an ^1^H-NMR study. Linkers (15 mM) were reacted with 2-mercaptoethanol (30 mM) in D_2_O using LiTPO as photoinitiator (0.6 mM, *λ* = 400–500 nm, 20 mW cm^−2^). (c) Both Nor-O-CC and **Nor-****l****-HCys** reached full conversion within 2 min of reaction time. In contrast, **Nor-****d**,**l****-HCys** reacted slower and reached high (96%) but not complete ene-conversion after 10 min.

The retarded reactivity of **Nor-****l****-Cys** is probably based on steric interactions, which could be either caused by the molecular configuration of norbornene or the core subunit. Hence, reasonable molecular modification strategies to improve the reactivity towards TEC include both the (I) norbornene group as well as the (II) disulfide-based core unit.

(I) **Nor-****l****-Cys** was synthesized from a mixture of *exo*- and *endo*-5-norbornene-carboxylic acid. Since the ene in *exo*-5-norbornenecarboxylic acid faces away from the carboxylic group, the *exo*-derivative might be sterically less hindered than the corresponding *endo*-based analogue. Thus, to test the influence of the stereoisomerism of the norbornene group, the *exo*-only linker ***exo*-Nor-****l****-Cys** (Fig. S23†) was synthesized from l-cystine and *exo*-5-norbornene carboxylic acid.

(II) In contrast to 2-hydroxyethyl disulfide, cystine is a chiral compound containing two stereocenters. Due to the close proximity of the functional groups, their relative orientation might affect the reactivity of the norbornenes by intramolecular interactions. Additionally, disulfide dihedral angles are usually in the range of 90°,^60^ adding up to the sterical effects based on the configuration of functional groups. The core unit can be simply modified by changing the enantiopure l-cystine to the racemic d,l-cystine giving the linker **Nor-****d**,**l****-Cys** (Fig. S23,†Scheme 2). A further strategy to reduce steric hindrance between the two branches of the molecule is elongation of the core by exchanging cystine for homocystine, containing an additional methylene group per subunit (**Nor-****l****-HCys**, **Nor-****d**,**l****-HCys**, Fig. 1, Scheme 2). Finally, as the water solubility of the molecule is facilitated by carboxylate anions, also hydratization effects could impact the reactivity of the ene. Hence, the reactivity of the linker in aprotic medium was tested as well. Hence, the dimethylated linker **Nor-****l****-CysMe** was synthesised (Fig. S23c†).

### Reactivity estimation by ^1^H-NMR

To examine the reactivity of the linkers towards TEC and evaluate the efficiency of this reaction in presence of disulfides, a small molecule ^1^H-NMR study was performed. The respective linker (15 mM) was combined with 2-mercaptoethanol (30 mM) in an equimolar thiol–ene ratio in D_2_O and irradiated (400–500 nm, 20 mW cm^−2^) in presence of the photoinitiator LiTPO^61,62^ for different time periods (Fig. 1b, Fig. S23b†). A low concentration of LiTPO (0.6 mM, 2 mol% of SH) was used to prevent interaction of excess radicals with disulfides.^39^ The evolution of double bond signals (∼6.0–6.4 ppm, HC=CH) was analysed. The signal of the proton at the α-carbon (∼4.2–4.5 ppm, CH–NH) served as internal reference. Surprisingly, the experiment revealed a strong dependence of the ene-conversion from the molecular linker design (Fig. 1b, Fig. S23†). Although all linkers were expected to efficiently react with a thiol in a thiol–ene “click”-reaction,^56,63^ the results showed that homocysteine-based linkers are highly reactive (Fig. 1b), while the TEC reaction of cystine-based derivatives is strongly retarded (Fig. S23†). Nevertheless, a distinct difference in reactivity among the homocysteine linkers could be found. **Nor-****l****-HCys** reacted much quicker than **Nor-****d**,**l****-HCys** and showed full conversion of norbornenes within 2.5 min of irradiation. With **Nor-****d**,**l****-HCys** high (∼94%) but not quantitative ene-conversion was reached within 10 min. Thus, the TEC reaction of **Nor-****d**,**l****-HCys** seems to be slightly retarded. For comparison, also the non-cleavable linker Nor-O-CC was tested (Fig. 1a and c and Scheme 1). As with **Nor-****l****-HCys**, full conversion was achieved within 2.5 min of irradiation. Since Nor-O-CC contains a non-rigid aliphatic spacer without a distinct configuration of side groups, minimal intramolecular interactions can be assumed. As **Nor-****l****-HCys** shows equal reactivity, minimal intramolecular interactions can be expected as well. Based on these findings, the reactivity of the linkers regarding TEC reaction can be graded in the following order: Nor-O-CC ∼ **Nor-****l****-HCys** > **Nor-****d**,**l****-HCys** ≫ **Nor-****l****-Cys** ∼ **Nor-****d**,**l****-Cys** > ***exo*- Nor-****l****-Cys**.

This simple ^1^H-NMR study demonstrates significant differences in TEC reactivity depending on the molecular design, as recently suggested.^64^ While the radical-mediated TEC reaction has often been considered an archetype of “click”-reactions,^63^ recently several studies pointed out its limitations.^64–67^ It was shown that the efficiency of TEC reactions can be reduced by the nature of the thiol or the ene, the photoinitiator used or the chemical reaction environment. Hence, these studies highlight the importance of carefully optimizing the molecular design in order to achieve high conversion. Yet, no ultimate design guidelines for highly efficient TEC systems could be established yet. Obviously in the current case, both the length of the core as well as its configuration affect the ene-conversion. Hence, intramolecular interactions are expected to influence the reactivity. As the length of the core is critical, since homocystine-based linkers are much more reactive than their cystine counterparts, such interactions most probably occur between functional groups of opposing linker-subunits.

### Computational study

To get a better insight into the cause of the TEC retardation and further evaluate the structure–property relationship, the reaction of the retarded cystine-based linker **Nor-****l****-Cys** (system A) and the elongated, highly-reactive homocystine derivative **Nor-****l****-HCys** (system B) with a 2-mercaptoethanol thiyl radical was computationally modelled (ESI†). System A is more rigid compared to the elongated, and therefore, more flexible system B. As a consequence, system B has a higher degree of freedom for the formation of stabilizing intermolecular interactions and gives the more stabilized thermodynamically product. The thermodynamic driving force of the computationally modeled event is 2.5 times larger for system B than for system A. This agrees well with the experimentally observed reactivity difference for **Nor-****l****-Cys** (A) (Fig. S23†) and **Nor-****l****-HCys** (B) (Fig. 1).

### Development of the disulfide-based hydrogel platform

**Fig. 2:**
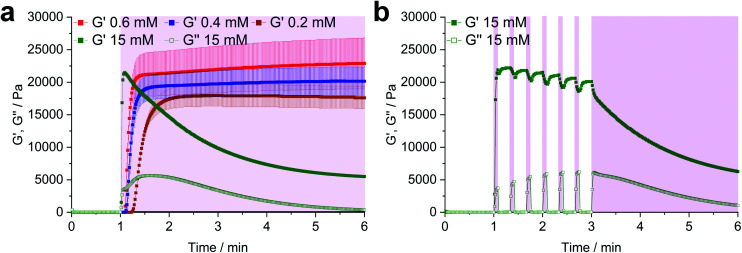
(a) *In situ* oscillatory photorheology of hydrogel formulations containing 10 wt% 8armPEG20k-SH, **Nor-****d**,**l****-HCys** (22.2 mM, SH : ene = 1 : 1) and variable concentrations of LiTPO (0.2 mM, 0.4 mM, 0.6 mM, 15 mM). The prepolymer solutions were exposed to 385 nm light (6 mW cm^−2^) for 5 min continuously, or (b) light was shuttered with 5 s on-time and 15 s off-time followed by 3 min of continuous exposure. Periods of irradiation are highlighted by a purple background.

Next, hydrogel formation of the highly reactive homocystine-based linkers **Nor-****l****-HCys** and **Nor-****d**,**l****-HCys** was investigated by photorheology. Linkers were reacted with 10 wt% of thiol-terminated 8-armed PEG (8armPEG20k-SH, *M*_w_ ∼20 kDa) by irradiation with an LED (385 nm, ∼6 mW cm^−2^). First, the lowest required concentration of LiTPO was established, as an excess of photoinitiator is known to induce degradation of disulfide cross-linked hydrogels.^39^ Stock solutions of 8armPEG20k-SH, **Nor-****d**,**l****-HCys** (190 mM) and LiTPO (18 mM) in PBS were combined to give formulations with final concentrations of 10 wt% 8armPEG20k-SH, 22 mM **Nor-****d**,**l****-HCys** (SH : ene = 1 : 1) and a variable LiTPO concentration (0.2 mM, 0.4 mM, 0.6 mM, 15 mM). After 1 min of equilibration, the prepolymer solutions were irradiated for 5 min (Fig. 2a).

At low LiTPO concentrations (0.2 mM, 0.4 mM, 0.6 mM), a delay of polymerization was observed, which decreases with an increasing amount of photoinitiator, while the final storage modulus *G*′ is increasing from 17.6 ± 1.7 kPa (0.2 mM), over 20.2 ± 2.2 kPa (0.4 mM) to 22.9 ± 3.9 kPa (0.6 mM). Similar behavior has been reported before, when a TEC reaction had been sensitized using different concentrations of Eosin Y.^68^ Anyhow, *G*′ is unaffected by further irradiation after a plateau is reached, showing that crosslinking *via* TEC occurs quicker than degradation of disulfides and LiTPO concentrations of 0.6 mM and below are sufficient for gel formation without provoking significant network degradation.

In contrast, when a high concentration of LiTPO (15 mM) is used, *G*′ rises very rapidly within 5 s to a maximal value of 21.5 ± 0.3 kPa, before declining again. This result illustrates initial very rapid step-growth polymerization followed by network degradation *via* excess photoinitator, again demonstrating that the TEC reaction is preferred over radical-mediated disulfide-cleavage. Here, also the loss modulus *G*′′ is significantly increased during irradiation. As 15 mM LiTPO is not sufficient for complete reverse gelation of a hydrogel based on 22 mM **Nor-****d**,**l****-HCys**, G′ reaches a plateau after 5 min of irradiation due to still intact disulfide-crosslinks. When the light is dynamically shuttered (5 s on-time, 15 s off-time, 3 min continuous irradiation, 15 mM, Fig. 2b, purple shading) the partial reversibility of the radical induced disulfide cleavage becomes apparent.^39^ While during irradiation *G*′ decreases and *G*′′ increases, the inverse behavior is observed when irradiation is interrupted, indicating rapid reformation of disulfide bonds by thiyl radical recombination. However, with every irradiation circle *G*′ is gradually reduced, as thiyl-radicals are consumed by photoinitiator fragments, impeding full restauration of the initial crosslinking density and *G*′_max_. After establishing the impact of the photoinitiator concentration on the hydrogel, further characterizations were performed with a hydrogel formulation based on 10 wt% 8armPEG20k-SH, **Nor-(d,)l-HCys** (22 mM, SH : ene = 1 : 1) and 0.4 mM LiTPO, which will herein be referred to as **8arm-(d,)l-HCys 100** hydrogel.

### Thermo- and mechanostability of 8arm-d,l-HCys 100 hydrogel

To investigate the thermostability, a sample of **8arm-****d**,**l****-HCys 100** hydrogel was photopolymerized at 20 °C on the rheometer, before the temperature was continuously increased up to 90 °C at a heating rate of 1 °C min^−1^ (ESI Fig. S24†). Only at temperatures above 60 °C a sharp decrease of *G*′ could be observed, demonstrating thermal stability of the hydrogel at ambient and physiological conditions. The mechanostability of **8arm-(****d****,)****l****-HCys 100** hydrogel was investigated in a strain sweep experiment, where the strain was logarithmically increased from 0.1% to 1000% at a constant frequency of 1 Hz (ESI Fig. S25†) or dynamically cycled between 1% and 350% (ESI Fig. S26†). Up to a strain of 100% the moduli of the hydrogel remained unaltered. At higher strains rupture of the gel occurred.

### Stabilization of 8arm-d,l-HCys 100 hydrogel

**Fig. 3:**
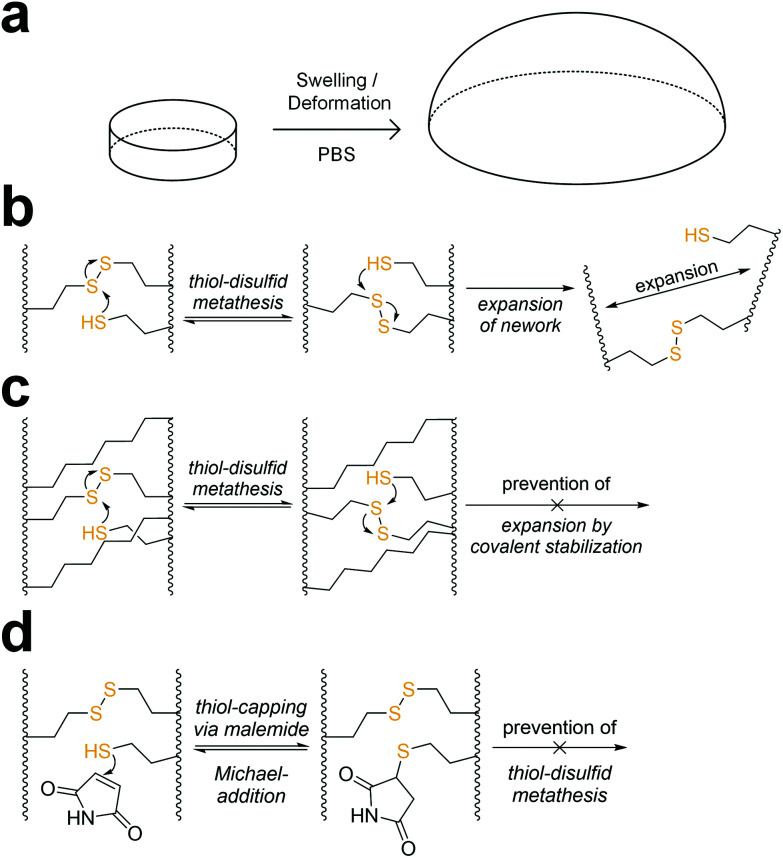
(a) In the course of swelling in PBS, **8arm-****d**,**l****-HCys 100** hydrogel disks lost their shape by turning into spherical caps before finally dissolving within a few days. (b) A possible reason for this behavior is that unreacted thiols can undergo thiol-disulfide metathesis with crosslinks leading to relaxation of internal stress and resulting in expansion of the network. The network could be stabilized by (c) a non-cleavable background network or (d) capping of free thiols by maleimide or an excess of linker.

When immersed in PBS at room temperature, **8arm-****d**,**l****-HCys 100** started swelling heavily, leading to first deformation and then dissolution within a few days. In the course of deformation, cylindrical samples turned into spherical caps (Fig. 3a). This behavior indicates relaxation of surface tension, which is most likely occurring *via* thiol-disulfide metathesis involving free thiols (Fig. 3b). It is in accordance with the observation that partly degraded disulfide networks adapted to an applied strain to minimize the system's free energy.^39^ This is explicable, as the ^1^H-NMR reactivity estimation revealed incomplete ene-conversion of **Nor-****d**,**l****-HCys** (Fig. 1c). Upon swelling and relaxation by thiol-disulfide metathesis the hydrogel expands. Since the linkers contain carboxylate anions to facilitate solubility, further swelling of water into the hydrogel volume is provoked by the increased ion concentration, finally leading to dissolution of the hydrogel.

Interestingly, when swollen in 10x PBS for the first 20 h after formation, **8arm-****d**,**l****-HCys 100** hydrogel remained stable for weeks thereafter (Fig. S27†). Due to the increased ion concentration of the swelling medium in the initial period, the samples were losing mass *via* osmosis first. After the medium was changed to 1× PBS, samples began swelling and gained mass, but remained their shape and did not dissolve. This behavior can be explained by formation of additional crosslinks by unreacted thiols upon condensing of the network due to initial negative swelling.

A strategy to prevent shape-relaxation and expansion of **8arm-****d**,**l****-HCys 100** hydrogel involves the inclusion of non-cleavable aliphatic crosslinks by addition of Nor-O-CC (Fig. 1 and 3c). In order to stabilize a hydrogel based on an 8armPEG20k-SH backbone, at least two arms of the macromer have to be connected to a non-cleavable unit to meet the percolation threshold. Hence, 25% of **Nor-****d**,**l****-HCys** must be substituted by Nor-O-CC to form a non-cleavable background network, in which every macromer is theoretically connected to two units of Nor-O-CC. This hypothesis was confirmed by comparing the swelling behavior of different formulations based on variable ratios of **Nor-****d**,**l****-HCys** to Nor-O-CC. Whereas both non-stabilized **8arm-****d**,**l****-HCys 100** and **8arm-SS-CC (7 : 1)** hydrogel (12.5% Nor-O-CC) were disintegrating within a few days, a mixture of 75% **Nor-****d**,**l****-HCys** and 25% Nor-O-CC gave the stable hydrogel **8arm-SS-CC (3 : 1)**. Further details are given in the ESI (Fig. S28†). This experiment demonstrates that unrestricted expansion of the hydrogel is a reason for dissolution of **8arm-****d**,**l****-HCys 100**. The most probable cause for this expansion is the dynamic reformation of linkages induced by free thiols.

The presence of pendant thiols can be indirectly proven by capping and thus disabling their participation in thiol-disulfide metathesis. As free thiols readily react in a thiol-Michael addition, they can simply be consumed by a small molecule Michael-acceptor (Fig. 3d).^69^ Hence, freshly polymerized samples of **8arm-****d**,**l****-HCys 100** were immersed in a solution of maleimide (4 mM) for 1 h and thereafter swollen in PBS (Fig. S29†). Indeed, this treatment led to a stabilization of the hydrogel, indicating(i) the presence of unreacted thiols as well as (ii) their involvement in the instability of this hydrogel.

**Fig. 4:**
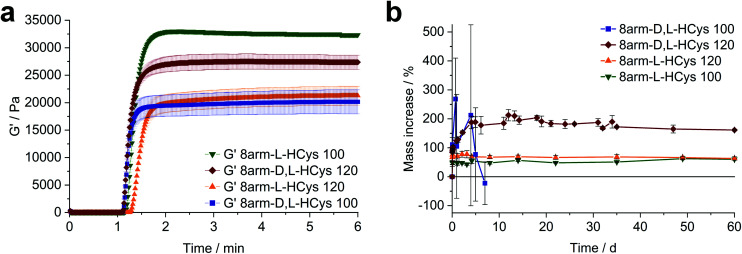
The effect of the configuration of homocysteine-based linkers on hydrogel behavior. (a) Comparison of the photopolymerization of formulations based on 10 wt% 8armPEG20k SH, 0.4 mM LiTPO and linkers with a homocysteine core. When using **Nor-****d**,**l****-HCys** a 20 mol% excess of ene (**8arm-****d**,**l****-HCys 120**) gives a higher storage modulus G′ than in the equimolar case. In contrast, with **Nor-****l****-HCys** the equimolar formulation **8arm-****l****-HCys 100** exhibits the highest *G*′ of the series, whereas an excess of **Nor-****l****-HCys** results in a reduced storage modulus. (b) Swelling behavior of the studied hydrogels in PBS. **Nor-****l****-HCys** gives stable hydrogels at equimolar conditions and when an excess of linker is used. In contrast, with **Nor-****d**,**l****-HCys** and excess of ene is required to yield a stable hydrogel. The data is depicted as mean values of at least triplicates and error bars illustrating the standard deviation.

Another simple yet convenient method to prevent the presences of unreacted thiols is the use of an excess of linker. However, this ene-excess has to be optimized precisely, since it also reduces the overall connectivity of the network, leading to pending norbornenes. Hence, three formulations with increasing ene-excess (15, 20 and 23.5 mol%) were tested by photorheology. Further details can be found in the ESI Fig. S30.† This study could well demonstrate, that (i) with **Nor-****d**,**l****-HCys** ene-conversion is incomplete when reacted at an 1 : 1 ratio of functional groups. Moreover, (ii) the crosslinking density and stiffness can even be increased by using an excess of linker, whereby an optimum excess of ene could be established at 20 mol% (**8arm-****d**,**l****-HCys 120**, Fig. 4a and b, ESI Fig. S30†).

### Nor-l-HCys based hydrogels

**Nor-** **l** **-HCys** showed the highest reactivity of all investigated linkers and exhibited quantitative conversion of ene in the model reaction with 2-mercaptoethanol within 2.5 min of irradiation, whereas **Nor-****d**,**l****-HCys** reached ∼96% conversion only after 10 min of irradiation (Fig. 1c). This leads to remarkable behavior in corresponding rheological and swelling experiments, when comparing hydrogel formulations based on these two linkers (Fig. 4a). While formulations with **Nor-****l****-HCys** react slightly slower, they reach higher *G*′ values than their **Nor-****d**,**l****-HCys** counterparts. More importantly, whereas with **Nor-****d**,**l****-HCys** a 20 mol% excess of ene is required to reach maximum *G*′ (**8arm-****d**,**l****-HCys 120**, 27.4 ± 1.3 kPa) and form a stable hydrogel (Fig. 4b), with **Nor-****l****-HCys** the equimolar formulation exhibits the highest *G*′ of the series (**8arm-****l****-HCys 100**, 32.4 ± 0.3 kPa). In contrast, using a 20% excess of ene leads to a lower stiffness when **Nor-****l****-HCys** is used (**8arm-****l****-HCys 120**, 21.3 ± 1.7 kPa). It lies in the range of the equimolar formulation of **Nor-****d**,**l****-HCys** (**8arm-****d**,**l****-HCys 100**, 20.2 ± 2.2 kPa).

These results indirectly confirm the very high to quantitative ene-conversion of **Nor-****l****-HCys** observed in the ^1^H-NMR reactivity estimation. The equimolar formulation of **Nor-****l****-HCys** reaches a higher storage modulus than when a 20 mol% excess of ene is used. In contrast, with **Nor-****d**,**l****-HCys**, an excess of ene leads to maximum *G*′. This is also reflected in the swelling behavior, since hydrogels of both **Nor-****l****-HCys** formulations remain stable, whereby the equimolar formulation exhibits the lowest swelling of the series, again indicating high network connectivity (Fig. 4b).

In the case of the linker **Nor-****d**,**l****-HCys**, the final storage modulus is reached within less than 60 s of irradiation (Fig. 4a and Fig. S30†), while in the NMR experiments it takes 2.5 min to reach a norbornene conversion of 80%, and only after 10 min a high ene-conversion of 96% is obtained. We hence reason that this lower reactivity of **Nor-****d**,**l****-HCys** compared to **Nor-****l****-HCys** is the cause why an excess of linker is required here to form a stable hydrogel.

### Two-photon micropatterning of disulfide-crosslinked hydrogels

**Fig. 5:**
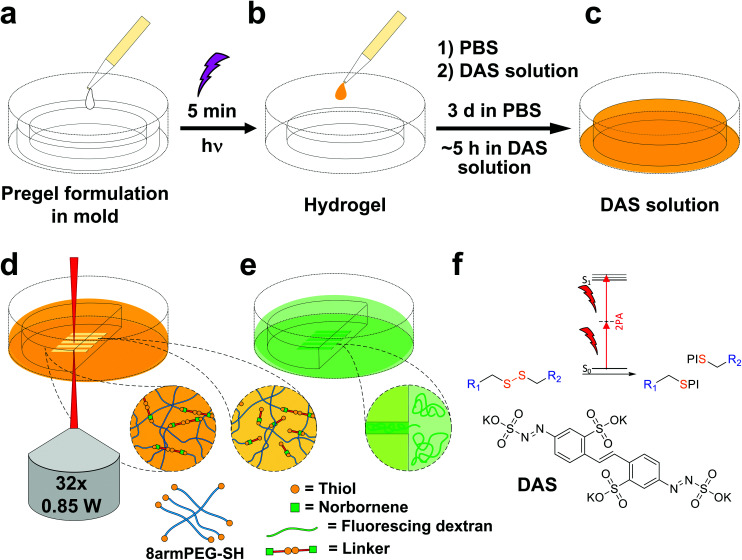
Workflow of hydrogel formation *via* UV-light and treatment with **DAS** prior to two-photon micropatterning and post-treatment for visualization. (a) Disulfide-crosslinked PEG hydrogels were formed by photoinduced TEC upon irradiation with UV-light. (b) Thereafter, hydrogels were swollen in PBS, (c) before being soaked in solutions of **DAS**. (d) The activated hydrogels were then two-photon micropatterned using a fs-pulsed laser. (e) Microchannels were visualized by confocal microscopy, after swelling in fluorescing dextran. (f) The presence of the two-photon active **DAS** is crucial for effective cleavage of disulfide linkages by two-photon irradiation. PI denotes a photoinitiator fragment.

**Fig. 6:**
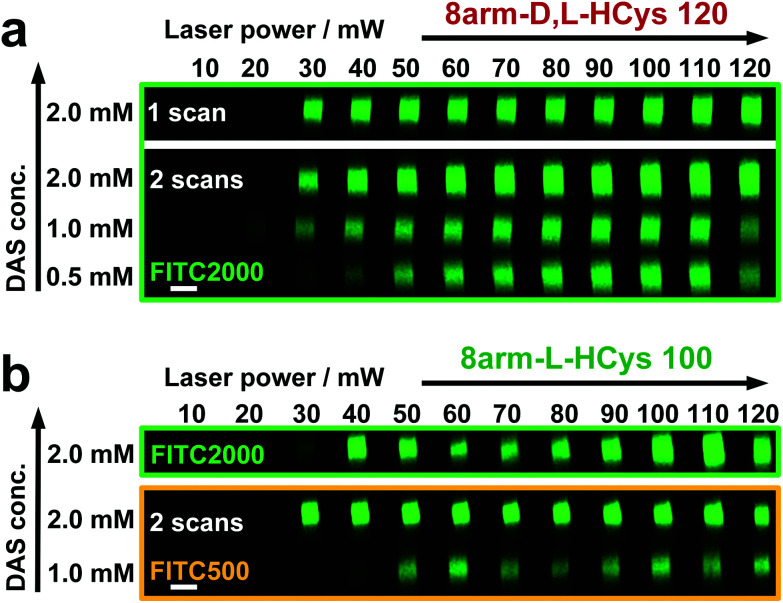
Microchannels were fabricated by two-photon degradation of disulfide-based hydrogels in presence of two-photon initiator **DAS** (0.5–2.0 mM). Individual *x*,*y*-planes were either scanned once or twice. Thereafter, hydrogels were soaked in a solution of high molecular weight fluorescent dextran (**FITC2000**) and microchannels were visualized by confocal microscopy. (a) **8arm-****d**,**l****-HCys 120** could be fully degraded in presence of 2.0 mM **DAS** in a single scan. At lower concentration double scanning was required for complete erosion. (b) Microchannel fabrication in stiffer **8arm-****l****-HCys 100** hydrogel required double scanning in presence of 2.0 mM **DAS** for complete erosion. When 1 mM **DAS** was used, channels could only be visualized using lower molecular weight dextran (**FITC500**), indicating localized softening of the material. Images display orthogonal cross sections (*y,z*-plane) of micro channels. Scale bar 20 μm.

Disulfide crosslinked hydrogels can be degraded by UV-light in presence of a cleavable photoinitiator *via* a radical-mediated disulfide fragmentation.^39^ Here, we demonstrate that this concept also works in the two-photon irradiation regime, permitting 3D-micropatterning of such hydrogels. For this purpose, the cleavable, water-soluble two-photon initiator **DAS** was used (Fig. 5f).^70,71^ Two-photon induced hydrogel cleavage was studied by microchannel formation and subsequent visualization of the channels *via* confocal microscopy, after swelling with high molecular weight fluorescent dextran.^18^ Micropatterning was tested on **8arm-****d**,**l****-HCys 120** and **8arm-****l****-HCys 100** hydrogels. Samples were formed in molds by irradiation at 365 nm, and first swollen in PBS to reach equilibrium swelling and subsequently submersed in solutions of **DAS** at different concentrations (0.5 mM, 1.0 mM, 2.0 mM), to permit diffusion of the chromophore into the network (Fig. 5a–c). Thereafter, the samples were micropatterned at a scanning speed of 200 mm s^−1^ using a fs-pulsed laser (720 nm) focused through a water immersion objective (Fig. 5d). Parallel channels were eroded into the hydrogel at laser powers ranging from 10–120 mW. Individual *x*,*y*-planes were either scanned once or twice. For examination of the channels by LSM, the samples were swollen with high molecular weight fluorescent dextran (2000 kDa, **FITC2000**). **FITC2000** only infiltrates open micro-channels but not the bulk hydrogel (Fig. 5e). When **8arm-****d**,**l****-HCys 120** hydrogel was micropatterned using 2.0 mM **DAS**, open channels formed after a single scan at laser powers starting from 20 mW (Fig. 6a, ESI Fig. S31†). At lower concentrations it was necessary to scan each *x*,*y*-plane twice to produce open microchannels. At a concentration of 1 mM **DAS** bright fluorescence was visible in regions scanned at 30 mW and above, while at 0.5 mM **DAS** at least 40 mM were required to produce channels. When no **DAS** was used, no fluorescence could be observed in scanned regions. This experiment demonstrates that the two-photon cleavage requires the use of a two-photon active initiator and that the threshold for this cleavage is concentration dependent.

The scanning parameters for complete degradation can be optimized in multiple ways, as magnification, laser power, scanning speed, hatch and *z*-layer spacing influence the applied light dose. Here, a hatch distance of 0.1 μm and a *z*-layer spacing of 0.5 μm was used. At **DAS** concentrations below 2 mM, *x*,*y*-planes had to be scanned twice. In contrast, for photodegradation of a hydrogel based on an oNB ester moiety, which undergoes defined molecular photolysis in response to pulsed light (*λ* = 740 nm), 5–7 frame repeat scans using 1.9 W laser power with a pixel dwell time of 2 μs were reported to be required recently.^17^ However, no additional oNB-cleavage-promoting two-photon sensitizer^18^ was used in these experiments.

When using the same scanning parameters as above on the stiffer **8arm-****l****-HCys 100** hydrogel, double scanning in presence of 2.0 mM **DAS** was required to fabricate micro-channels at laser powers of 40 mW and above, which could be infiltrated by **FITC2000** (Fig. 6b, ESI Fig. S32†). With lower molecular weight FITC-dextran (500 kDa, **FITC500**) channels fabricated at 30 mW were visible as well. When only 1.0 mM **DAS** was used in **8arm-****l****-HCys 100**, microchannels produced by double scanning at laser powers of 50 mW and above required swelling in **FITC500** for visualization, indicating local softening but no complete degradation at these scanning parameters. In contrast, when channels were only scanned once in presence of 2.0 mM **DAS**, hydrogel degradation was not sufficient for FITC-dextran to enter the irradiated areas and no fluorescence could be observed (Fig. S33†).

With these experiments, two-photon cleavage of disulfides in presence of **DAS** could be demonstrated. Based on the scanning parameters used either complete degradation or localized softening of hydrogels can be achieved, depending on the application of choice. Moreover, with the developed linker photodegradable hydrogels can be produced directly from thiol-terminated macromers (thiol-gelatin,^72^ thiol-HA,^49^ thiol-PEG,^39^ thiol-PVA^73^) without the use of linkers containing photolabile groups, significantly simplifying precursor preparation and easing handling of hydrogels, as no inherent light sensitivity is given prior to the addition of a photoinitiator.

Noteworthy, others have developed a photoresist based on thiol-terminated macromers and a phenacyl sulfide linker for the direct formation of disulfide networks upon two-photon irradiation at 700 nm. In this system the phenacyl sulfide releases a photo-caged thioaldehyde species, which reacts with a thiol to form a disulfide bond.^74^ Hence, our two-photon induced cleavage of disulfide networks also expands the methodology for localized 3D-printing and degradation of polymer networks by means of two-photon irradiation, as recently shown by Batchelor *et al.*^75^

## Conclusion

A disulfide-crosslinker for the redox-free, photochemical formation of cleavable hydrogels from thiol-terminated precursors *via* TEC reaction was developed. Interestingly, the reactivity of the TEC reaction was found to be strongly dependent on the molecular design of the linker. Whereas linkers based on homocysteine were highly reactive, those with a cystine core exhibited hindered thiol–norbornene addition. However, full consumption of free thiols is crucial. It could be shown that pendant thiol moieties lead to an unstable network and hence have to be avoided. In the absence of free thiols, disulfide-based hydrogels formed from a thiol-terminated macromer were stable for months in PBS. Furthermore, a method for two-photon induced degradation of disulfide-based hydrogels was demonstrated by microchannel fabrication. The presented crosslinker extends the scope of photolabile functional groups, as it is easily accessible and allows the mild formation of various disulfide-based cleavable networks in a controllable way by the use of light. Due to the reversible nature of the disulfide bond, it provides various opportunities for the design of tunable materials systems with tailored properties, allowing to engineer materials for numerous applications from tissue engineering to drug delivery, microfluidics and materials science.

## Author contributions

The manuscript was written through contributions of all authors. All authors have given approval to the final version of the manuscript.

## Conflicts of interest

The authors declare no competing financial interest.

## Supplementary Material

PY-013-D1PY00914A-s001
