# Supplementary material for: A disulfide-based linker for thiol–norbornene conjugation: formation and cleavage of hydrogels by the use of light
Source: Polym Chem. 2022 Jan 10;13(9):1158–68. doi: 10.1039/d1py00914a (PMC8886483; doi:10.1039/d1py00914a)
Supplement: PY-013-D1PY00914A-s001 [file PY-013-D1PY00914A-s001.pdf]

Electronic Supplementary Material (ESI) for Polymer Chemistry.
This journal is © The Royal Society of Chemistry 2022

Supporting Information

A disulfide-based linker for thiol-norbornene conjugation: formation and cleavage of hydrogels by the use of light

Markus Lunzer,[a,b] Boris Maryasin,[c,d] Tommaso Zandrini,[b] Stefan Baudis,[a] Aleksandr Ovsianikov,[b] Robert Liska[a,*]

a. Institute of Applied Synthetic Chemistry, TU Wien, 1060 Vienna, Austria

b. Institute of Materials Science and Technology, TU Wien, 1060 Vienna, Austria

c. Institute of Organic Chemistry, University of Vienna, 1090 Vienna, Austria

d. Institute of Theoretical Chemistry, University of Vienna, 1090 Vienna, Austria

E-mail: robert.liska@tuwien.ac.at

Abstract: Photolabile groups are the key components of photo-responsive polymers, dynamically tunable materials with multiple applications in materials and life sciences. They usually consist of a chromophore and a labile bond and are inherently light sensitive. An exception are disulfides, simple reversible linkages, which become photocleavable upon addition of a photoinitiator. Despite their practical features, disulfides are rarely utilized due to their impractical formation. Here, we report a disulfide-based linker series bearing norbornene terminals for facile crosslinking of thiol-functionalized macromers via light-triggered thiol-ene conjugation (TEC). Besides finding a highly reactive lead compound, we also identify an unexpected TEC-retardation, strongly dependent on the molecular linker structure and affecting hydrogel stability. Finally, we present a useful method for localized disulfide cleavage by two-photon irradiation permitting micropatterning of disulfide-crosslinked networks.

Materials 4
Instrumentation 4
Synthetic Procedures 5
Synthesis of 2,2'-(1,6-hexanediyl)bicyclo[2.2.1]hept-5-ene-2,3-dicarboxylic acid ester, Nor-O-CC 5
Synthesis of sodium 2,2'-(1,6-hexanediyl) bicyclo[2.2.1]hept-5-ene-2,3-dicarboxylic acid ester, Nor-O-CC Na salt 6
Synthesis of bicyclo[2.2.1]hept-5-ene-2-carbonyl chloride, NorCOCl 7
Synthesis of exo-5-norbornenecarboxylic acid chloride, exo-NorCOCl 7
General procedure for the preparation of (homo)cystine-based linkers 8
General procedure for the conversion of (homo)cystine-based linkers into the salt form 8
Synthesis of disodium *N,N'*-bis(bicyclo[2.2.1]hept-5-ene-2-ylcarbonyl)-L-cystine, Nor-L-Cys Na salt 9
Synthesis of disodium *N,N'*-bis-(*exo*-bicyclo[2.2.1]hept-5-ene-2-ylcarbonyl)-L-cystine, exo-Nor-L-Cys Na salt 10
Synthesis of disodium *N,N'*-bis(bicyclo[2.2.1]hept-5-ene-2-ylcarbonyl)-D,L-cystine, Nor-D,L-Cys Na salt 11
Synthesis of disodium *N,N'*-bis(bicyclo[2.2.1]hept-5-ene-2-ylcarbonyl)-L-homocystine, Nor-L-HCys Na salt 12
Synthesis of disodium *N,N'*-bis(bicyclo[2.2.1]hept-5-ene-2-ylcarbonyl)-D,L-homocystine, Nor-D,L-HCys Na salt 13
Synthesis of disodium 1,1'-dimethyl-*N,N'*-bis(bicyclo[2.2.1]hept-5-ene-2-ylcarbonyl)-L-cystine ester, Nor-L-CysMe 14
Synthesis of 3,3'-(((disulfanediylbis(ethane-2,1-diyl))bis(oxy))bis(carbonyl))-bis-(bicyclo[2.2.1]hept-5-ene-2-carboxylic acid), Nor-O-SS 15
Synthesis of sodium 3,3'-(((disulfanediylbis(ethane-2,1-diyl))bis(oxy))-bis(carbonyl))-bis(bicyclo[2.2.1]hept-5-ene-2-carboxylate), Nor-O-SS Na salt 16
Experimental Procedures 17
Linker reactivity estimation by ^{1}H-NMR in D_2O. 17
Linker reactivity estimation by ^{1}H-NMR in DMSO-d_6. 17
Photorheology. 17
Hydrogel swelling experiments. 18
Optimization of the photoinitiator concentration 18
Thermostability of 8arm-D,L-HCys 100 hydrogel 18
Mechanostability of 8arm-D,L-HCys 100 hydrogel 19
Stabilization of 8arm D,L-HCys 100 hydrogel by a non-cleavable background network 19
Stabilization of 8arm D,L-HCys 100 hydrogel by capping of free thiols by maleimide 19
Consumption of free thiols by an excess of Nor-D,L-HCys linker 19
Stabilization of 8armPEG-based hydrogels by the use of Nor-L-HCys 19
Two-photon micropatterning. 20

Supporting Figures 21
Computational study 41
Computational details 44
References 45
Appendix 46

MATERIALS

All photosensitive steps were performed under light protection. Unless otherwise stated, chemicals were purchased from Sigma-Aldrich, TCI Europe or ABCR GmbH and used without further purification. Thiol-terminated 8-armed PEG (8armPEG20k-SH, Mw ~20 kDa, tripentaerythritol core) was purchased from JenKem Technology USA Inc. The photoinitiators LiTPO (lithium phenyl-2,4,6-trimethylbenzoylphosphinate) and **DAS** (tetrapotassium 4,4′-(1,2-ethenediyl)bis[2-(3-sulfophenyl)diazenesulfonate]) were synthesized at TU Wien following literature procedures.[1, 2] TLC aluminum foils coated with silica gel 60 F254 from Merck KGaA, (Darmstadt, Germany) were used. Column chromatography was performed on Merck silica gel 60 (0.040-0.063 mm). Fluorescein-modified dextran (FITC–dextran) was purchased from TdB Consultancy AB (Uppsala, Sweden).

INSTRUMENTATION

NMR spectra were recorded at 600 MHz for ^{1}H and 150 MHz for ^{13}C on a Bruker Avance III HD spectrometer and at 400 MHz for 1H and 100 MHz for ^{13}C on a Bruker Avance DRX-400 spectrometer. Data for ^{1}H-NMR are reported as follows: chemical shift in units of parts per million (ppm) from tetramethylsilane (TMS) using the residual non-deuterated solvent signal of $CDCl_3$ (δ_H = 7.26 ppm), D_2O (δ_H = 4.79 ppm) or DMSO-d_6 (δ_H = 2.50 ppm) as internal reference. Multiplicities are reported by using the following abbreviations; bs: broad singlet; s: singlet; d: doublet; t: triplet; q: quartet; quint: quintet, m: multiplet; J: coupling constants in Hertz (Hz). ^{13}C-NMR data are reported in ppm from TMS using the central peak of the solvent as reference ($CDCl_3$: δ_C = 77.16 ppm, DMSO-d_6: δ_C = 39.51 ppm); the multiplicity with respect to H (s = quaternary C, d = CH, t = CH_2, q = CH_3) is deduced from attached proton test (APT) experiments.

HRESIMS analysis was carried out from methanol or acetonitrile solutions (concentration: 10 ppm) using an HTC PAL system autosampler (CTC Analytics AG, Zwingen, Switzerland), an Agilent 1100/1200 HPLC with binary pumps, degasser and column thermostat (Agilent Technologies, Waldbronn, Germany) and Agilent 6230 AJS ESI–TOF mass spectrometer (Agilent Technologies, Palo Alto, USA).

Column chromatography was performed with a Büchi MPLC-system equipped with the control unit C-620, fraction collector C-660, and UV-photometer C-635 (BÜCHI Labortechnik AG, Flawil, Switzerland).

SYNTHETIC PROCEDURES

Synthesis of 2,2'-(1,6-hexanediyl)bicyclo[2.2.1]hept-5-ene-2,3-dicarboxylic acid ester, Nor-O-CC

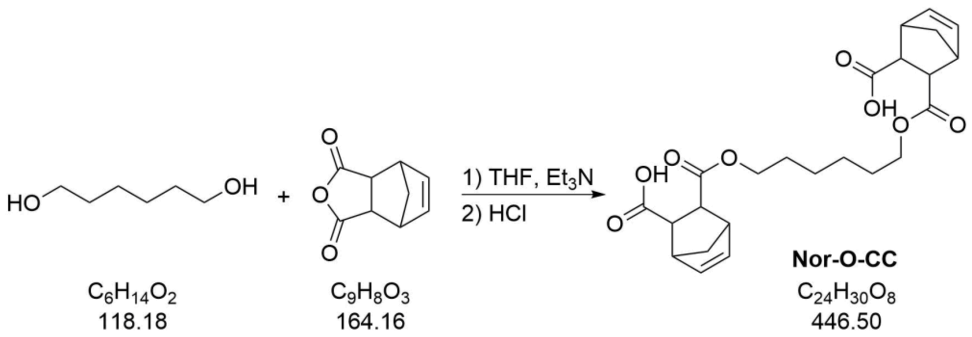

The synthesis of bicyclo[2.2.1]hept-5-ene-2,3-dicarboxylic acid, 2,2'-(1,6-hexanediyl) ester **Nor-O-CC** was conducted according to a modified procedure.[3]

1,6-Hexanediol (1.43 g, 12 mmol, 1 eq) was dissolved in dry THF (20 mL) in Ar atmosphere. A solution of carbic anhydride (3.97 g, 24 mmol, 2 eq) in dry THF (25 mL) was added to the stirred solution. Thereafter, trimethylamine (6.25 mL, 48 mmol, 4 eq) was added slowly and the reaction was stirred at room temperature. After 14 h of stirring, ^{1}H-NMR showed 80% consumption of starting materials. Hence, the reaction mixture was refluxed for 3 h to increase the conversion. The solvent was removed in vacuum and the white residue was dissolved in sat. aq. $NaHCO_3$ solution (50 mL) to give a “clear” solution. The product was precipitated using conc. HCl giving a sticky white solid. The aqueous phase was extracted with diethyl ether (3 x 200 mL) and the combined organic layers were washed with brine. The organic phase was dried over Na_2SO_4 and the solvent was removed in vacuum yielding a whitish oil. The raw product was purified by column chromatography (110 g silica) using DEE: ethyl acetate = 50% as eluent. To remove residual monosubstituted derivative, it was recrystallized from toluene twice yielding a white solid (4.23 g, 79%).

^{1}H-NMR (400 MHz, DMSO-d_6): δ = 11.89 (s, 2H, -COOH), 6.15-6.13 (dd, J = 2.9 Hz, 2.9 Hz, 2H, CH=CH), 6.10-6.06 (dd, J = 2.9 Hz, 2.9 Hz, 2H, CH=CH), 3.94-3.79 (m, 4H, O-CH_2-CH_2), 3.29-3.21 (m, 4H, aliphatic), 3.03-3.00 (m, 4H, aliphatic), 1.52-1.49 (m, 4H, aliphatic), 1.34-1.25 (m, 8H, aliphatic) ppm.

^{13}C-NMR (100 MHz, DMSO-d_6): δ = 173.2 (s, COOH), 172.0 (s, $COOCH_2$), 135.0 (d, CH=CH), 134.3 (d, CH=CH), 63.5 (t, $COOCH_2$), 47.8 (t, CH-CH_2-CH), 47.3 (d, $CHCOOCH_2$), 47.1 (d, CHCOOH), 45.9 (d, CHCH-ester), 45.6 (d, CHCH-acid), 27.9 (t, aliphatic), 25.1 (t, aliphatic) ppm.

HRMS (ESI): $[M + Na]^+$ m/z calcd. for $C_{24}H_{30}O_8Na^+$ 469.1833; found 469.1844.

Synthesis of sodium 2,2'-(1,6-hexanediyl) bicyclo[2.2.1]hept-5-ene-2,3-dicarboxylic acid ester, Nor-O-CC Na salt

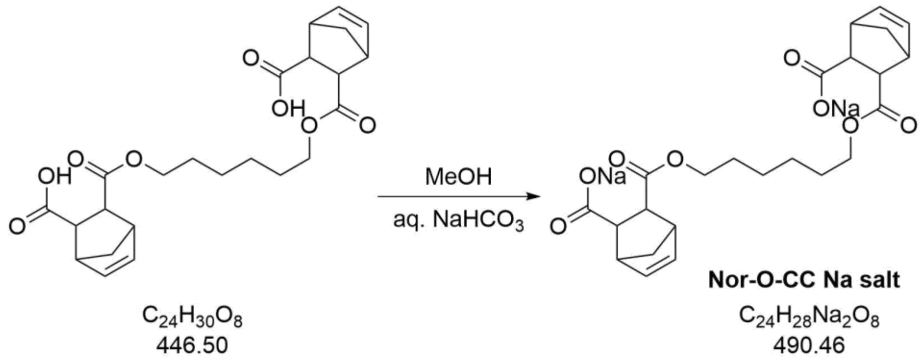

Nor-O-CC (4.1 g, 9.2 mmol, 1 eq) was dissolved in MeOH (20 mL) and an aqueous solution of $NaHCO_3$ (1 M, 18.4 mL, 18.4 mmol, 2 eq) was added dropwise to a stirred solution. Formation of gas was observed. The solution was stirred for 15 min, the solvent was evaporated and the solid residue was then dried in high vacuum for two days yielding a transparent glass (4.5 g, quant.).

^{1}H-NMR (400 MHz, D_2O): δ = 6.29-6.27 (dd, J = 2.9 Hz, 2.6 Hz, 2H, C<u>H</u>=CH), 6.22-6.20 (dd, J = 2.9 Hz, 2.6 Hz, 2H, CH=C<u>H</u>), 4.10-4.05 (m, 2H, O-C<u>H</u>H-CH_2), 4.00-3.94 (m, 2H, O-CH<u>H</u>-CH_2), 3.38-3.13 (m, 8H, aliphatic), 1.63-1.62 (m, 4H, aliphatic), 1.45-1.38 (m, 8H, aliphatic) ppm.

Synthesis of bicyclo[2.2.1]hept-5-ene-2-carbonyl chloride, NorCOCl

For practical and economic reasons, **NorCOCl** was synthesized from 5-norbornene-2-carboxylic acid in its common commercial form, a racemic mixture of exo- and endo-isomers. Consequently, the resulting linkers were obtained as a mixture of diastereomers and used as received without applying complex purification procedures.

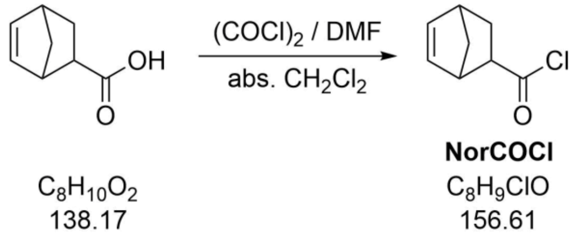

The synthesis was conducted according to a protocol by Chae *et al*.[4]

5-Norbornene-2-carboxylic acid (14 g, 101 mmol, 1 eq, CAS: 120-74-1, mixture of exo-/endo, predominantly endo) was dissolved in dry DCM (112 mL) in Ar atmosphere and a small droplet of DMF was added. Oxalyl chloride (51 g, 405 mmol, 4 eq) was added to a one-neck flask with a dropping funnel attached and the apparatus was purged with Ar for 5 min. The carboxylic acid solution was added dropwise via the dropping funnel to the stirred oxalyl chloride (gas formation). After the reaction was completed (^{1}H-NMR), remaining oxalyl chloride and DCM were removed under reduced pressure. The product NorCOCl was obtained as brownish liquid (15.3 g, 98 mmol, 97%). Since the ^{1}H-NMR revealed high purity, the product was used without further purification.

^{1}H-NMR (400 MHz, $CDCl_3$): δ = 6.27-6.20 (m, 1H, CH=C<u>H</u>), 6.13-6.02 (m, 1H, C<u>H</u>=CH), 3.47-2.72 (m, 3H), 2.04-1.91 (m, 1H), 1.54-1.41 (m, 2H), 1.34-1.32 (m, 1H) ppm.

Synthesis of exo-5-norbornenecarboxylic acid chloride, exo-NorCOCl

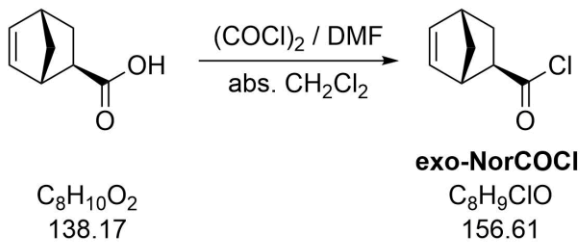

exo-5-Norbornene-2-carboxylic acid (1.00 g, 7.2 mmol, 1 eq, CAS: 934-30-5) was reacted with oxalyl chloride (3.67 g, 29.0 mmol, 4 eq) in presence of a droplet of DMF in dry DCM (8.0 mL) in Ar atmosphere until full conversion was achieved. Remaining oxalyl chloride and DCM were removed under reduced

pressure. The obtained brown liquid was purified by distillation at 15 mbar, T = 70-75 °C). exo-NorCOCl was obtained as a clear translucent liquid (0.96 g, 85%).

^{1}H-NMR (400 MHz, $CDCl_3$): δ = 6.22-6.20 (m, 1H, CH=C<u>H</u>), 6.13-6.11 (m, 1H, C<u>H</u>=CH), 3.28 (m, 1H, =CH-C<u>H</u>-CH-COCl), 2.99 (m, 1H, =CH-C<u>H</u>), 2.75-2.72 (m, 1H, CH-COCl), 2.05-2.00 (m, 1H, aliphatic), 1.55-1.41 (m, 3H, aliphatic) ppm.

General procedure for the preparation of (homo)cystine-based linkers

The general procedure is roughly based on a modified synthetic procedure.[5]

Cystine or Homocystine (1 eq) and finely ground NaOH (4 eq) were added to a one-neck flask and purged with Ar. Dry MeOH was added while stirring until a clear solution was obtained. Thereafter, the stirred reaction mixture was cooled with an ice bath and freshly distilled norbornene acid chloride (2 eq) was slowly added. After 1 h the ice bath was removed and stirring was continued at room temperature until complete conversion was observed by ^{1}H-NMR reaction monitoring. MeOH was removed under reduced pressure. The obtained solid typically smelled heavily of methyl 5-norbornene-2-carboxylate (NorCOOMe). The solid was washed three times with DCM, DEE or ethyl acetate to remove most of NorCOOMe. The product was dissolved in deionized H_2O and 1 M HCl was added until pH 1 was obtained. The protonated product precipitated as white to yellow solid and was obtained by dissolving the precipitate with DEE or ethyl acetate and extraction of the remaining aqueous phase.

The combined organic layers where washed with brine, dried with Na_2SO_4 and filtered before the solvent was removed under reduced pressure. The obtained solid was washed with DCM twice to remove remaining NorCOOMe and then dried in HV. For removing of remaining solvent traces the product can be carefully heated with warm air while on HV.

General procedure for the conversion of (homo)cystine-based linkers into the salt form

For the transformation into its water-soluble Na salt, the respective linker was dissolved in MeOH until a clear solution was obtained. A solution of $NaHCO_3$ in water (1 M, 2 eq.) was slowly added to the stirred linker solution causing bubble formation. After 15 min of stirring, solvents were removed under reduced pressure. The obtained solid was further dried under high vacuum for several hours.

Synthesis of disodium *N,N'*-bis(bicyclo[2.2.1]hept-5-ene-2-ylcarbonyl)-L-cystine, Nor-L-Cys Na salt

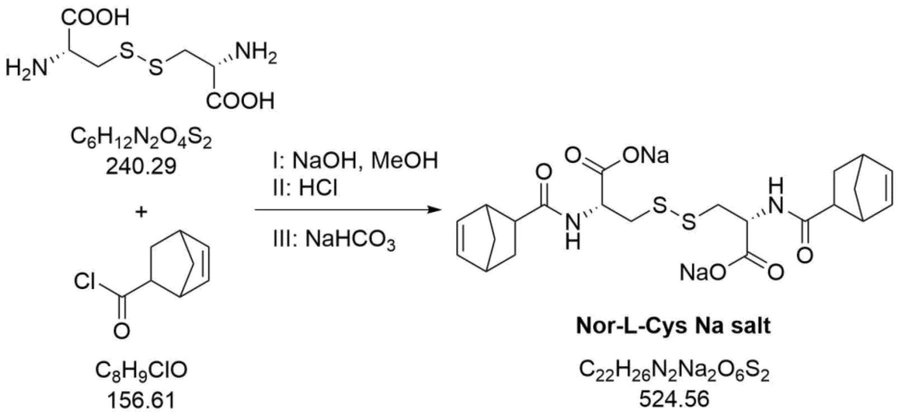

The synthesis of **Nor-L-Cys** is roughly based on a modified procedure.[5]

Powdered NaOH (640 mg, 16.0 mmol, 2.5 eq) was first dissolved in dry MeOH (200 mL) before *L*-cystine (1538 mg, 6.4 mmol, 1 eq) was added in argon atmosphere. To the cooled solution (ice bath) **NorCOCl** (2506 mg, 16.0 mmol, 2.5 eq) was added dropwise. After 20 h of reaction time, the solvent was removed under reduced pressure. The yellow residue was first washed with diethyl ether (3 x 100 mL) to remove NorCOMe. Subsequently, it was dissolved in deionized water (100 mL) and acidified with 1 N HCl. The aqueous phase was extracted with diethyl ether (5 x 200 mL). The combined organic layers were washed with brine, dried with Na_2SO_4 and the solvent was removed in vacuum yielding **Nor-L-Cys** (2338 mg, 76%).

For conversion into the Na salt, **Nor-L-Cys** (1425 mg, 3.0 mmol, 1 eq) was dissolved in MeOH (20 mL) and aqueous $NaHCO_3$ solution (1 M, 6.0 mL, 6.0 mmol, 2 eq) was added. The mixture was stirred for 15 min and then dried in vacuum yielding 1555 mg (quant.) of a beige powder.

Nor-L-Cys:

^{1}H-NMR (400 MHz, DMSO-d$_6$): δ = 12.79 (bs, 2H, COO<u>H</u>), 8.31-7.96 (m, 2H, N<u>H</u>), 6.13-5.81 (m, 4H, <u>H</u>C=C<u>H</u>), 4.48-4.37 (m, 2H, C<u>H</u>-NH), 3.21-3.08 (m, 4H, aliphatic), 2.98-2.80 (m, 6H, aliphatic), 1.81-1.25 (m, 8H, aliphatic) ppm.

HRMS (ESI): $[M + H]^+$ m/z calcd. for $C_{22}H_{29}N_2O_6S_2^+$ 481.1462; found 481.1472.

Nor-L-Cys Na salt:

^{1}H-NMR (400 MHz, D_2O): δ = 6.28-5.98 (m, 4H, <u>H</u>C=C<u>H</u>), 4.55-4.42 (m, 2H, C<u>H</u>-NH), 3.33-3.19 (m, 4H, aliphatic), 3.09-2.95 (m, 6H, aliphatic), 2.01-1.75 (m, 2H, aliphatic), 1.60-1.24 (m, 6H, aliphatic) ppm.

Synthesis of disodium *N,N'*-bis-(*exo*-bicyclo[2.2.1]hept-5-ene-2-ylcarbonyl)-L-cystine, exo-Nor-L-Cys Na salt

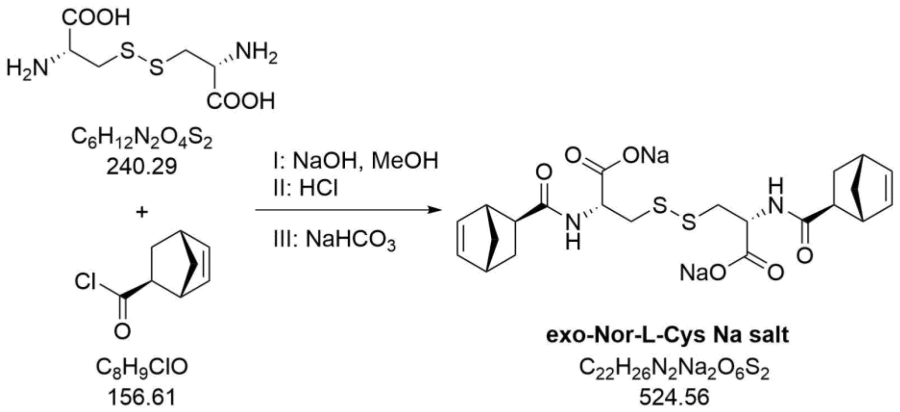

exo-Nor-L-Cys was prepared according to the general procedure from *L*-cystine (705 mg, 3 mmol, 1 eq), **exo-NorCOCl** (918 mg, 6 mmol, 2 eq), NaOH (465 mg, 12 mmol, 4 eq) and dry MeOH (43 mL). After 17 h of reaction time, 1038 mg (72%) of crude **exo-Nor-L-Cys** were received as white to yellow powder. 227 mg (0.43 mmol) of crude **exo-Nor-L-Cys** were converted into the Na salt by dissolving in 25 mL MeOH and adding to $NaHCO_3$ solution (1 M, 945 µL) yielding 248 mg (quant.) of a beige powder after drying in high vacuum. The remaining **exo-Nor-L-Cys** was purified via reversed phase chromatography (80 g Sepacore® C18, Büchi) using MeOH:H_2O = 1:1 as eluent yielding a white to slightly beige powder (65% recovery).

exo-Nor-L-Cys:

^{1}H-NMR (400 MHz, DMSO-d$_6$): δ = 12.90 (bs, 2H, COOH), 8.31-8.22 (m, 2H, NH), 6.13 (m, 4H, HC=CH), 4.56-4.44 (m, 2H, CH-NH), 3.20-3.12 (m, 2H, =CH-CH-CH-CON), 2.94-2.88 (m, 2H, =CH-CH), 2.83 (m, 4H, S-CH$_2$), 2.11-2.10 (m, 2H, CH-CON), 1.80-1.77 (m, 2H, aliphatic), 1.58-1.56 (m, 2H, aliphatic), 1.16-1.15 (m, 4H, aliphatic) ppm.

HRMS (ESI): $[M + Na]^+$ m/z calcd. for $C_{22}H_{28}N_2O_6S_2Na^+$ 503.1281; found 503.1287.

exo-Nor-L-Cys Na salt:

^{1}H-NMR (400 MHz, D_2O): δ = 6.22 (m, 4H, HC=CH), 4.57-4.50 (m, 2H, CH-NH), 3.32-3.19 (m, 2H, =CH-CH-CH-CO), 3.01-2.95 (m, 6H, aliphatic), 2.26 (m, 2H, aliphatic), 1.84-1.75 (m, 2H, aliphatic), 1.54-1.52 (m, 2H, aliphatic), 1.46-1.34 (m, 4H, aliphatic) ppm.

Synthesis of disodium *N,N'*-bis(bicyclo[2.2.1]hept-5-ene-2-ylcarbonyl)-D,L-cystine, Nor-D,L-Cys Na salt

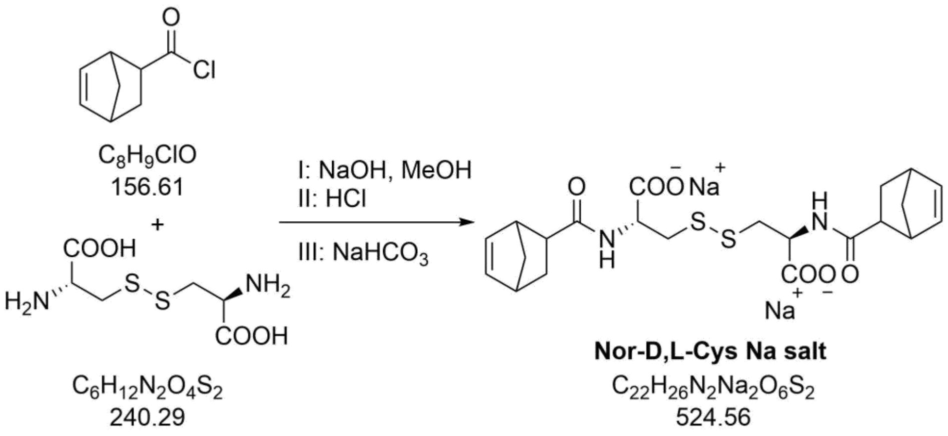

Nor-D,L-Cys was synthesized according to the general procedure from *D,L*-cystine (1000 mg, 4.2 mmol, 1 eq, CAS: 923-32-0), **NorCOCl** (1304 mg, 8.3 mmol, 2 eq) and pulverized NaOH (666 mg, 16.6 mmol, 4 eq) in dry MeOH (52 mL). After a reaction time of 20 h, MeOH was evaporated and orange crystals were obtained. The solid was washed with DCM (3 x 20 mL) to remove unreacted NorCOOMe. Thereafter the solid was dissolved in deionized water (50 mL) and acidified using HCl (1 M) causing precipitation of a sticky orange solid. The solid was dissolved in ethyl acetate and the aqueous phase was extracted with ethyl acetate (4 x 50 mL). The combined organic layers were washed with brine (50 mL) and dried with Na_2SO_4 before the solvent was removed under reduced pressure. After drying in vacuum 1.093 g (55%) of **Nor-D,L-Cys** were received as a beige foam.

For conversion into the Na salt, **Nor-D,L-Cys** (240 mg, 0.50 mmol, 1 eq) was dissolved in MeOH (2 mL) and aqueous $NaHCO_3$ solution (1 M, 1.00 mL, 2 eq) was added. The mixture was stirred for 15 min and then dried in vacuum yielding 234 mg (89%) of a beige powder.

Nor-D,L-Cys:

^{1}H-NMR (400 MHz, DMSO-d$_6$): δ = 12.78 (bs, 2H, COO<u>H</u>), 8.26-7.97 (m, 2H, N<u>H</u>), 6.13-5.82 (m, 4H, <u>H</u>C=C<u>H</u>), 4.48-4.39 (m, 2H, C<u>H</u>-NH), 3.19 (m, 2H, =CH-C<u>H</u>-CH-CON), 3.14-3.06 (m, 2H, =CH-C<u>H</u>), 2.99-2.90 (m, 2H, aliphatic), 2.84-2.81 (m, 4H, aliphatic), 2.14-1.57 (m, 3H, aliphatic), 1.33-1.22 (m, 5H, aliphatic) ppm.

HRMS (ESI): $[M + H]^+$ m/z calcd. for $C_{22}H_{29}N_2O_6S_2^+$ 481.1462; found 481.1465.

Nor-L-Cys Na salt:

^{1}H-NMR (400 MHz, D_2O): δ = 6.27-6.00 (m, 4H, H̲C=CH̲), 4.51-4.43 (m, 2H, CH̲-NH), 3.30-2.95 (m, 9H, aliphatic), 2.40-1.25 (m, 9H, aliphatic) ppm.

Synthesis of disodium *N,N'*-bis(bicyclo[2.2.1]hept-5-ene-2-ylcarbonyl)-L-homocystine, Nor-L-HCys Na salt

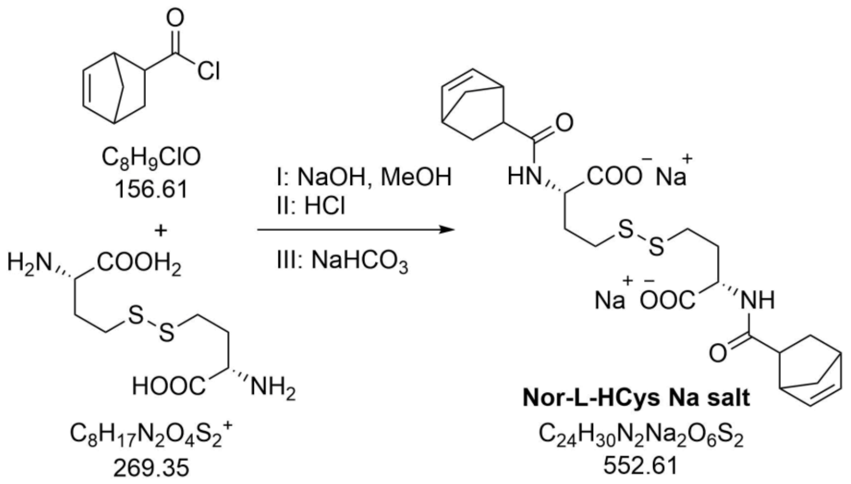

Nor-L-HCys was prepared according to the general procedure from *L*-homocystine (197 mg, 0.7 mmol, 1 eq, CAS: 626-72-2), powdered NaOH (117 mg, 2.9 mmol, 4 eq) and **NorCOCl** (229 mg, 1.5 mmol, 2 eq) in dry MeOH (14 mL). During addition of **NorCOCl** a whitish turbid suspension formed. The ice bath was removed after 40 min. and the reaction mixture was stirred for 22 h at room temperature. MeOH was removed under reduced pressure. The obtained residue was dissolved in deionized H_2O (100 mL) and washed with DCM (2 x 50 mL). The aqueous layer was acidified with HCl (1 M, 7.5 mL) causing precipitation of a white solid. The aqueous phase was extracted with ethyl acetate (4 x 50 mL). The organic phase was washed with brine (50 mL) and dried with Na_2SO_4. Solvents were removed in vacuum and the residue was dissolved in MeOH and filtered through a nylon syringe. After drying in high vacuum, **Nor-L-HCys** (225 mg, 60%) was obtained as a white foam. For conversion into the Na salt, **Nor-L-HCys** (222 mg) was dissolved in MeOH (3 mL) and aqueous $NaHCO_3$ solution (1 M, 873 µL, 2 eq) was added. It was stirred for 10 min before the solvents were removed in vacuum yielding a white foam (243 mg, quant.).

Nor-L-HCys:

^{1}H-NMR (400 MHz, DMSO-d_6): δ = 12.56 (bs, 2H, COOH̲), 8.17-7.85 (m, 2H, NH̲), 6.14-5.76 (m, 4H, H̲C=CH̲), 4.29-4.23 (m, 2H, CH̲-NH), 3.19 (m, 2H, =CH-CH̲-CH-CO), 2.83-2.67 (m, 8H, aliphatic), 2.11-

2.07 (m, 2H, aliphatic), 1.99-1.92 (m, 2H, aliphatic), 1.79-1.68 (m, 2H, aliphatic), 1.59-1.15 (m, 6H, aliphatic) ppm.

HRMS (ESI): $[M + H]^+$ m/z calcd. for $C_{24}H_{33}N_2O_6S_2^+$ 509.1775; found 509.1779.

Nor-L-HCys Na salt:

^{1}H-NMR (400 MHz, D_2O): δ = 6.30-5.96 (m, 4H, $\underline{H}C{=}C\underline{H}$), 4.35-4.26 (m, 2H, C$\underline{H}$-NH), 3.21 (m, 2H, =CH-C$\underline{H}$-CH-CO), 3.08-2.94 (m, 4H, aliphatic), 2.84-2.68 (m, 4H, aliphatic), 2.29-2.15 (m, 2H, aliphatic), 2.12-1.71 (m, 4H, aliphatic), 1.55-1.23 (m, 6H, aliphatic).

Synthesis of disodium *N,N'*-bis(bicyclo[2.2.1]hept-5-ene-2-ylcarbonyl)-D,L-homocystine, Nor-D,L-HCys Na salt

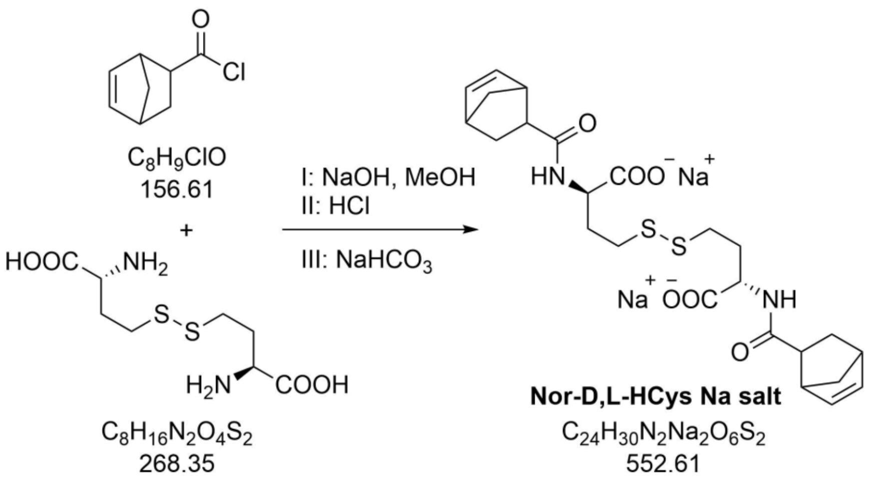

Nor-D,L-HCys was prepared according to the general procedure from *D,L*-homocystine (5.99 g, 22 mmol, 1 eq, CAS: 870-93-9), NaOH (3.58 g, 90 mmol, 4 eq) and NorCOCl (7.01 g, 45 mmol, 2 eq) in dry MeOH (460 mL). The reaction mixture was stirred for 17 h at room temperature. Due to the large-scale synthesis, the work-up differs from the general procedure. First, MeOH was removed under reduced pressure. The obtained solid was then washed with DEE (3 x 50 mL), dissolved in H_2O and acidified with HCl (1 M) causing precipitation. The precipitate was dissolved in MeOH and filtered to remove NaCl. The aqueous phase was extracted with DEE. The organic phase was washed with brine and dried over Na_2SO_4. After evaporation of solvents, the obtained white solids were united and, washed with DCM to remove remaining NorCOOMe. After drying in vacuum, 6.68 g (59%) of **Nor-D,L-HCys** were obtained as a white, highly viscous liquid. 3.00 g (5.9 mmol, 1 eq) of **Nor-D,L-HCys** were converted into the Na salt by dissolving in 400 mL MeOH at 40°C and adding to $NaHCO_3$ solution (1 M, 11.8 mL, 2 eq) yielding 3.27 g (quant.) of a white powder after drying in high vaccum.

Nor-D,L-HCys:

^{1}H-NMR (400 MHz, DMSO-d$_6$): δ = 12.56 (bs, 2H, COOH), 8.17-7.84 (m, 2H, NH), 6.12-5.75 (m, 4H, HC=CH), 4.23 (m, 2H, CH-NH), 3.20 (m, 2H, =CH-CH-CH-CO), 2.82-2.72 (m, 8H, aliphatic), 2.11-2.07 (m, 2H, aliphatic), 1.94-1.93 (m, 2H, aliphatic), 1.74-1.68 (m, 2H, aliphatic), 1.59-1.15 (m, 6H, aliphatic) ppm.

HRMS (ESI): [M + H]$^+$ m/z calcd. for $C_{24}H_{33}N_2O_6S_2^+$ 509.1775; found 509.1776.

Nor-D,L-HCys, Na salt:

^{1}H-NMR (400 MHz, D_2O): δ = 6.29-5.95 (m, 4H, HC=CH), 4.34-4.26 (m, 2H, CH-NH), 3.22 (m, 2H, =CH-CH-CH-CO), 3.08-2.96 (m, 4H, aliphatic), 2.77-2.66 (m, 4H, aliphatic), 2.25-2.15 (m, 2H, aliphatic), 2.12-1.71 (m, 4H, aliphatic), 1.58-1.21 (m, 6H, aliphatic).

Synthesis of disodium 1,1'-dimethyl-*N,N'*-bis(bicyclo[2.2.1]hept-5-ene-2-ylcarbonyl)-L-cystine ester, Nor-L-CysMe

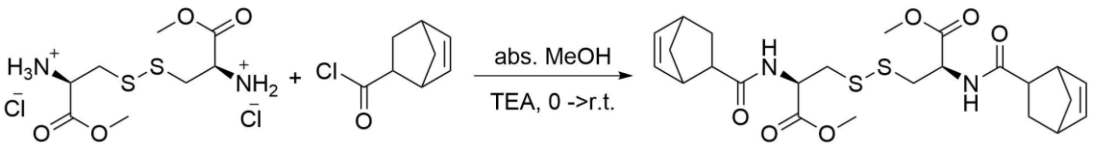

$C_8H_{17}Cl_2N_2O_4S_2^+$ 340.25

C_8H_9ClO 156.61

Nor-L-CysMe $C_{24}H_{32}N_2O_6S_2$ 508.65

The synthesis of **Nor-L-CysMe** is roughly based on a modified synthetic procedure.[6]

L-Cystine dimethyl ester dihydrochloride (1195 mg, 3.5 mmol, 1.0 eq) and triethylamine (2.0 mL, 14.7 mmol, 4.2 eq) were suspended in dry DCM (30 mL) in a round bottom flask equipped with a septum. The mixture was cooled to 0 °C and **NorCOCl** (1.1 mL, 7.7 mmol, 2.2 eq) was added dropwise using a syringe. After complete addition, the ice bath was removed and the reaction mixture was stirred at room temperature. The progress of the reaction was monitored by ^{1}H-NMR. After 5.5 h the reaction mixture was diluted with ethyl acetate (100 mL) and extracted with saturated sodium bicarbonate solution (50 mL), water (50 mL) and brine (50 mL). The organic phase was dried with Na_2SO_4 and the solvent was removed in vacuum. The raw product (solid foam) was purified by column chromatography using light pertol:ethyl acetate (50-60%) as eluent. Two fractions were received of which one contained both exo- and endo-norbornene based products (514 mg, 29%) and the other only the endo-norbornene product (1004 mg, 56%). The white liquids were dried at HV while being carefully heated with warm air.

endo-Nor-L-CysMe:

^{1}H-NMR (400 MHz, DMSO-d$_6$): δ = 8.13 (t, J = 7.4 Hz, 2H, N<u>H</u>), 6.08 (m, 2H, <u>H</u>C=CH), 5.80 (m, 2H, HC=C<u>H</u>), 4.47 (m, 2H, C<u>H</u>-NH), 3.63 (m, 6H, OC<u>H</u>$_3$), 3.19 (m, 2H, =CH-C<u>H</u>-CH-CO), 3.14-3.07 (m, 2H, =CH-C<u>H</u>), 2.97-2.91 (m, 2H, aliphatic), 2.86-2.81 (m, 4H, aliphatic), 1.74-1.69 (m, 2H, aliphatic), 1.32-1.24 (m, 6H, aliphatic) ppm.

HRMS (ESI): $[M + H]^+$ m/z calcd. for $C_{24}H_{33}N_2O_6S_2^+$ 509.1775; found 509.1775.

Synthesis of 3,3'-(((disulfanediylbis(ethane-2,1-diyl))bis(oxy))bis(carbonyl))-bis-(bicyclo[2.2.1]hept-5-ene-2-carboxylic acid), Nor-O-SS

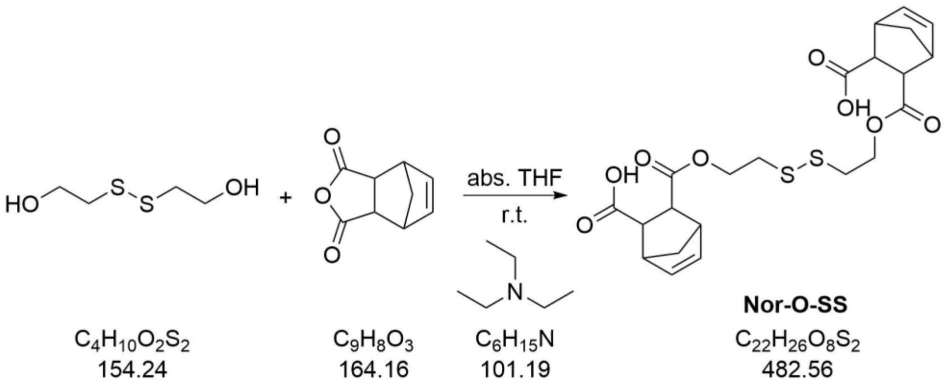

The synthesis of **Nor-O-SS** is roughly based on a modified synthetic procedure.[3]

2-Hydroxyethyl disulfide (573 mg, 3.7 mmol, 1 eq) was dissolved in dry THF (5 mL) in Ar atmosphere. A solution of carbic anhydride (1220 mg, 7.4 mmol, 2 eq) in dry THF (15 mL) was added to the stirred solution. Thereafter, trimethylamine (1.55 mL, 11.1 mmol, 3 eq) was added slowly and the reaction was stirred at room temperature overnight. After 14 h ^{1}H-NMR showed 80% consumption of starting materials. Hence, the reaction mixture was refluxed for 3 h to increase the conversion. Then the reaction mixture was dried in vacuum before it was dissolved in sat. aq. $NaHCO_3$ solution (50 mL) to give a “clear” solution. The product was precipitated using conc. HCl giving a sticky white solid. Ethyl acetate (100 mL) was added to dissolve the product. After phase separation the aqueous layer was extracted with more ethyl acetate (3x 50 mL) and the combined organic layers were washed with brine, dried over Na_2SO_4 and the solvent was removed in vacuum yielding a whitish oil (1.907 g, 106%). The raw product contained traces of solvent and both starting materials. Hence, the raw product was purified by column chromatography (110 g silica) using light petrol:ethyl acetate (1% glacial acetic acid) = 20%-55% as eluent. After drying in high vacuum while heating to 50 °C a colourless liquid was received still containing starting material due to the decomposition of **Nor-O-SS** (1.678 g, 94%).

A further attempt of purification included dissolution of the product in diethyl ether (10 mL) and addition of light petrol (80 mL) to precipitate the product as white sticky solid. After centrifugation at 5000 rpm (6 °C, 20 min), the supernatant was decanted off and the white precipitate was dissolved in DCM and transferred to a round bottom flask. After drying in vacuum 1.274 g (71%) of a viscose white liquid were received, still containing carbic anhydride and 2-hydroxyethyl disulfide.

^{1}H-NMR (400 MHz, DMSO-d$_6$): δ = 11.94 (s, 2H, -COOH), 6.19-6.17 (dd, J = 3.0 Hz, 2.9 Hz, 2H, CH=CH), 6.07-6.05 (dd, J = 3.0 Hz, 2.9 Hz, 2H, CH=CH), 4.21-4.06 (m, 4H, CH$_2$-CH$_2$S), 3.32-3.23 (m, 4H, aliphatic), 3.05-3.02 (m, 4H, aliphatic), 2.91-2.88 (m, 4H, S-CH$_2$), 1.36-1.26 (m, 4H, CH-CH$_2$-CH) ppm.

^{13}C-NMR (100 MHz, DMSO-d$_6$): δ = 173.1 (s, COOH), 171.9 (s, COOCH$_2$), 135.2 (d, CH=CH), 134.3 (d, CH=CH), 61.5 (t, COOCH$_2$), 48.0 (t, CH-CH$_2$-CH), 47.9 (d, CHCOOCH$_2$), 47.1 (d, CHCOOH), 46.0 (d, CHCH-ester), 45.5 (d, CHCH-acid), 36.3 (t, S-CH$_2$) ppm.

HRMS (ESI): $[M + H]^+$ m/z calcd. for $C_{22}H_{27}O_8S_2^+$ 483.1142; found 481.1145.

Synthesis of sodium 3,3'-(((disulfanediylbis(ethane-2,1-diyl))bis(oxy))-bis(carbonyl))-bis(bicyclo[2.2.1]hept-5-ene-2-carboxylate), Nor-O-SS Na salt

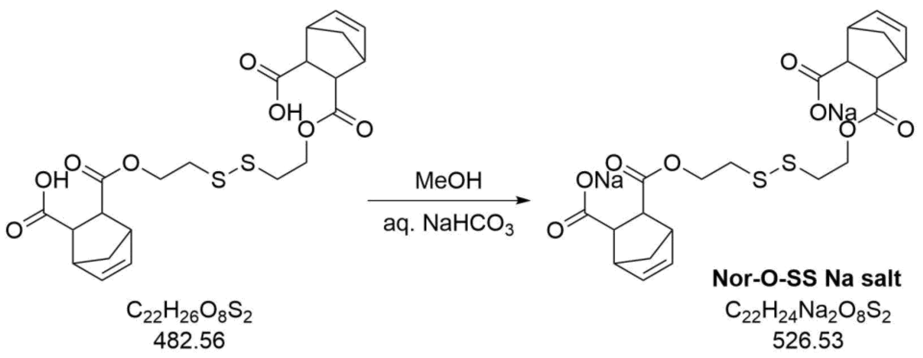

Nor-O-SS (117 mg, 0.24 mmol, 1 eq) was dissolved in dry MeOH (1 mL) and added dropwise to a stirred aqueous solution of $NaHCO_3$ (0.1 M, 4.86 mL, 0.49 mmol, 2 eq). Formation of gas and a solid film which rapidly dissolved again was observed. After complete addition, MeOH (1 mL) was used to transfer remaining **Nor-O-SS** to the aqueous solution. The solution was stirred for 15 min and then dried in high vacuum yielding a transparent glass with crystalline sites (127 mg, quant.). The product still contained decomposition products.

^{1}H-NMR (400 MHz, D$_2$O): δ = 6.31-6.29 (dd, J = 2.9 Hz, 2.7 Hz, 2H, CH=CH), 6.20-6.18 (dd, J = 2.9 Hz, 2.7 Hz, 2H, CH=CH), 4.41-4.35 (m, 2H, O-CHH-CH$_2$), 4.25-4.19 (m, 2H, O-CHH-CH$_2$), 3.36-3.25 (m, 4H, aliphatic), 3.11 (m, 4H, aliphatic), 3.05-2.98 (m, 4H, S-CH$_2$), 1.43-1.36 (m, 4H, CH-CH$_2$-CH) ppm.

EXPERIMENTAL PROCEDURES

Linker reactivity estimation by ^{1}H-NMR in D_2O.

For reactivity testing, linkers were reacted with 2-mercaptoethanol at a functional group ratio of 1:1 in D_2O by irradiation at 400-500 nm (20 mW cm^{-2}) in presence of 0.6 mM LiTPO. Stock solutions of the respective linker (150 mM), 2-mercaptoethanol (300 mM) and photoinitiator (6 mM) were prepared. In a typical experiment, to 420 µL of solvent, 60 µL of each stock solution were added to give final concentrations of 15 mM linker, 30 mM 2-mercaptoethanol and 0.6 mM photoinitiator (2 mol% of ene) in a total volume of 600 µL. The samples were prepared in GC-MS vials, vortexed and then irradiated on the measuring platform of a photorheometer for various irradiation periods. The photorheometer (MCR-302 WESP, Anton-Paar GmbH) was coupled to an OmniCure®S2000 Spot UV Light Curing System (Excelitas Technologies) from the underside of the glass plate via a waveguide. The total irradiation intensity was measured through the glass bottom of a GC-MS vial at the measuring platform using an Ocean Optics USB 2000+ (Ocean Insight Inc.) spectrometer. After irradiation, the sample solutions were transferred to brown NMR-tubes and measured by ^{1}H-NMR. For estimation of norbornene-consumption, the integral of the norbornene protons (HC=CH, 4H at t = 0 s) was monitored. In experiments conducted in D_2O, the signal of the proton at the α-carbon (CH-NH, 2H) served as internal reference for calibration, whereas in DMSO-d_6 the amide-proton (NH, 2H) was used.

Linker reactivity estimation by ^{1}H-NMR in DMSO-d_6.

Experiments were conducted similar to the measurements in D_2O. Here, linkers were reacted with 2-mercaptoethanol at a functional group ratio of 1:1 in DMSO-d_6 by irradiation at 400-500 nm (20 mW cm^{-2}) in presence of TPO-L (ethyl (2,4,6-trimethylbenzoyl)phenylphosphinate).

Photorheology.

Light-induced hydrogel formation was investigated with selected linkers of the series by means of photorheology using a MCR-302 WESP rheometer equipped with a P-PTD 200/GL peltier glass plate, a H-PTD 200 peltier-temperature-controlled hood, and a D-CP/PP 7 measuring tool (all Anton-Paar GmbH). Hydrogel samples were prepared by reacting linkers with 10 wt% of thiol-terminated eight-armed PEG (8armPEG20k-SH, Mw ~20 kDa) using LiTPO as photoinitiator. For mixing, a vortex and a micro-centrifuge were used. Samples were prepared in 500 µL safe-lock tubes. The rheological analysis was performed with a plate-plate geometry (PP08 disposable, tool diameter = 8 mm, gap size = 0.8 mm) at 20 °C. After mixing of the components, 45 µl of the sample solution were pipetted into the gap (1 mm) between measuring tool and glass plate utilizing capillary force, before the tool was lowered into the measuring position at a gap of 0.8 mm. The formulations were sheered with a strain of 1% and a

frequency of 1 Hz. After 1 min of equilibration, photocuring of the hydrogel samples was induced by UV-irradiation projected via a waveguide from the underside of the glass plate using an OmniCure® LX400 LED UV Spot Curing System with a 385 nm LED head (Excelitas Technologies) with a specific power of ~6 mW cm^{-2} at the measuring platform.[7] All measurements were performed at least in triplicates, except for experiments in which a high amount of photoinitiator was used or the thermal or mechanical stability of the hydrogel was investigated. Error bars are illustrating the standard deviation in graphs. The formed hydrogels disks were utilized for subsequent swelling tests in PBS.

Hydrogel swelling experiments.

For swelling experiments, the hydrogel disks, produced in the course of photorheology measurements, were carefully removed from the photorheometer measuring platform and placed in individual tared petri dishes. After initial weighing of the samples, they were swollen in PBS (~2 mL). For weighing, the liquid was pipetted off, and the hydrogel disks were carefully patted dry with paper towel. Thereafter, the disks were submersed in fresh PBS again. All swelling experiments were performed in triplicates.

Optimization of the photoinitiator concentration

Stock solutions of 8armPEG20k-SH, linker **Nor-D,L-HCys** (190 mM) and LiTPO (18 mM) were prepared in PBS. The stock solutions were combined to give sample solutions with final concentrations of 10 wt% 8armPEG20k-SH, 22 mM Nor-D,L-HCys (SH:ene = 1:1) and a variable LiTPO concentration (0.2 mM, 0.4 mM, 0.6 mM, 15 mM). For high LiTPO concentrations (15 mM), 8armPEG20k-SH was directly dissolved in the LiTPO stock solution. Aliquots of 45 µl of the sample solutions were measured on the photorheometer at the settings described in section 6.3.2.0. After 1 min of equilibration time, the prepolymer formulations were exposed to 385 nm light (~6 mW cm^{-2}) for 5 min. The formulation containing 15 mM LiTPO was also polymerized with shuttered light (60 s off-time; 6 cycles of 5 s on-time and 15 s off-time, 3 min of constant irradiation).

Thermostability of 8arm-D,L-HCys 100 hydrogel

An aliquot (45 µL) of **8arm-D,L-HCys 100** prepolymer solution (containing 0.4 mM LITPO) was *in situ* photopolymerized at 20 °C on the photorheometer at the settings described in section 6.3.2.0, before the temperature was continuously increased up to 90°C at a heating rate of 1 °C/min. To prevent evaporation of water, the measuring system was sealed with a ring of paraffin.

Mechanostability of 8arm-D,L-HCys 100 hydrogel

For the amplitude sweep, after *in situ* photo-polymerization of **8arm-D,L-HCys 100** prepolymer solution, the amplitude of the applied strain was logarithmically increased from 0.1% to 1000% at a constant frequency of 1 Hz. For the dynamic strain amplitude cycling experiment, after *in situ* polymerization, a series of four strain cycles was applied varying strains between 350% and 1% in 60 s periods.

Stabilization of 8arm D,L-HCys 100 hydrogel by a non-cleavable background network

Several prepolymer solutions were prepared from stock solutions of 8armPEG20k-SH (11.4 wt%), **Nor-O-CC** (190 mM), **Nor-D,L-HCys** (190 mM) and LiTPO (18 mM) in PBS. Mixed linker stock solutions were prepared at a ratio of 1:3 (CC:SS) and 1:7 (CC:SS). The stock solutions were combined to give sample solutions with final concentrations of 10 wt% 8armPEG20k-SH, 22 mM total linker (SH:ene = 1:1) and 0.4 mM LiTPO. For the preparation of **8arm-SS-CC (3:1) 15 mM** formulation, 8armPEG20k-SH was directly dissolved in the LiTPO stock solution and combined with the 1:3 (CC:SS) linker stock at a 1:1 ratio of SH:ene.

Stabilization of 8arm D,L-HCys 100 hydrogel by capping of free thiols by maleimide

After in situ polymerization of **8arm-D,L-HCys 100** hydrogel, samples were weighted and then swollen in a solution of maleimide (4 mM) in PBS for 1 h. Thereafter, the samples were further immersed in PBS and the swelling was monitored gravimetrically.

Consumption of free thiols by an excess of Nor-D,L-HCys linker

Stock solutions of linker **Nor-D,L-HCys** (190 mM), LiTPO (18 mM) were prepared in PBS. 8armPEG20k-SH was dissolved in PBS and combined with the stock solutions to give sample solutions with final concentrations of 10 wt% 8armPEG20k-SH, 0.4 mM LiTPO and a variable concentration of **Nor-D,L-HCys** depending on the SH:ene ratio [1:1 = 22.2 mM (**8arm-D,L-HCys 100**), 1:1.15 = 25.6 mM (**8arm-D,L-HCys 115**), 1:1.20 = 26.7 mM (**8arm-D,L-HCys 120**), 1:1.24 = 27.6 mM (**8arm-D,L-HCys 124**)].

Stabilization of 8armPEG-based hydrogels by the use of Nor-L-HCys

Stock solutions of linker **Nor-L-HCys** (190 mM) and LiTPO (18 mM) were prepared in PBS. 8armPEG20k-SH was dissolved in PBS and combined with the stock solutions to give sample solutions with final concentrations of 10 wt% 8armPEG20k-SH, 0.4 mM LiTPO and a variable concentration of Nor-L-HCys depending on the SH:ene ratio [1:1 = 22.2 mM (100), 1:1.20 = 26.7 mM (120)].

Two-photon micropatterning.

Prepolymer formulations (25 µl) of **8arm-D,L-HCys 120** hydrogel and **8arm-L-HCys 100** hydrogel were polymerized in cylindrical silicon molds (Ø = 6 mm, d = 0.8 mm) between methacrylate glass-bottom µ-dishes (35 mm, Ibidi GmbH) and cover glass by irradiation in a Boekel Scientific UV Crosslinker AH (365 nm, 2 J). Subsequently, the molds were removed and the solidified hydrogel disks were swollen in PBS for 3 d to reach equilibrium swelling. Thereafter, the hydrogels were submersed in solutions of DAS in PBS at different concentrations (0.5 mM, 1.0 mM, 2.0 mM) for at least 5 h to allow diffusion of the chromophore into the network. Then samples were cut using a scalpel to generate a sharp edge. Micropatterning was performed by means of two-photon degradation. Details of the experimental setup have been reported previously.[8] Briefly, the setup is based on a femtosecond laser (MaiTai DeepSee, Spectra Physics) operating at 720 nm, with a pulse length of 70 fs after the objective (C-Achroplan 32x/0.85 W, ZEISS). Parallel micro-channels with quadratic cross sections (l = 300 µm, A = 20 µm x 20 µm) were eroded from the edge into the bulk of the hydrogel at different laser powers ranging from 10–120 mW and scanning along the channel (x-axis). The scanning speed was 200 mm/s (hatch distance: 0.1 µm, z-layer distance: 0.5 µm). Individual x,y-planes were either scanned once or twice. After micropatterning, the samples were washed with PBS twice and then soaked in a solution of high molecular weight FITC-dextran (**FITC2000**, 1 mg mL^{-1}, Mw ~2,000 kDa) in PBS at room temperature for more than 4 h. Channels were visualized by laser scanning microscopy (LSM 700, ZEISS). If no channels were visible, the hydrogels were also imaged after swelling in lower molecular weight FITC-dextran (**FITC500**, 1 mg mL^{-1}, Mw ~500 kDa) for at least 5 h.

SUPPORTING FIGURES

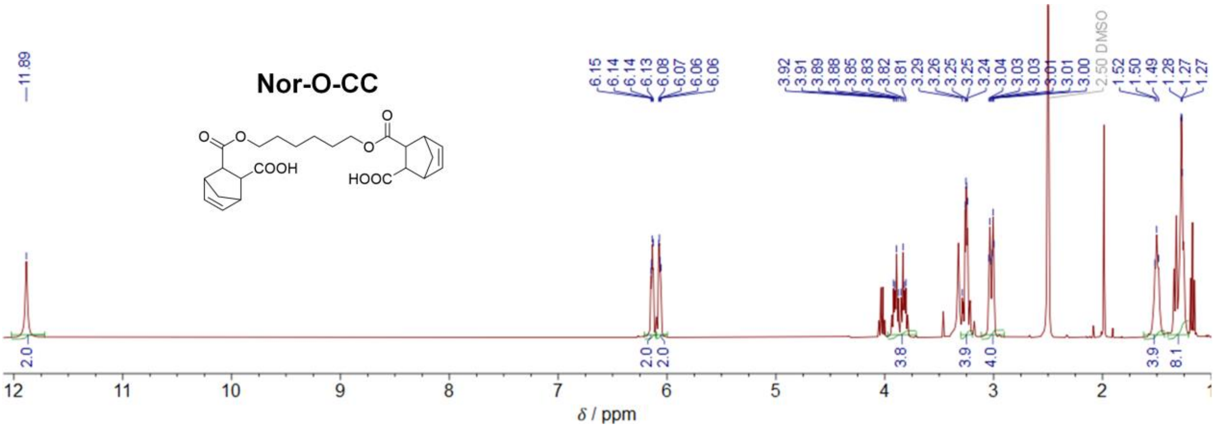

Figure S1. ^{1}H-NMR (400 MHz, DMSO-d_6) of **Nor-O-CC**.

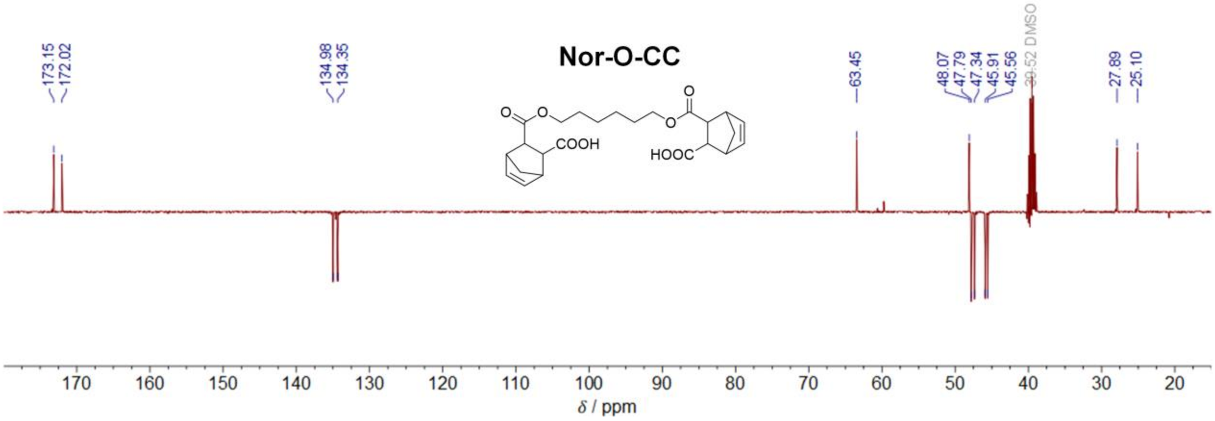

Figure S2. ^{13}C-NMR (100 MHz, DMSO-d_6) of **Nor-O-CC**.

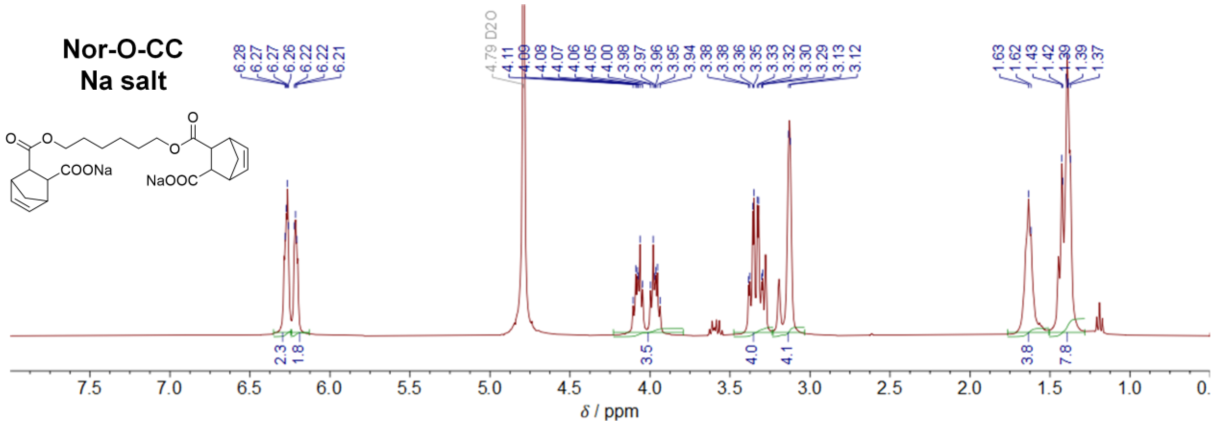

Figure S3. ^{1}H-NMR (400 MHz, D_2O) of **Nor-O-CC** Na salt.

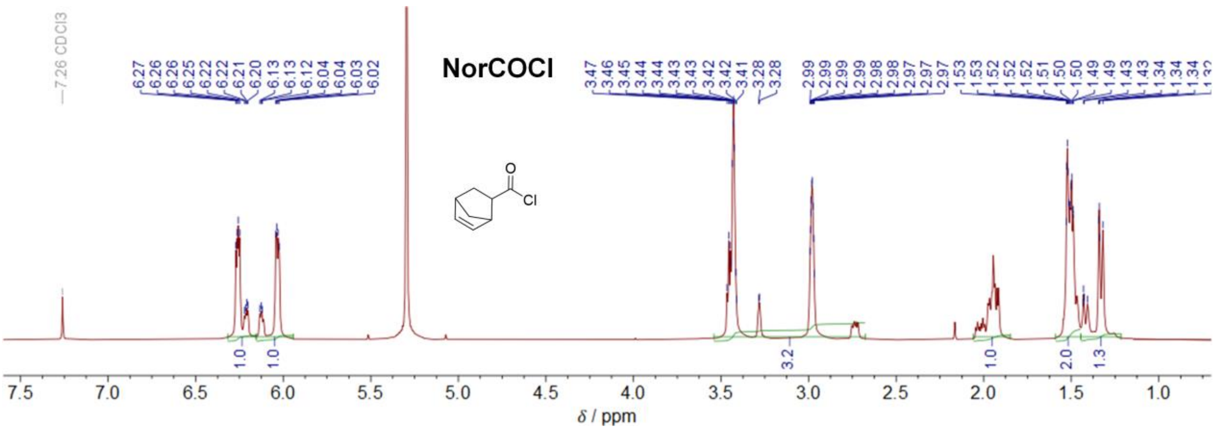

Figure S4. ^{1}H-NMR (400 MHz, $CDCl_3$) of **NorCOCl**.

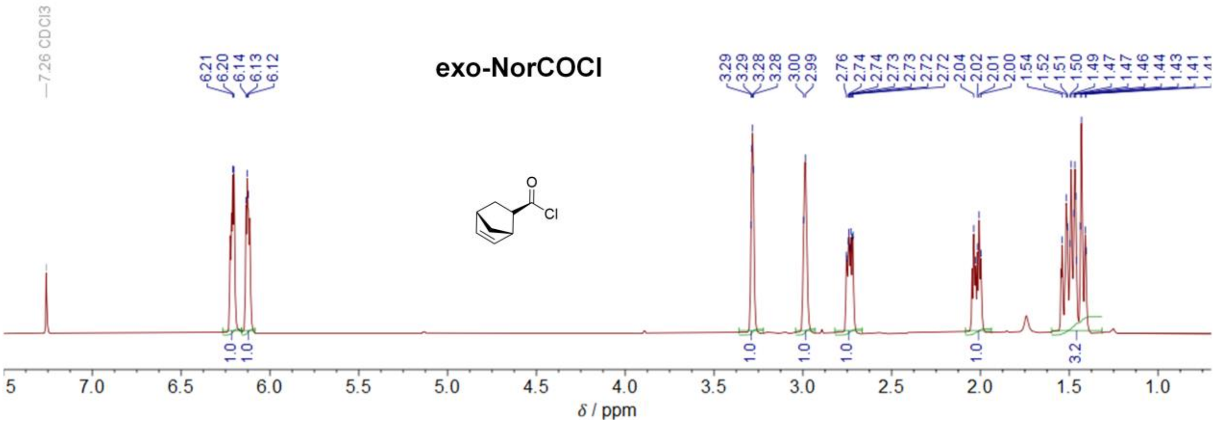

Figure S5. ^{1}H-NMR (400 MHz, $CDCl_3$) of **exo-NorCOCl**.

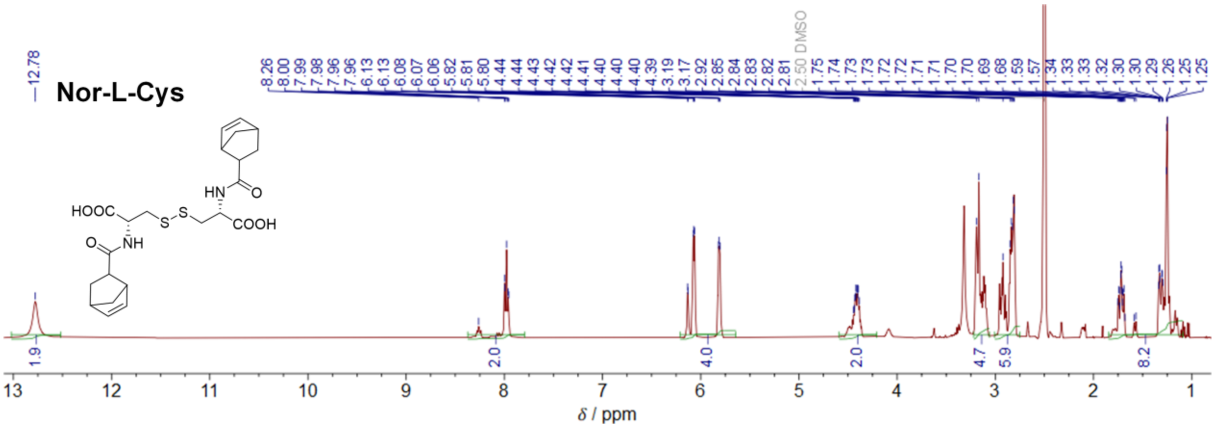

Figure S6. ^{1}H-NMR (400 MHz, DMSO-d_6) of **Nor-L-Cys**.

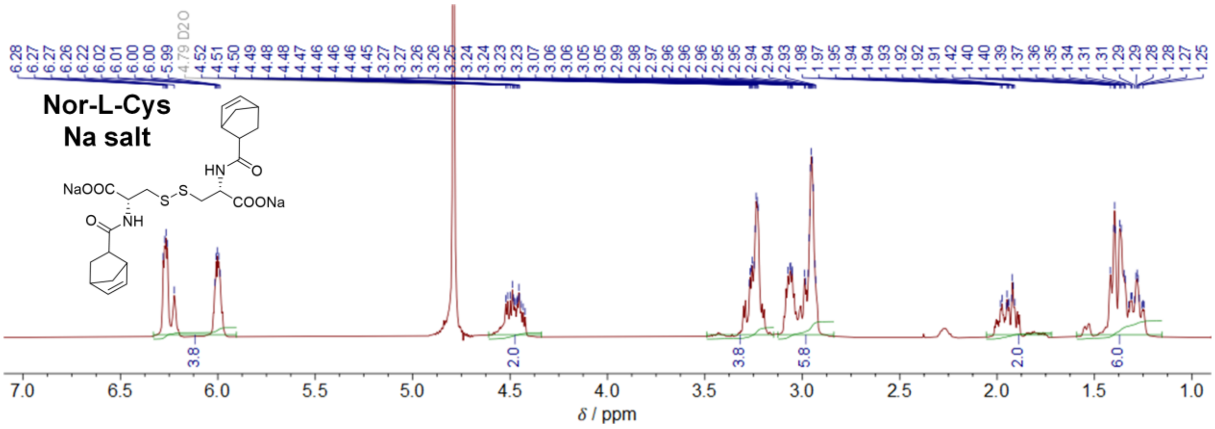

Figure S7. ^{1}H-NMR (400 MHz, D_2O) of **Nor-L-Cys** Na salt.

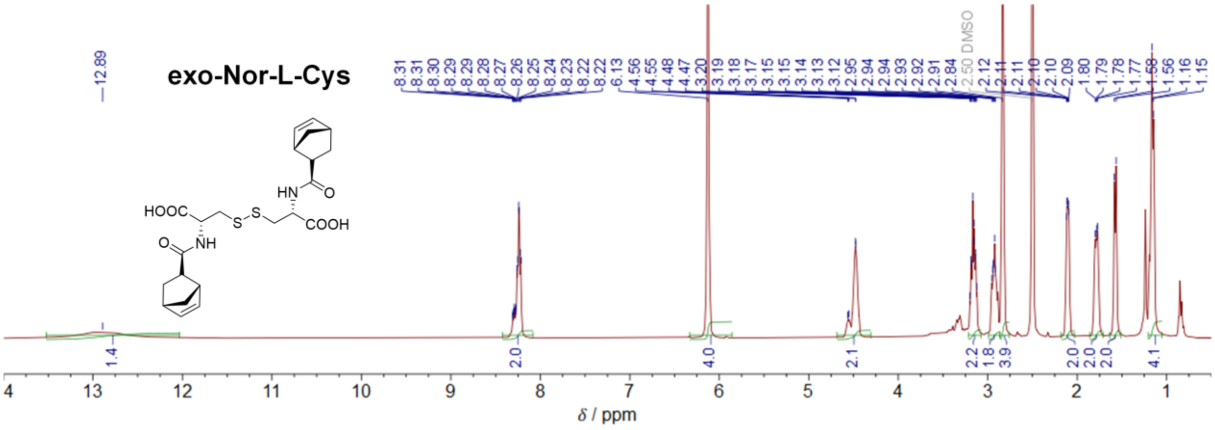

Figure S8. ^{1}H-NMR (400 MHz, DMSO-d_6) of **exo-Nor-L-Cys**.

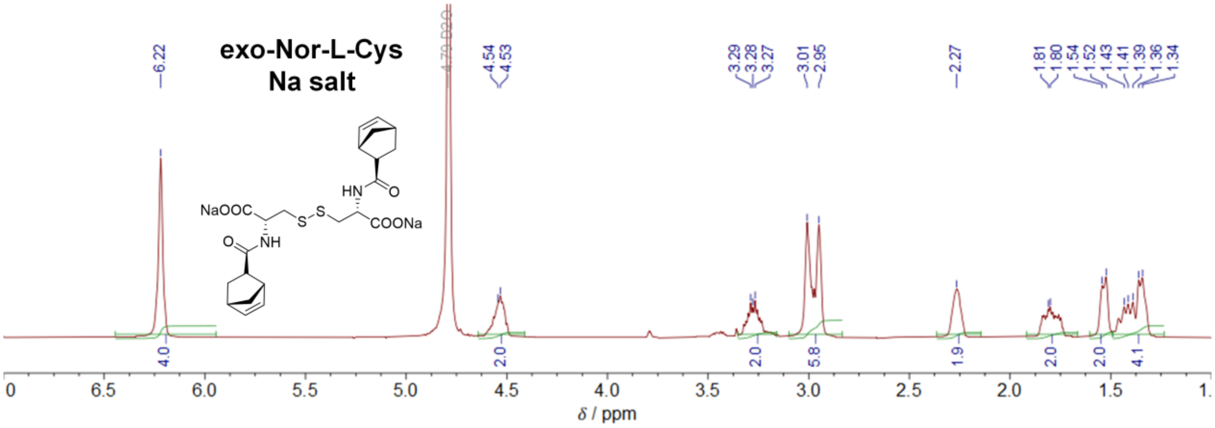

Figure S9. ^{1}H-NMR (400 MHz, D_2O) of **exo-Nor-L-Cys** Na salt.

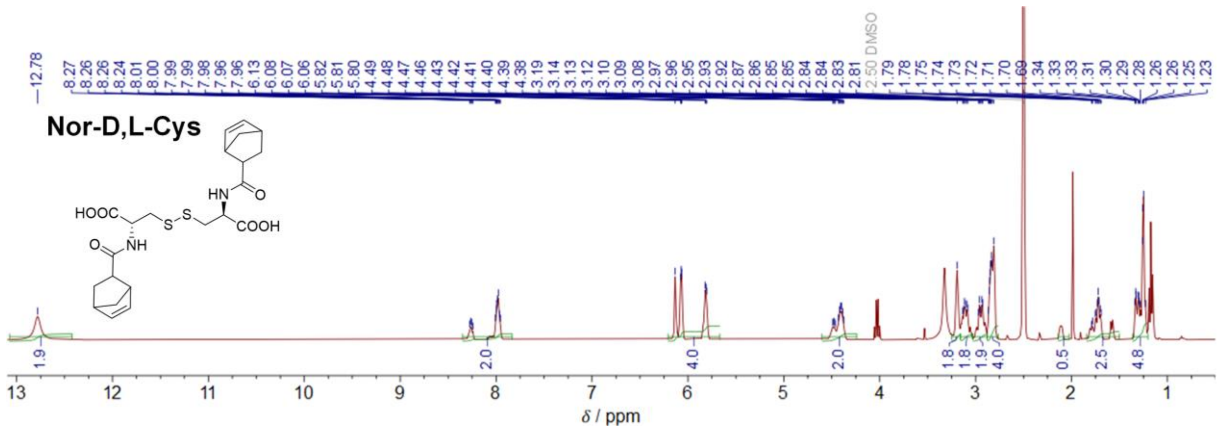

Figure S10. ^{1}H-NMR (400 MHz, DMSO-d$_6$) of **Nor-D,L-Cys**.

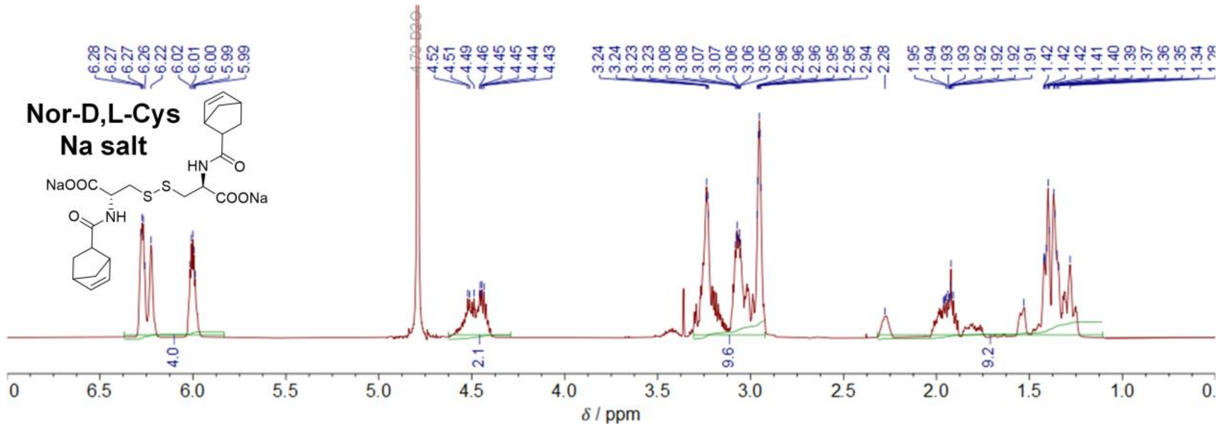

Figure S11. ^{1}H-NMR (400 MHz, D_2O) of **Nor-D,L-Cys** Na salt.

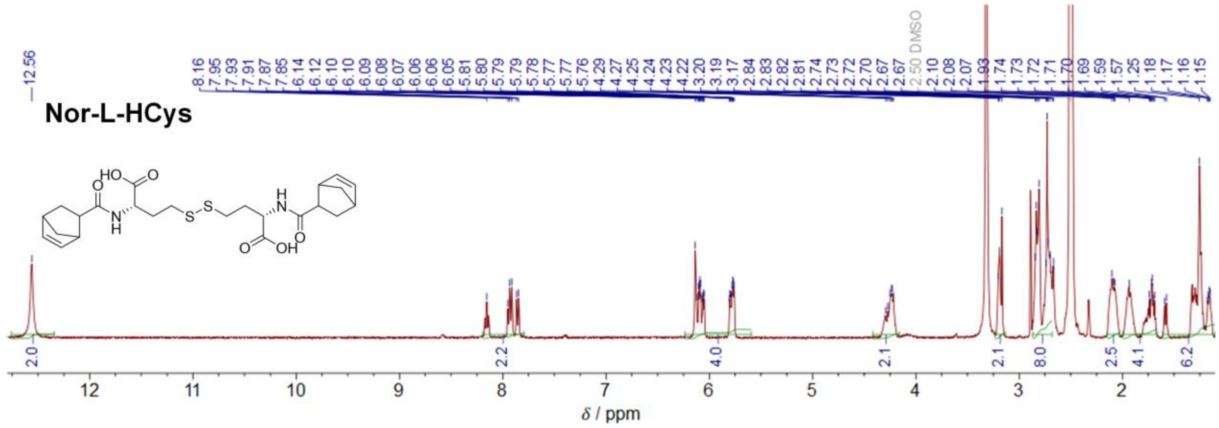

Figure S12. ^{1}H-NMR (400 MHz, DMSO-d$_6$) of **Nor-L-HCys**.

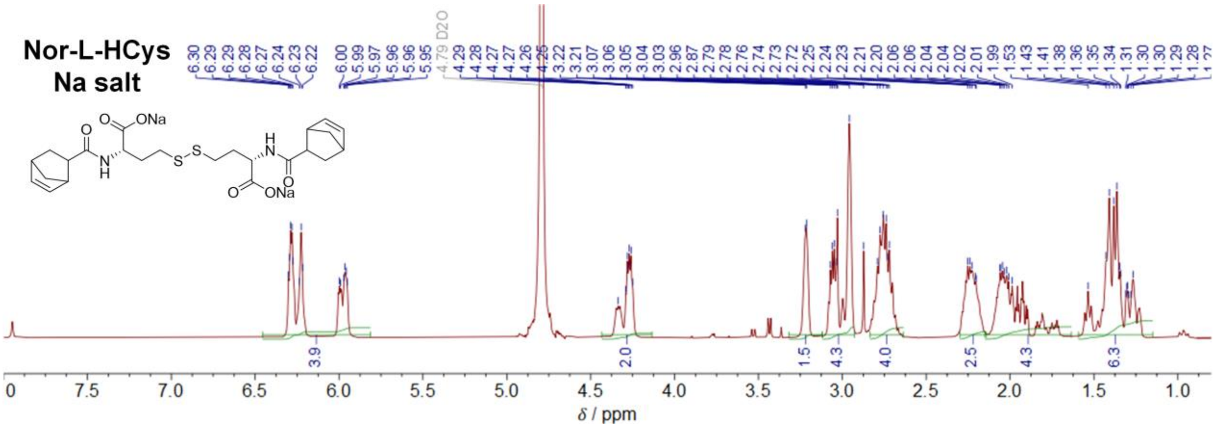

Figure S13. ^{1}H-NMR (400 MHz, D_2O) of **Nor-L-HCys** Na salt.

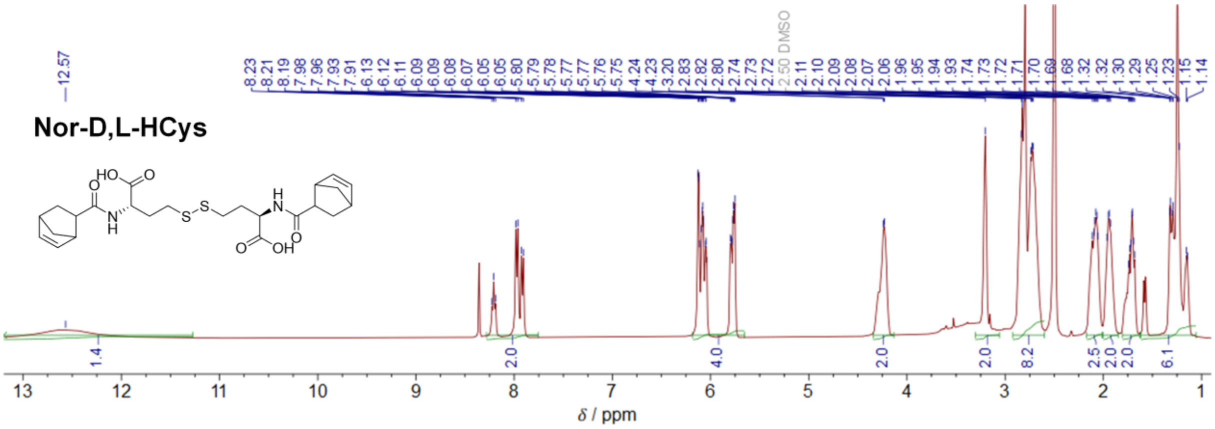

Figure S14. ^{1}H-NMR (400 MHz, DMSO-d_6) of **Nor-D,L-HCys**.

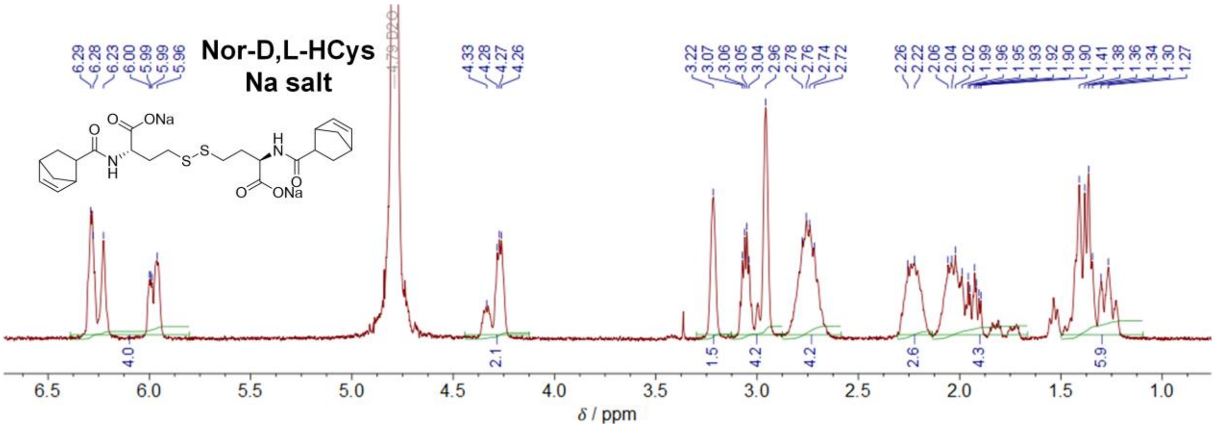

Figure S15. ^{1}H-NMR (400 MHz, D_2O) of **Nor-D,L-HCys** Na salt.

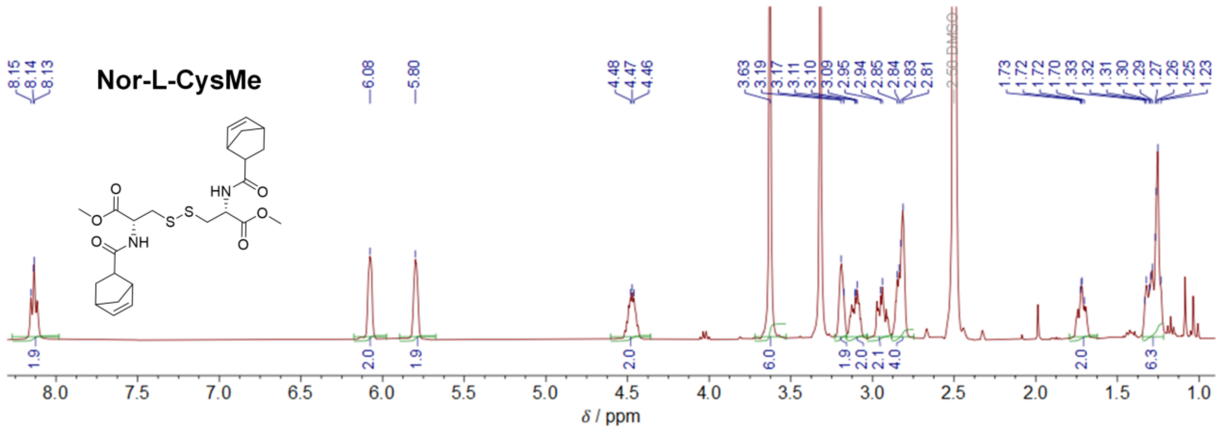

Figure S16. ^{1}H-NMR (400 MHz, DMSO-d_6) of **Nor-L-CysMe**.

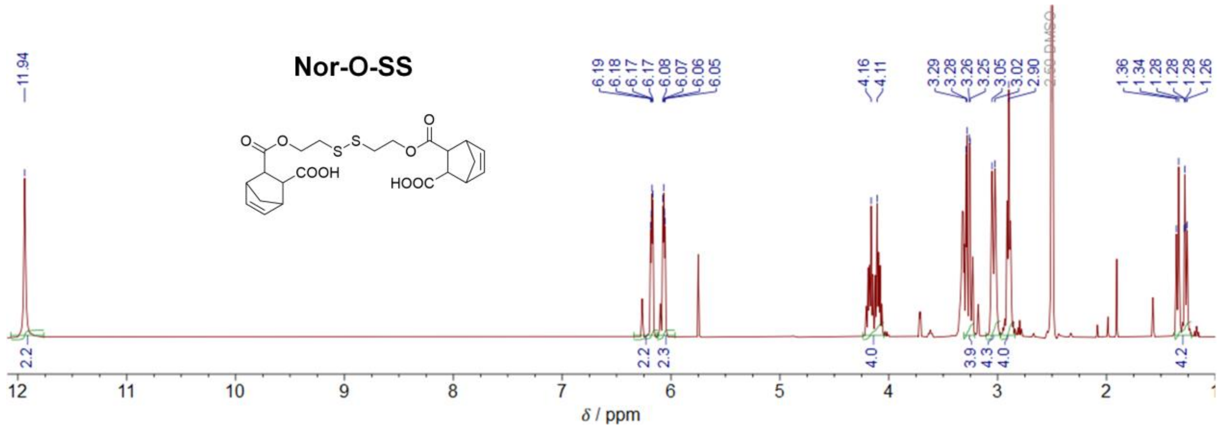

Figure S17. ^{1}H-NMR (400 MHz, DMSO-d_6) of **Nor-O-SS**.

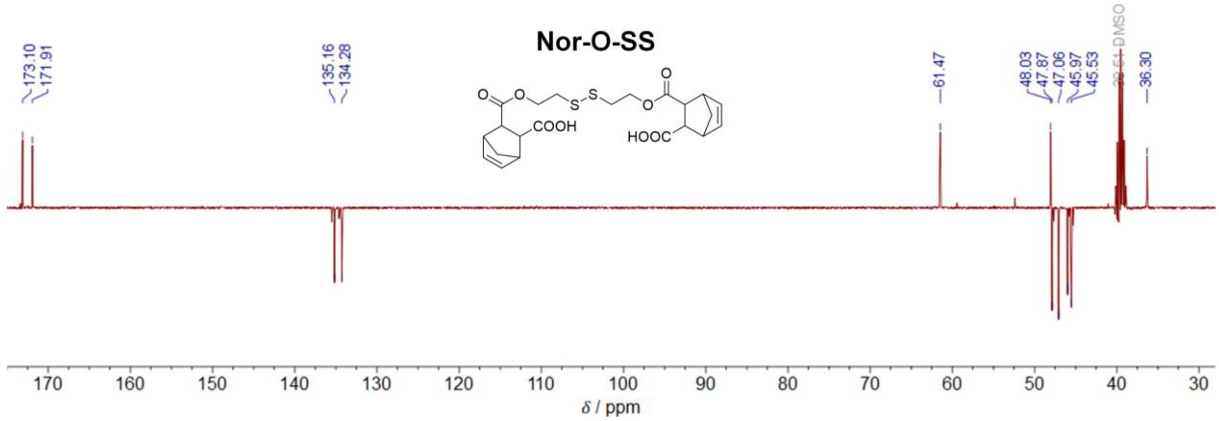

Figure S18. ^{13}C-NMR (100 MHz, DMSO-d_6) of **Nor-O-SS**.

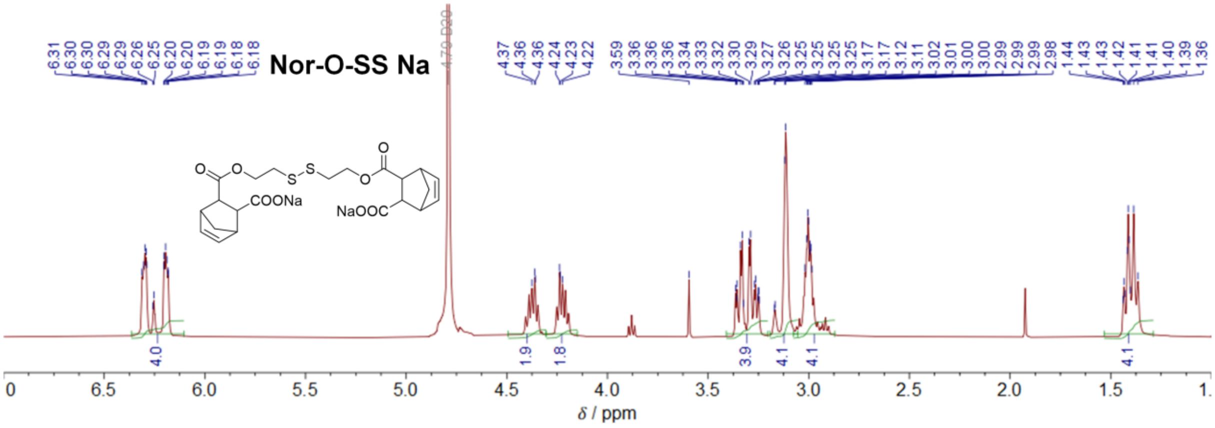

Figure S19. ^{1}H-NMR (400 MHz, D_2O) of **Nor-O-SS** Na salt.

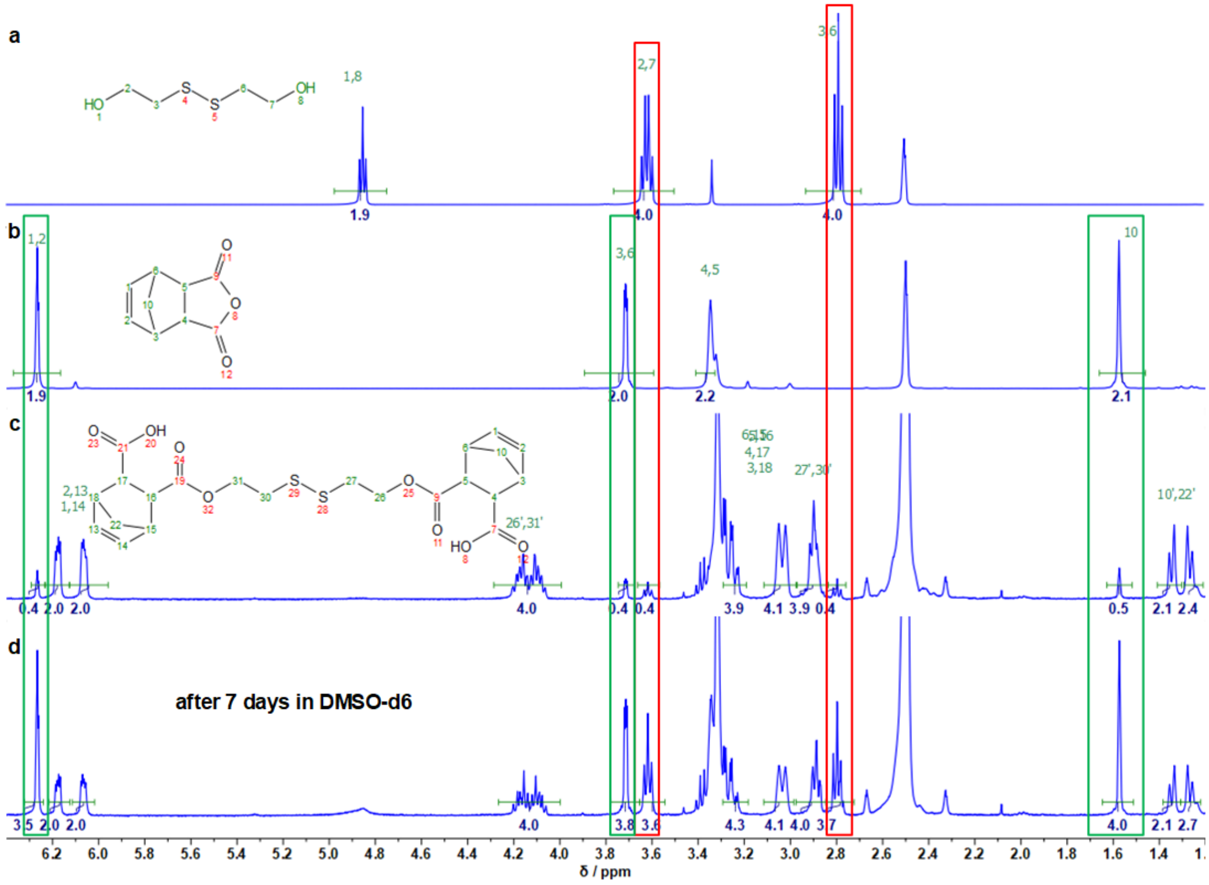

Figure S20. Self-immolation of **Nor-O-SS**. Comparison of ^{1}H-NMR spectra of starting materials (a) hydroxyethyl disulfide, (b) carbic anhydride and product (c) **Nor-O-SS** after column chromatography and (d) after 7 days in DMSO-d_6 solution. As the signals of the starting materials reappeared again, **Nor-O-SS** obviously decomposed into its starting materials via elimination of carbic anhydride. Since DMSO-d_6 is an aprotic solvent, hydrolysis can be excluded.

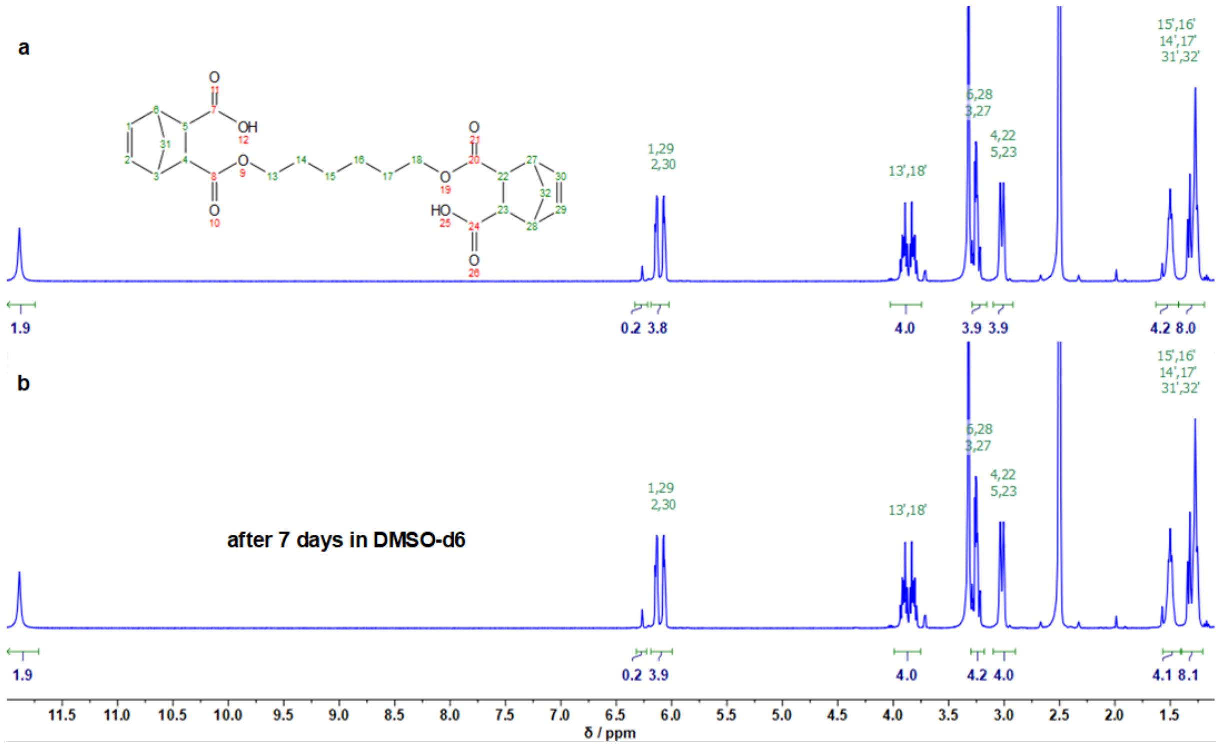

Figure S21. ^{1}H-NMR spectra of non-cleavable linker **Nor-O-CC** (a) after column chromatography and (b) after 7 days in DMSO-d_6 solution. **Nor-O-CC** proved to be stable against elimination in DMSO-d_6.

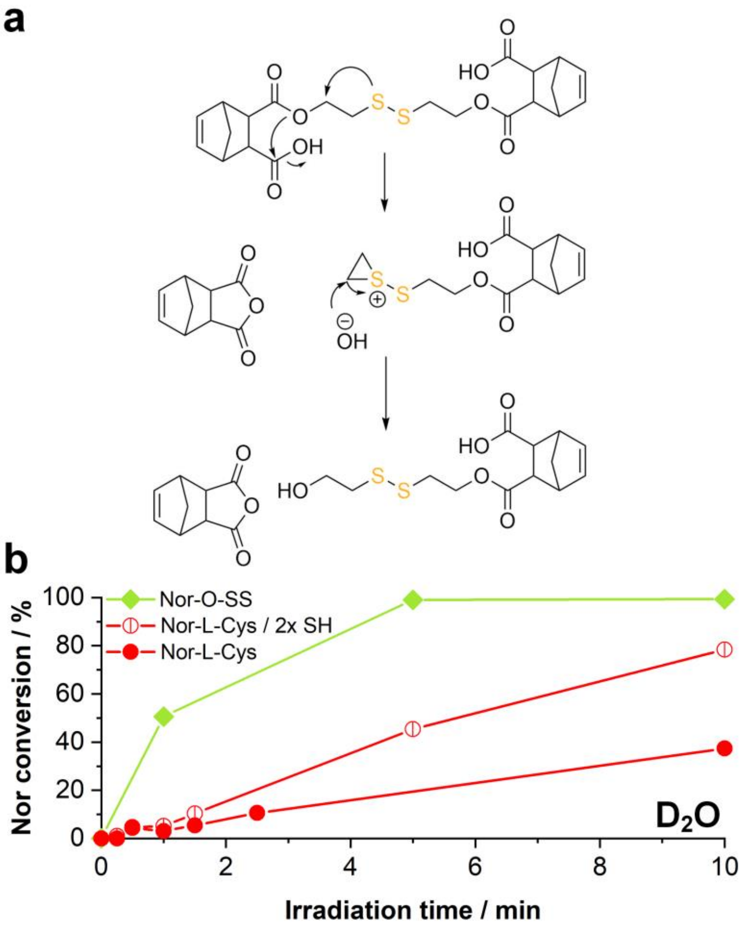

Figure S22. (a) Proposed elimination mechanism of carbic anhydride from **Nor-O-SS** triggered by the neighbouring group effect of the β-positioned disulfide via formation of an intermediate cyclic sulfonium ion. The results depicted in Figure S20 and S21 indicate that the presence of the disulfide is triggering the elimination reaction, which occurs most probably due to a neighboring group effect of the disulfide in combination with the free carboxylic acid in close proximity to the ester.[9] Thioethers in β-postion to reactive sites are known to participate in elimination reactions via the formation of an intermediate cyclic sulfonium ion.[10] In contrast, no elimination occurs in the analog disulfide-free linker **Nor-O-CC** (Figure S21). Hence, the simple disulfide-based linker **Nor-O-SS** is not expected to withstand prolonged incubations of a hydrogel made thereof. Moreover, the purity and hence the crosslinking properties could not be guaranteed. (b) A preliminary ^{1}H-NMR conversion test showed full consumption of the norbornene double bond signals of **Nor-O-SS** by photoinduced reaction with 2-mercaptoethanol, giving an initial proof of concept. Whereas the double bonds of **Nor-O-SS** were readily consumed within 5 min of irradiation, full conversion of **Nor-L-Cys** was retarded. Even with a SH:ene ration of 2:1, full conversion of **Nor-L-Cys (2x SH)** could not be observed within 10 min. Linkers (15 mM) were reacted with 2-mercaptoethanol (30 mM) in D_2O, using LiTPO as photoinitiator (0.6 mM, λ = 400-500 nm, 20 mW cm^{-2}).

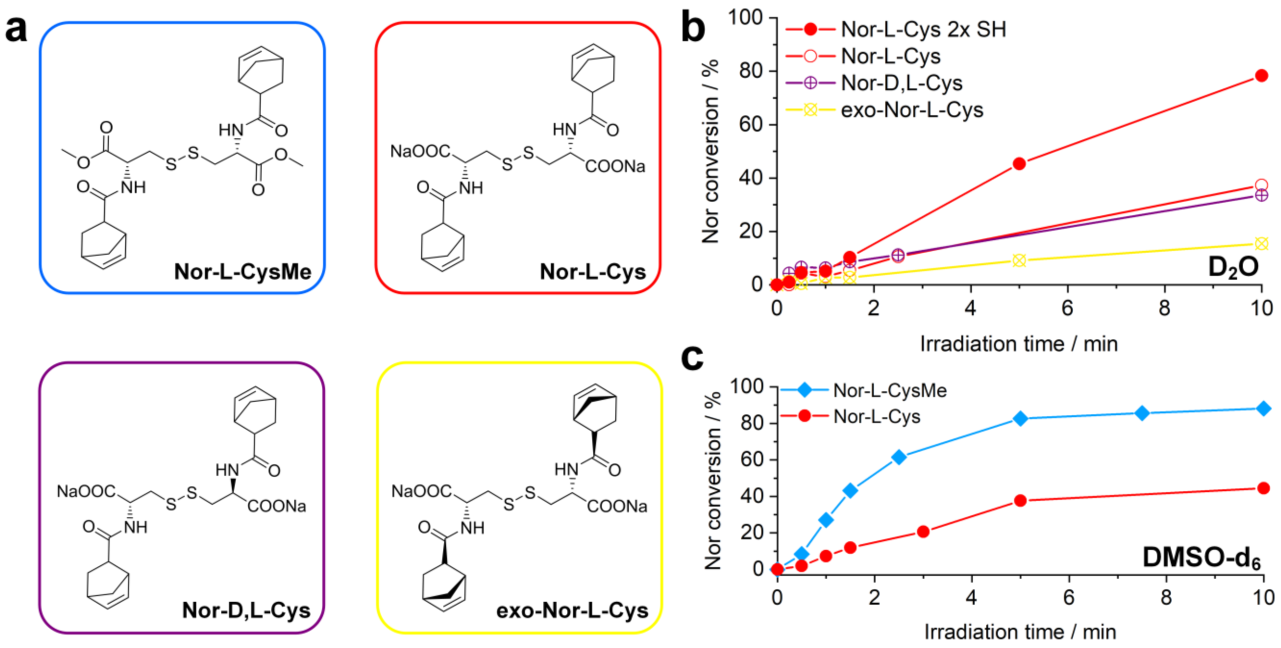

Figure S23. (a) Cystine-based linkers showed a strong retardation of TEC reaction in a reactivity estimation by ^{1}H-NMR. (b) **Nor-L-Cys** exhibited incomplete conversion (~37%) upon irradiation for 10 min, even when irradiated in presence of a double equivalent of thiols (60 mM, SH:ene=2:1, 78% conversion). **exo-Nor-L-Cys** showed the lowest reactivity with only ~15% ene-conversion after 10 min of irradiation. Moreover, changing the core configuration from *L*-cystine to *D,L*-cystine does not significantly impact the reactivity, as both **Nor-L-Cys** and **Nor-D,L-Cys** gave similar low ene-conversions. (c) To examine the effect of the carboxylic acid side groups on the reactivity, further tests were conducted in aprotic DMSO-d_6. Organosoluble linkers (15 mM) were reacted with 2-mercaptoethanol (30 mM, SH:ene=1:1) using TPO-L (0.6 mM) as photoinitiator. As in D_2O, **Nor-L-Cys** free acid showed relatively low norbornene conversion (~44%), whereas its methylated derivative **Nor-L-CysMe** yielded a final conversion of ~88% after 10 min of irradiation. The significant higher reactivity of **Nor-L-CysMe** indicates an involvement of the carboxylic acid groups of **Nor-L-Cys** in intramolecular interactions causing retardation.

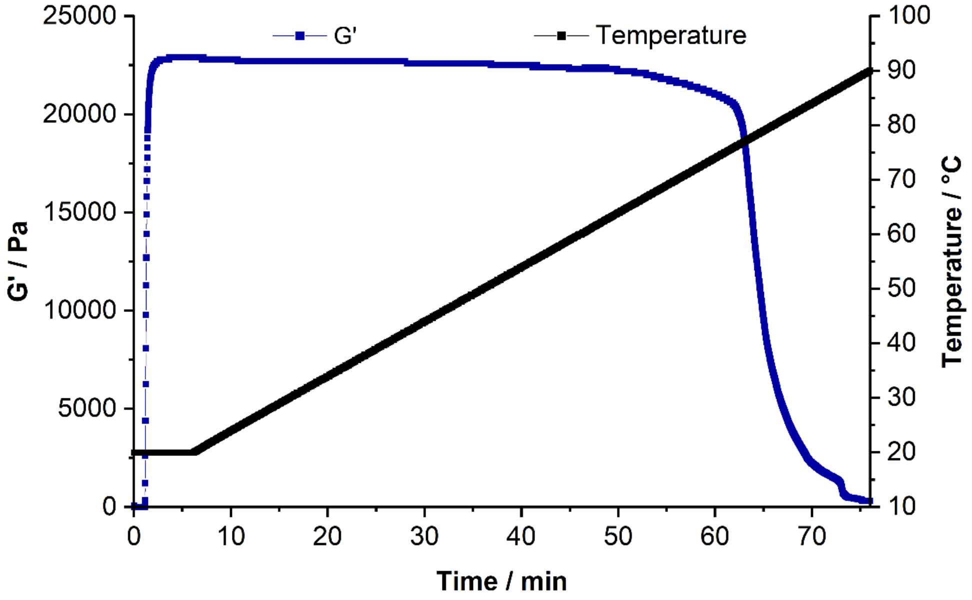

Figure S24. The thermostability of **8arm-D,L-HCys 100** hydrogel was investigated by oscillatory photorheology. After *in situ* photopolymerization at 20 °C the temperature was increased up to 90 °C at a heating rate of 1° C/min. Only at temperatures above 60 °C G' significantly changed, revealing stability of the hydrogel at physiological conditions.

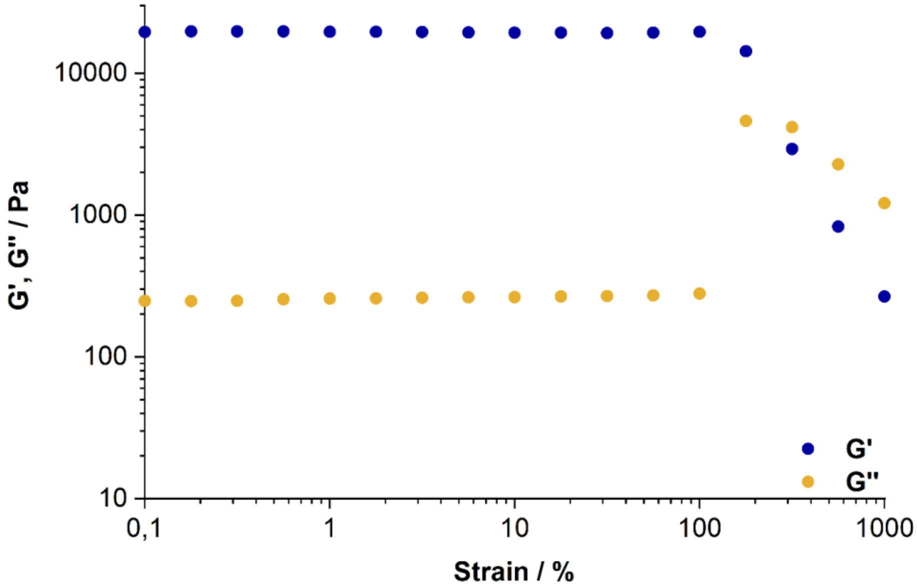

Figure S25. Mechanostability of disulfide-crosslinked **8arm-D,L-HCys 100** hydrogel. Disulfide-linked networks are reported to exhibit dynamic shear-thinning behavior, if a pendant free thiol is present in very close proximity to a crosslinking disulfide bond.[11] If a high amount of unreacted thiols were present in **8arm-D,L-HCys 100** hydrogel as well, reversible shear-thinning behavior would be observable. Hence, the shear-thinning behavior of **8arm-D,L-HCys 100** hydrogel was investigated, to exclude instability due to reversible bond formation. First an amplitude sweep was performed. After *in situ* photo-polymerization, the amplitude of the applied strain was logarithmically increased from 0.1% to 1000% at a constant frequency of 1 Hz. Up until a strain of 100% the hydrogel behaves like an elastic solid (linear viscoelastic regime) with the value of the storage modulus G' being two magnitudes above that of the loss modulus G''. Starting at a strain of ~300%, the loss modulus G'' surpasses the storage modulus G', indicating that the gel behaves like a liquid suggesting reverse gelation. However, inspection of the sample revealed rupture of the hydrogel.

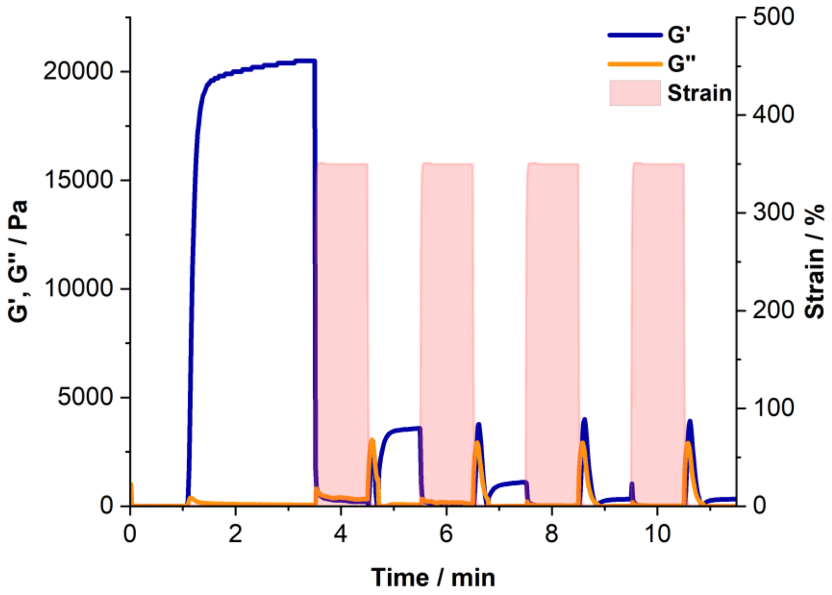

Figure S26. Dynamic strain amplitude cycling experiment, displaying storage- and loss-modulus (G', G'') of **8arm-D,L-HCys 100** hydrogel over time at alternating strains. After *in situ* polymerization, the strain was varied between 350% and 1% in 60 s periods. In phases of high strain G' was lower than G'', while when strain was released G' increased again. Nevertheless, no full recovery of G'_{max} but gradual reduction was observed with every cycle, after an initial peak when the strain was released, demonstrating that **8arm-D,L-HCys 100** hydrogel is not reversibly shear-thinning but damaged at high strains.

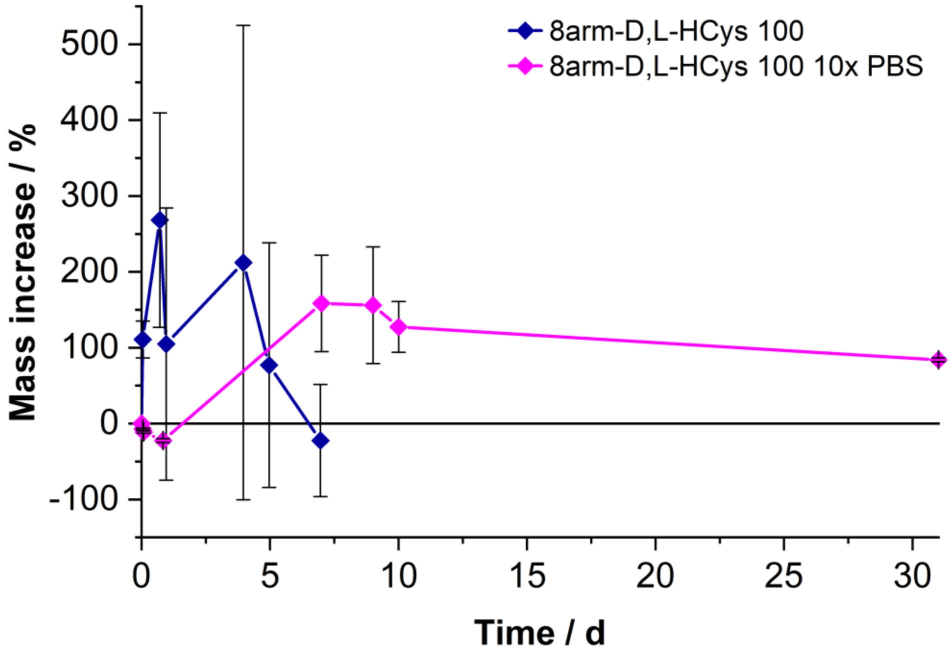

Figure S27. Impact of ion strength on hydrogel stability. When immersed in PBS, **8arm-D,L-HCys 100** hydrogel was deforming and swelling heavily leading finally to disintegration within a few days. On the contrary, when the hydrogel was immersed in 10x PBS in the initial 20 h, samples were first losing mass due to osmosis. After changing to normal PBS, samples were swelling as well, but remained their shape and did not dissolve anymore. These samples were monitored for more than a month but were stable much longer.

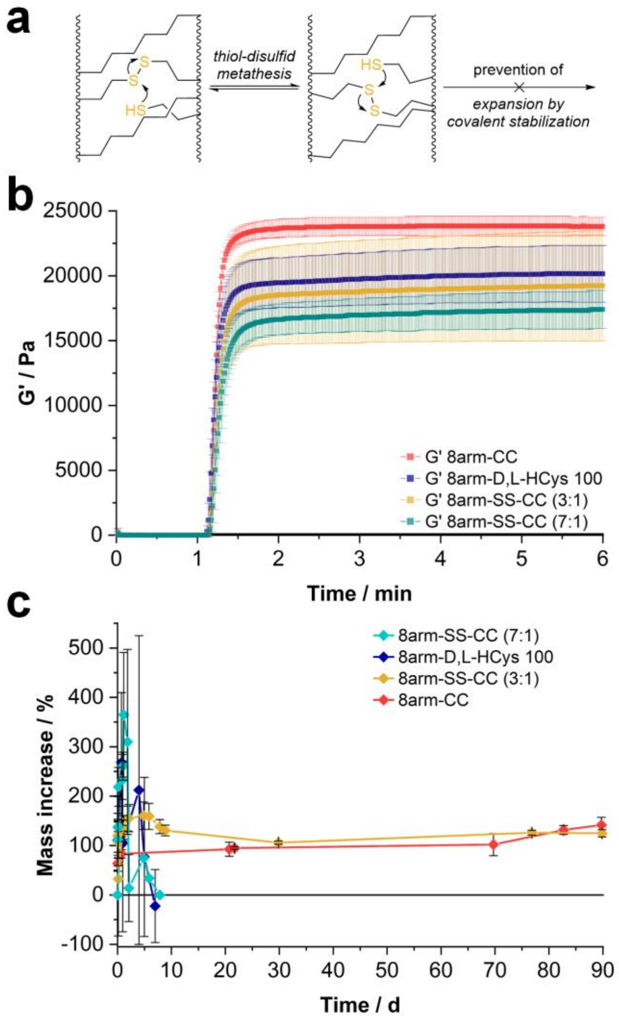

Figure S28. Stabilization of **8arm-D,L-HCys 100** hydrogel by a non-cleavable background network. (a) Introducing a non-cleavable background network inhibits detrimental swelling of **Nor-D,L-HCys 100** hydrogel. Whereas thiol-disulfide metathesis can still occur, dissolution of the hydrogel is prevented. To stabilize a hydrogel with an 8armPEG20k-SH backbone, at least two arms of the macromer have to be connected to an aliphatic **Nor-O-CC** linker unit to form a continuous non-cleavable network. (b) Photopolymerization of formulations based on 10 wt% 8armPEG20k-SH, combinations of **Nor-D,L-HCys** and **Nor-O-CC** at an equimolar thiol-ene ratio (22.2 mM) and 0.4 mM Li-TPO was monitored by photorheology. The linker ratios were varied from 100% **Nor-O-CC** (**8arm-CC**) to a mixture of 75% **Nor-D,L-HCys** and 25% **Nor-O-CC** (**8arm-SS-CC (3:1)**) as well as 87.5% **Nor-D,L-HCys** and 12.5% **Nor-O-CC** (**8arm-SS-CC (7:1)**). (c) Both non-stabilized **8arm-D,L-HCys 100** and **8arm-SS-CC (7:1)** hydrogels were disintegrating upon swelling in PBS within a few days. With 25% of **Nor-D,L-HCys** being substituted by **Nor-O-SS** (**8arm-SS-CC (3:1)**) the percolation threshold was met and the hydrogel was stabilized.

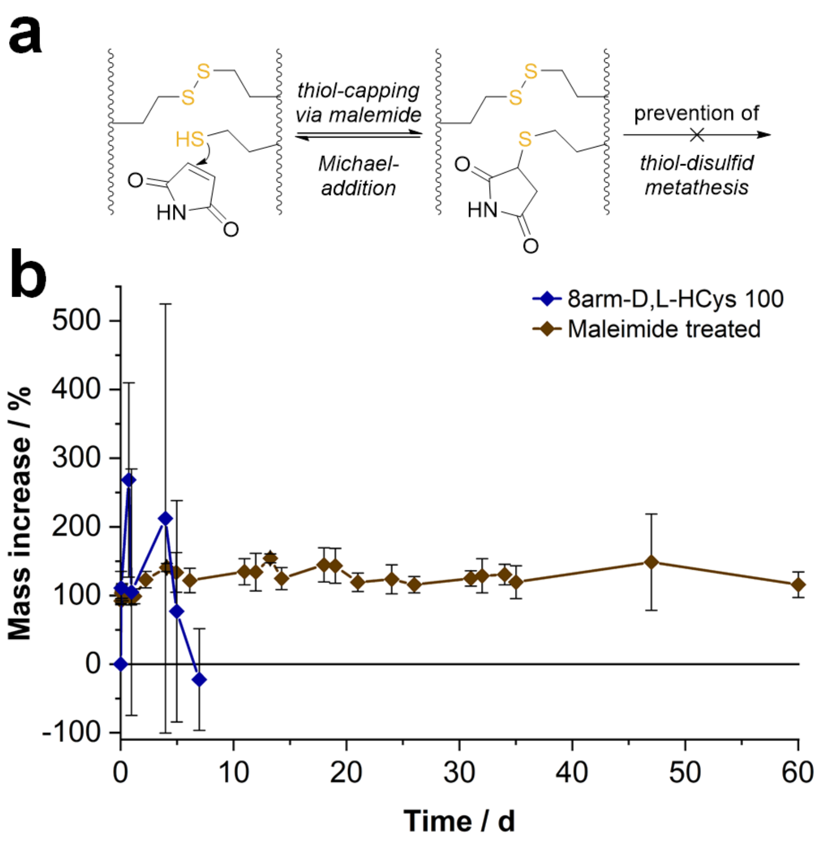

Figure S29. Capping of free thiols by maleimide. (a) Small molecule Michael-acceptors as maleimide can be used to irreversibly bind free thiols and thus prevent thiol-disulfide metathesis. (b) Unstable **8arm-D,L-HCys 100** hydrogel was stabilized by soaking in a solution of maleimide (4 mM) for 1 h prior to swelling in PBS. Following this treatment, samples were stable for more than 2 months.

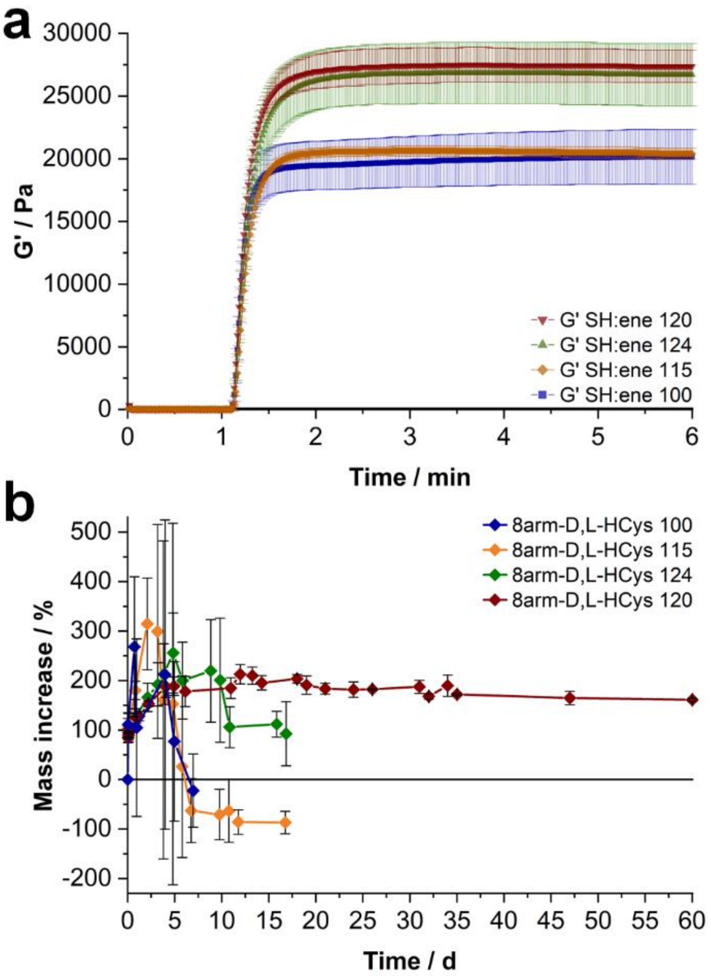

Figure S30. Consumption of free thiols by an excess of linker. (a) Photopolymerization of hydrogels based on 10wt% 8armPEG20k-SH and an excess of **Nor-D,L-HCys** was monitored by photorheology. Whereas G' of **8arm-D,L-HCys 115** hydrogel prepared with an ene-excess of 15% was in the same range as that of the equimolar **8arm-D,L-HCys 100** hydrogel, an increased excess (1:1.20, 1:1.24) lead to higher stiffness. (b) Swelling behavior of hydrogels formed with excess **Nor-D,L-HCys**. Whereas formulations with an ene-excess of 15 mol% (**8arm-D,L-HCys 115**) and 24 mol% (**8arm-D,L-HCys 124**) were not stabilized, using 20 mol% excess of ene successfully preserved long time hydrogel integrity. Hence, an ene-excess of 20% appears to be the optimum for this formulation (**8arm-D,L-HCys 120**). This experiment is of particular interest, since it shows, that **Nor-D,L-HCys** does not fully react when applied at an 1:1 thiol-ene ration. Consequently, an excess of ene is required here to consume unreacted thiols and even increase the crosslinking density.

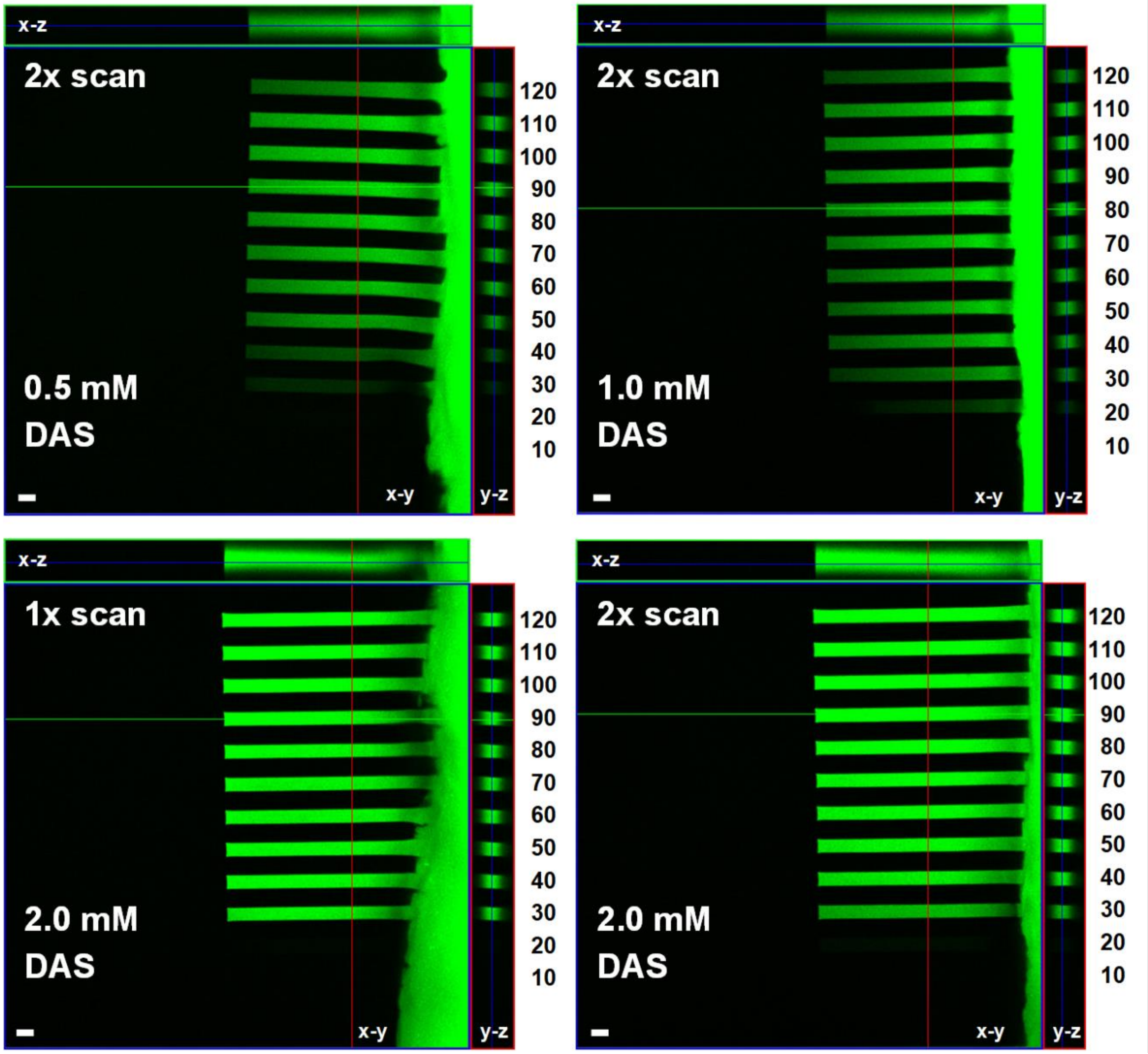

Figure S31: Two-photon micropatterning of **8arm-D,L-HCys 120** hydrogel. Top-down views of confocal z-stacks with corresponding orthogonal projections of channels (300 x 20 x 20 µm^3) fabricated at increasing laser powers (10–120 mW) in **8arm-D,L-HCys 120** hydrogel in presence of different concentrations of **DAS** ranging from 0.5-2.0 mM. Micro-channels were visualized by soaking in a solution of **FITC2000**. Scale bars = 20 µm.

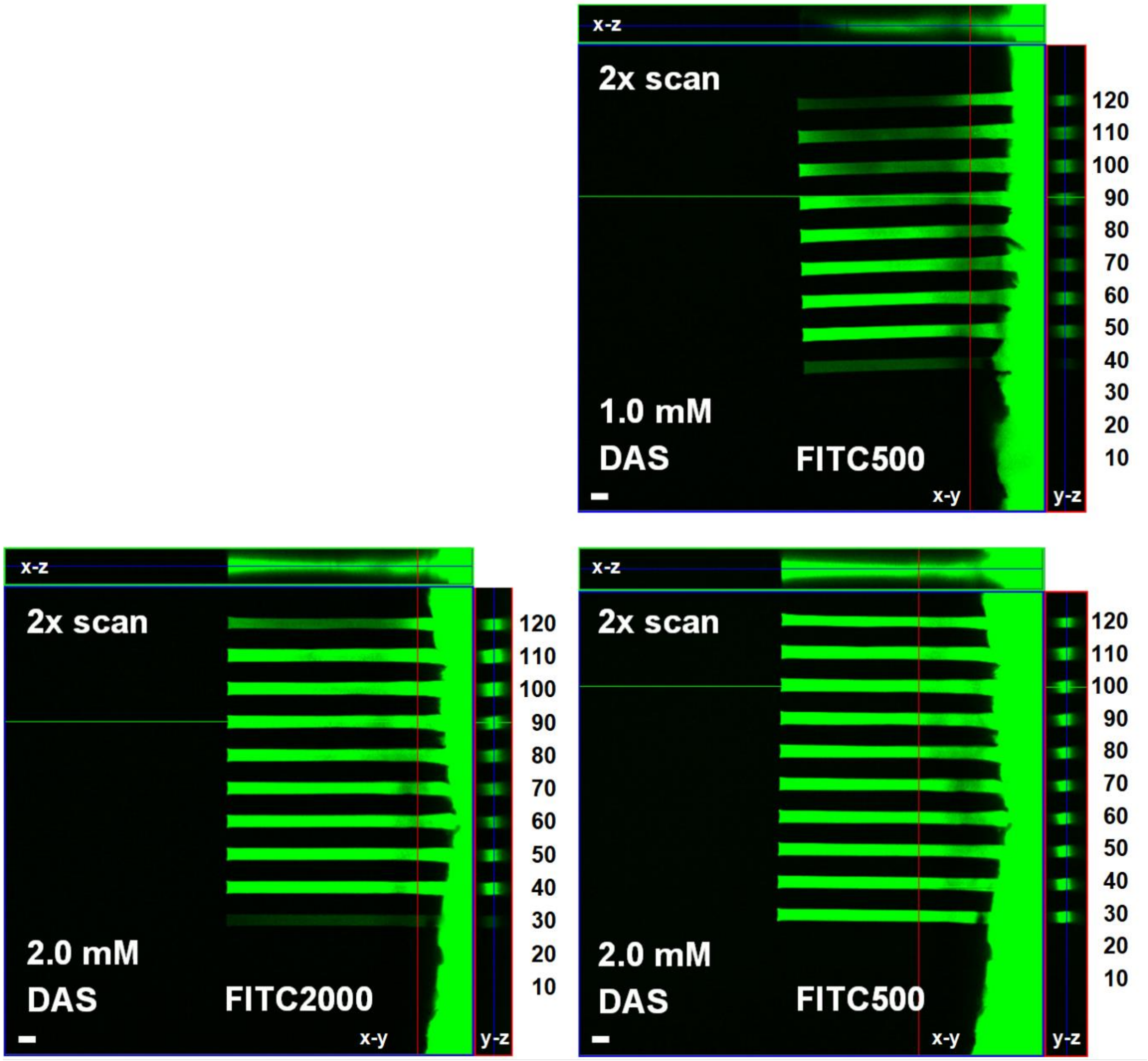

Figure S32. Two-photon micropatterning of **8arm-L-HCys 100** hydrogel. Top-down views of confocal z-stacks with corresponding orthogonal projections of channels (300 x 20 x 20 µm^3) fabricated at increasing laser powers (10–120 mW) in **8arm-L-HCys 100** in presence of 1.0 mM or 2.0 mM **DAS**. Micro-channels were visualized by soaking in solutions of either **FITC2000** (2 mM **DAS**) or **FITC500** (1.0 mM **DAS**). Scale bars = 20 µm.

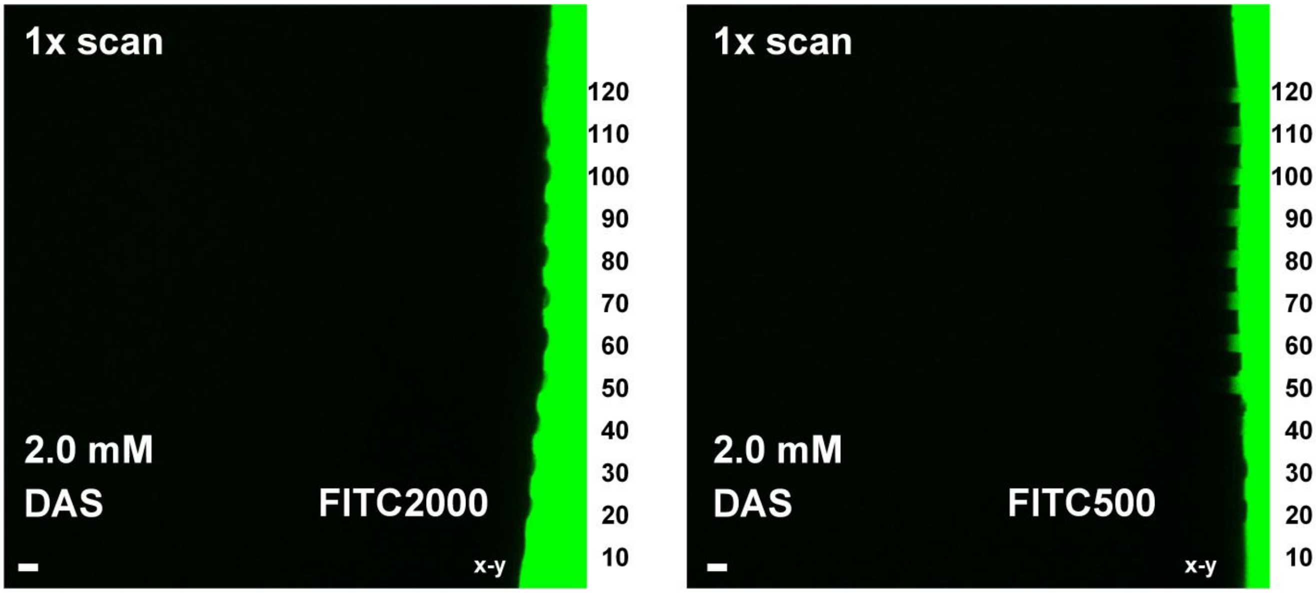

Figure S33. Two-photon micropatterning of **8arm-L-HCys 100** hydrogel. Top-down views of LSM images areas (300 x 20 x 20 µm^3) scanned only once at increasing laser powers (10–120 mW) in **8arm-L-HCys 100** in presence of 2.0 mM **DAS**. Hydrogels were soaked in solutions of either **FITC2000** or **FITC500** for one week. In both cases microchannels could not be visualized due to insufficient cleavage for the FITC-dextran to enter. When **FITC500** was used, the openings of channels micropatterned at laser powers above 40 mW were visible. At lower laser powers or when **FITC2000** was used, a bulge could be observed at the edge of the scanning area indicating local swelling. Scale bars = 20 µm.

COMPUTATIONAL STUDY

To get a better insight into the cause of this TEC retardation and further evaluate the structure-property relationship, the reaction of the retarded linker **Nor-L-Cys** and the elongated, highly-reactive **Nor-L-HCys** with a 2-mercatoethanol thiyl radical was computationally modelled. The attack of the thiyl radical on the acids **A** and **B** (Scheme S1) was chosen as a simplified model for the investigations.

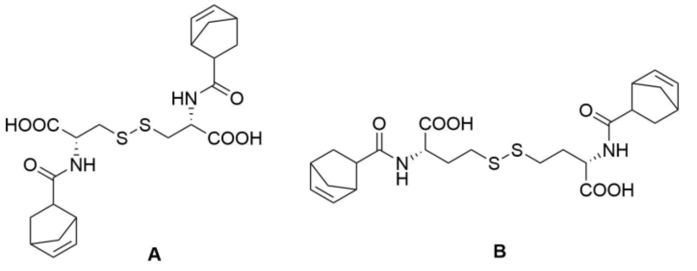

Scheme S1. The systems chosen for the computational studies of the reaction with the thiyl radical. The acids **A** and **B** reflect the systems **Nor-L-Cys** and **Nor-L-HCys**.

The thiyl radical can attack the double bond of the norbornene fragment yielding two different radical products **p1** and **p2**. Moreover, a new chiral center appears simultaneously with the formation of the new C-S bond. Thus, for each of the systems **A** and **B** four possible radical products have to be taken into account: **p1_R**, **p1_S**, **p2_R** and **p2_S**. Scheme S2 illustrates the computed reaction pathways for system **A**. Figure S34 presents the computed free energy profiles for the aforementioned reaction pathways for both systems **A** and **B** in comparison.

Since the studied systems are highly flexible conformationally, the allowed conformational space was carefully explored. The free energies of the reactants, transition states, and products depicted in Figure S34 correspond to the most favorable conformations. One can see that system **B** leads to the most stabilized thermodynamically product **B_p2_R**, with ΔG = -4.2 kcal mol^{-1}. The energetically most favorable product in the case of system **A** is **A_p2_S**, with ΔG = -1.7 kcal mol^{-1}. Thus, the thermodynamic driving force of the computationally modeled event is 2.5 times larger for system **B** as compared to system **A**. This agrees well with the experimentally observed reactivity difference for **Nor-L-Cys** (**A**) and **Nor-L-HCys** (**B**).

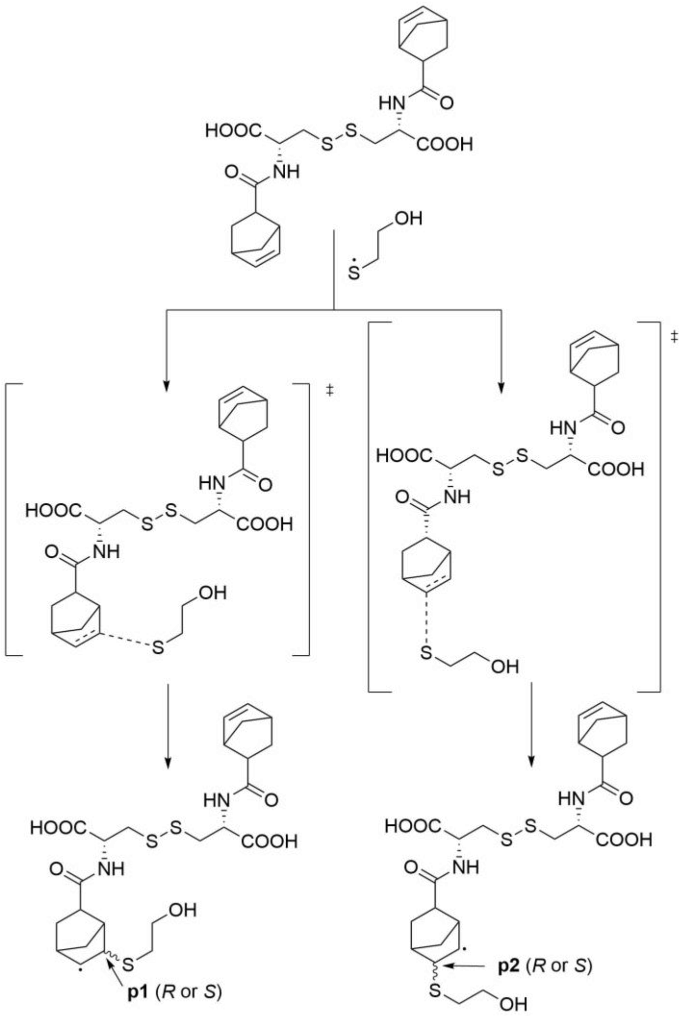

Scheme S2. Possible reaction pathways for the attack of the thiyl radical on the system **A** yielding the radical products **p1** and **p2**.

The high conformational flexibility of the computed molecules allows the presence of manifold intramolecular interactions. Figure S34 also shows the optimized structures of the products **A_p1_S** and **B_p2_R**. In both structures the characteristic stabilizing hydrogen bonds are visible. System **A** is more rigid compared to the elongated, and therefore, even more flexible system **B**. Thus, system **B** has a higher degree of freedom for the formation of those stabilizing intermolecular interactions. Being proven by the computed thermodynamic properties, this explains the experimentally observed reactivity difference.

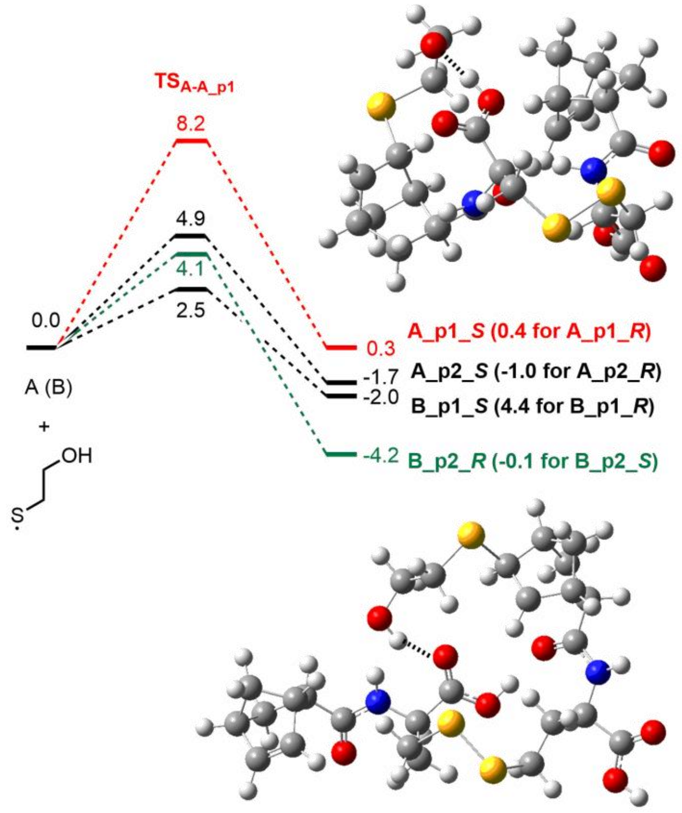

Figure S34. Computed reaction profiles ((U)B3LYP-D3/def2-TZVP, $\Delta G_{298,H2O}$) for the formation of the radical products **p1** and **p2** for the systems **A** and **B** including the diastereomeric pathways. The sum of the reactant energies is taken as a reference (0.0 kcal mol^{-1}). The optimized structures of the products **A_p1_S** and **B_p2_R** are also shown.

Computational details

The conformational space of all flexible molecules has been initially searched using the OPLS_2005 force field[12] and the systematic Monte Carlo conformers search routine implemented in MACROMODEL 11.5.[13]

Accordingly, the structures located at force field level have then been subjected to (U)B3LYP-D3/def2-TZVP[14-19] geometry optimization. The nature of all stationary points (minima and transition states) was verified through the computation of the vibrational frequencies. Gibbs free energies (G_{298}) at 298.15 K were evaluated using a quasi-RRHO treatment of vibrational frequencies and *GoodVibes* program.[20, 21] All energies are reported in kcal mol^{-1}.

The density-based solvation model SMD[22] was applied to consider solvent (DCM) effects. Free energies in solution have been corrected to a reference state of 1 mol L^{-1} at 298.15 K through addition of RTln(24.46) = +7.925 kJ mol^{-1} to the gas phase (1 atm) free energies.

The DFT calculations have been performed with the Gaussian16 program package.[23]

REFERENCES

1. T. Majima, W. Schnabel and W. Weber, *Die Makromolekulare Chemie*, 1991, **192**, 2307-2315.
2. M. Tromayer, A. Dobos, P. Gruber, A. Ajami, R. Dedic, A. Ovsianikov and R. Liska, *Polym. Chem.*, 2018, **9**, 3108-3117.
3. S. Muthusamy, B. Gnanaprakasam and E. Suresh, *Org. Lett.*, 2006, **8**, 1913-1916.
4. C.-G. Chae, Y.-G. Yu, H.-B. Seo, M.-J. Kim, R. H. Grubbs and J.-S. Lee, *Macromolecules*, 2018, **51**, 3458-3466.
5. M. Mackiewicz, K. Kaniewska, J. Romanski, E. Augustin, Z. Stojek and M. Karbarz, *Journal of Materials Chemistry B*, 2015, **3**, 7262-7270.
6. F. N. Behrendt and H. Schlaad, *Macromol. Rapid Commun.*, 2018, **39**, 1700735.
7. C. Gorsche, R. Harikrishna, S. Baudis, P. Knaack, B. Husar, J. Laeuger, H. Hoffmann and R. Liska, *Anal. Chem.*, 2017, **89**, 4958-4968.
8. A. Ovsianikov, S. Mühleder, J. Torgersen, Z. Li, X.-H. Qin, S. Van Vlierberghe, P. Dubruel, W. Holnthoner, H. Redl, R. Liska and J. Stampfl, *Langmuir*, 2014, **30**, 3787-3794.
9. W. Lwowski, *Angew. Chem.*, 1958, **70**, 483-495.
10. K.-D. Gundermann, *Angew. Chem.*, 1963, **75**, 1194-1203.
11. G. A. Barcan, X. Zhang and R. M. Waymouth, *J. Am. Chem. Soc.*, 2015, **137**, 5650-5653.
12. J. L. Banks, H. S. Beard, Y. Cao, A. E. Cho, W. Damm, R. Farid, A. K. Felts, T. A. Halgren, D. T. Mainz, J. R. Maple, R. Murphy, D. M. Philipp, M. P. Repasky, L. Y. Zhang, B. J. Berne, R. A. Friesner, E. Gallicchio and R. M. Levy, *J. Comput. Chem.*, 2005, **26**, 1752-1780.
13. S. MacroModel, LLC, New York, NY, 2018.
14. A. D. Becke, *The Journal of Chemical Physics*, 1993, **98**, 5648-5652.
15. C. Lee, W. Yang and R. G. Parr, *Physical Review B*, 1988, **37**, 785-789.
16. S. H. Vosko, L. Wilk and M. Nusair, *Can. J. Phys.*, 1980, **58**, 1200-1211.
17. P. J. Stephens, F. J. Devlin, C. F. Chabalowski and M. J. Frisch, *The Journal of Physical Chemistry*, 1994, **98**, 11623-11627.
18. S. Grimme, J. Antony, S. Ehrlich and H. Krieg, *The Journal of Chemical Physics*, 2010, **132**, 154104.
19. F. Weigend and R. Ahlrichs, *Phys. Chem. Chem. Phys.*, 2005, **7**, 3297-3305.
20. R. S. Paton and I. Funes-Ardois, *GoodVibes.Py*.
21. S. Grimme, *Chem. Eur. J.*, 2012, **18**, 9955-9964.
22. A. V. Marenich, C. J. Cramer and D. G. Truhlar, *The Journal of Physical Chemistry B*, 2009, **113**, 6378-6396.
23. M. J. T. Frisch, G. W.; Schlegel, H. B.; Scuseria, G. E.; Robb, M. A.; Cheeseman, J. R.; Scalmani, G.; Barone, V.; Mennucci, B.; Petersson, G. A.; et al. Gaussian 16, Revision A.03. Gaussian, Inc., Wallingford CT, 2016.

APPENDIX

Coordinates of the most stable ($\Delta G_{298,DCM}$) conformations as computed at the (U)B3LYP-D3/def2-TZVP level of theory

A

S 0.16348 3.15030 -0.21860

S -1.63124 2.14767 -0.29444

C -1.86080 1.42803 1.38414

C -1.62228 -0.08949 1.48145

N -2.53624 -0.84523 0.65357

C -3.84363 -0.98904 0.95554

O -4.30508 -0.61031 2.03788

C -4.67691 -1.60971 -0.14581

H -4.14355 -2.48544 -0.51808

C -4.87046 -0.60945 -1.35965

C -5.52922 0.63274 -0.79005

C -6.78173 0.30406 -0.46160

C -6.97982 -1.15987 -0.80626

H -8.00868 -1.49834 -0.90041

C -6.07376 -1.27902 -2.04933

H -6.43887 -0.70570 -2.90227

H -5.88219 -2.31106 -2.34943

C -6.13435 -1.96981 0.23301

H -6.30917 -3.03906 0.11263

H -6.37666 -1.69722 1.25794

H -7.49849 0.91324 0.07279

H -5.01487 1.55961 -0.57498

H -3.96620 -0.45933 -1.94349

H -2.21502 -1.10836 -0.26700

C -0.19398 -0.45282 1.12288

O 0.10318 -1.13604 0.16083

O 0.68069 0.09024 1.94976

H 1.62531 -0.10182 1.64959

H -1.77727 -0.36503 2.52700

H -2.89999 1.62409 1.64683

H -1.22107 1.96765 2.07865

C 1.43043 1.96600 -0.85781

C 2.76337 2.01305 -0.07711

N 3.61975 0.91520 -0.47402

C 3.76260 -0.22939 0.21208

O 3.13578 -0.46454 1.26112

C 4.71844 -1.22711 -0.38894

H 5.42711 -0.69018 -1.01879

C 5.47431 -2.12478 0.62682

H 5.12876 -1.97838 1.64817

H 6.54399 -1.91827 0.59265

C 5.17184 -3.57317 0.11119

C 3.70187 -3.81699 0.38733

C 2.99972 -3.05086 -0.45173

C 3.99024 -2.29161 -1.31187

H 3.60978 -1.86304 -2.23520

C 5.12974 -3.32688 -1.41084

H 4.84038 -4.21181 -1.97840

H 6.05721 -2.91426 -1.81260

H 1.93255 -2.88429 -0.45094

H 3.31880 -4.40456 1.21100

H 5.86470 -4.32570 0.47935

H 4.17480 1.04893 -1.30756

C 3.49720 3.31901 -0.34406

O 4.41450 3.44002 -1.12435

O 2.97922 4.32816 0.35946

H 3.42158 5.15968 0.11090

H 2.56465 1.93631 0.99008

H 1.61208 2.17501 -1.91202

H 1.02658 0.96237 -0.76854

B

S 0.72379 -2.04023 2.44690

S 2.05541 -3.23127 1.42442

C 3.06548 -1.70992 -0.67672

H 3.11233 -2.60174 -1.30217

C -0.87018 -1.89866 0.09382

H -1.57456 -2.61058 0.52428

C -1.65102 -1.00395 -0.89773

H -1.94554 -1.62086 -1.74747

C 4.04391 -0.66119 -1.24854

H 3.83979 -0.56454 -2.31488

C 5.48290 -1.11299 -1.10006

N 3.88275 0.64104 -0.63417

C 3.00746 1.55854 -1.06472

H 4.41055 0.82109 0.20671

O 2.30722 1.37369 -2.07817

C 2.96731 2.86366 -0.29524

C 1.63718 3.65497 -0.44508

C 3.12370 2.73511 1.26765

H 3.80170 3.45415 -0.68312

C 1.19312 3.86665 1.03642

H 0.89473 3.11208 -1.02327

H 1.81168 4.61549 -0.92973

C 2.00194 1.81908 1.72765

H 4.12827 2.48364 1.60004

C 0.85636 2.48967 1.58036

H 0.43371 4.63398 1.16524

H 2.12639 0.78003 1.99755

H -0.14583 2.10089 1.69827

C 2.56530 4.10420 1.70067

H 3.11090 4.94929 1.27634

H 2.50737 4.20634 2.78484

N -2.83925 -0.41948 -0.31088

H -2.75888 0.51696 0.05464

C -3.99831 -1.08730 -0.18396

O -4.13298 -2.24562 -0.60486

C -5.11829 -0.38281 0.55517

C -5.21303 1.17237 0.45448

C -6.54242 -0.84989 0.09578

H -4.98254 -0.66944 1.60218

C -6.68408 1.39253 -0.02487

H -4.50500 1.60885 -0.24853

H -5.05084 1.63562 1.42759

C -6.65943 -0.47818 -1.37280

H -6.77070 -1.87885 0.35635

C -6.74364 0.85310 -1.44228

H -7.04877 2.40632 0.12151

H -6.56229 -1.17405 -2.19420

H -6.72817 1.46493 -2.33447

C -7.40021 0.25857 0.73673

H -7.28484 0.32471 1.82056

H -8.45688 0.17336 0.48137

O 6.31195 -0.57598 -0.39873

O 5.72453 -2.21106 -1.82515

H 6.64361 -2.49655 -1.67897

C -0.75769 0.10816 -1.41850

O -0.94762 1.28859 -1.20205

O 0.28120 -0.36715 -2.09286

H 0.98891 0.32923 -2.19136

H 2.06704 -1.29236 -0.77773

C 3.33535 -2.08155 0.77699

H 3.36032 -1.20950 1.43008

H 4.28526 -2.61040 0.88382

C -0.19056 -1.08392 1.18091

H -0.91842 -0.52807 1.77789

H 0.50357 -0.36125 0.75557

H -0.12570 -2.46293 -0.46681

Thyil Radical

S 1.47797 -0.30766 0.06557

C 0.14361 0.86265 -0.26098

C -1.20731 0.47126 0.32037

O -1.70267 -0.75877 -0.20807

H -1.08829 -1.46007 0.05029

H -1.14628 0.42599 1.41254

H -1.93844 1.23616 0.05223

H 0.07987 0.93742 -1.35386

H 0.44924 1.84982 0.09783

B_p1_S

S 0.98700 -5.08153 0.06008

S -0.95717 -4.76824 0.66932

C -1.40084 -3.09273 0.07278

C -1.41715 -2.93835 -1.44225

H -2.21739 -3.53925 -1.87612

H -0.47460 -3.29743 -1.85502

C -1.59544 -1.48276 -1.91391

N -2.87764 -0.94194 -1.51579

C -3.32383 0.23713 -1.98249

O -2.67613 0.88001 -2.82269

C -4.64063 0.78747 -1.47759

H -5.15061 1.11707 -2.38359

C -5.61094 -0.09507 -0.63442

H -5.21819 -1.07300 -0.35973

H -6.53796 -0.26366 -1.18452

C -5.87564 0.78710 0.62463

C -4.60131 0.81427 1.40722

C -3.63054 1.66901 0.67230

H -2.69291 1.16136 0.44562

C -4.44667 2.05579 -0.58508

H -4.04160 2.90294 -1.13163

C -5.86346 2.19862 -0.00509

H -5.94394 2.99904 0.73059

H -6.62687 2.33305 -0.77248

S -3.12100 3.15373 1.68639

C -1.52621 3.58122 0.89310

C -0.29389 3.10990 1.64675

O -0.25581 1.69118 1.84953

H -0.37261 1.23967 0.99349

H 0.59667 3.43244 1.09542

H -0.25845 3.55542 2.64106

H -1.53698 3.17593 -0.12036

H -1.48303 4.66852 0.80881

H -4.33698 0.14595 2.21429

H -6.76495 0.49566 1.17951
H -3.37361 -1.38740 -0.76097
C -0.44092 -0.62177 -1.40978
O -0.54030 0.21490 -0.53426
O 0.69510 -0.92372 -2.01793
H 1.47029 -0.48917 -1.56072
H -1.53309 -1.47372 -3.00290
H -2.39106 -2.93973 0.51041
H -0.72268 -2.38657 0.54807
C 1.98875 -3.97353 1.13741
C 2.42679 -2.70951 0.39971
H 1.59166 -2.31395 -0.16825
H 3.21329 -2.95912 -0.31195
C 2.96470 -1.61665 1.34976
N 4.03352 -0.81503 0.77470
C 3.87510 0.04448 -0.24409
O 2.81436 0.13085 -0.88175
C 5.05021 0.94924 -0.51741
H 5.96588 0.41072 -0.27317
C 5.12420 1.54433 -1.94624
H 4.26925 1.26287 -2.55760
H 6.03169 1.21289 -2.45106
C 5.17447 3.08826 -1.68507
C 3.81083 3.47046 -1.14309
C 3.70948 2.96914 0.09080
C 5.00794 2.24758 0.39504
H 5.21957 2.03977 1.44039
C 6.00782 3.12181 -0.38735
H 6.10418 4.12340 0.03309
H 6.99418 2.66551 -0.49121
H 2.83130 2.94656 0.72053
H 3.02919 3.94578 -1.72058
H 5.53779 3.66744 -2.53035
H 4.90072 -0.76850 1.28759
C 1.80646 -0.77069 1.88360
O 0.71898 -1.25893 2.12561
O 2.08800 0.51008 2.07140
H 1.22621 1.01787 2.17942
H 3.40017 -2.09918 2.22747
H 2.85324 -4.55286 1.46720
H 1.38051 -3.74450 2.00955

B_p1_R

S -0.64456 4.68751 1.08356
S 0.69423 3.18038 1.50650
C 2.21932 3.62909 0.57420
C 3.42993 2.81160 1.03639
H 3.46937 2.76994 2.12629
H 4.32019 3.35178 0.71256
C 3.57450 1.37021 0.50918
N 2.54513 0.47375 0.98971
C 2.77890 -0.82506 1.25675
O 3.90995 -1.31648 1.10738
C 1.67949 -1.62145 1.92653
H 2.06709 -1.68205 2.94883
C 0.22494 -1.06865 2.00540
H -0.00540 -0.30662 1.26487
H 0.01878 -0.65174 2.99199
C -0.63488 -2.34466 1.74040
C -0.50793 -2.64689 0.28359
C 0.85886 -3.21546 0.05896
H 0.81416 -4.28175 -0.19783

C 1.48741 -3.09425 1.47826
H 2.37068 -3.71007 1.61253
C 0.25901 -3.45426 2.33955
H -0.12025 -4.45822 2.14578
H 0.44226 -3.33615 3.40881
S 1.68601 -2.43462 -1.38535
C 2.88750 -3.70670 -1.92432
C 4.31390 -3.53111 -1.42589
O 4.46392 -3.74922 -0.02622
H 4.19639 -2.92967 0.43839
H 4.94125 -4.27160 -1.93077
H 4.68574 -2.53819 -1.69988
H 2.50036 -4.68564 -1.63399
H 2.89396 -3.66271 -3.01590
H -1.15839 -2.27910 -0.49647
H -1.65201 -2.26267 2.11465
H 1.59124 0.79993 1.00316
C 3.70208 1.34679 -1.01012
O 2.89334 0.88581 -1.78990
O 4.84416 1.90885 -1.40787
H 4.88667 1.90362 -2.38070
H 4.52693 0.99591 0.88367
H 2.02792 3.49922 -0.49180
H 2.40842 4.68431 0.76524
C -1.18310 4.47473 -0.65793
C -2.16278 3.34880 -0.97770
H -2.45877 3.46227 -2.02477
H -3.06299 3.45698 -0.37055
C -1.63129 1.93377 -0.76589
N -2.60192 0.92028 -1.14392
C -3.12797 0.02477 -0.27688
O -2.85591 0.03508 0.92690
C -4.10518 -0.95090 -0.89336
H -3.81775 -1.11390 -1.93213
C -4.24830 -2.30579 -0.15191
H -3.67347 -2.33522 0.77094
H -3.91555 -3.12597 -0.78799
C -5.78893 -2.40230 0.11430
C -6.10324 -1.33543 1.14443
C -5.98120 -0.14715 0.54661
C -5.59096 -0.40192 -0.89625
H -5.74325 0.41528 -1.59636
C -6.33533 -1.72642 -1.16002
H -7.41921 -1.60618 -1.14913
H -6.02542 -2.22119 -2.08259
H -6.01806 0.82818 1.01166
H -6.26081 -1.52360 2.19809
H -6.13718 -3.40756 0.33845
H -2.87281 0.86762 -2.11546
C -0.33941 1.66227 -1.53367
O 0.04661 2.31202 -2.48562
O 0.31142 0.61575 -1.04662
H 1.21962 0.55615 -1.43992
H -1.41500 1.76840 0.28547
H -0.29230 4.42871 -1.27964
H -1.66586 5.43449 -0.85967

B_p2_R

S -0.42779 -1.25801 2.03320
S 0.53545 -3.05269 2.33231
C 1.63263 -3.28173 0.87750
C 2.65817 -2.17079 0.73311

H 3.28903 -2.11236 1.62240
H 2.13861 -1.21726 0.63432
C 3.56730 -2.29253 -0.51436
N 4.33982 -1.07503 -0.69046
C 3.81843 0.04470 -1.21219
O 2.71689 0.05876 -1.78875
C 4.61968 1.30340 -0.98311
H 5.67911 1.08441 -1.12330
C 4.17413 2.54082 -1.80773
H 3.35379 2.31242 -2.48558
H 5.00805 2.91766 -2.39915
C 3.77607 3.57919 -0.73191
H 3.73839 4.60119 -1.10309
C 4.81308 3.27638 0.36517
H 4.66117 3.84431 1.28285
H 5.84085 3.40316 0.02282
C 4.40238 1.79697 0.50841
C 2.92680 1.91824 0.70639
C 2.46009 3.09223 -0.07956
H 1.69617 2.84921 -0.81894
S 1.70697 4.42176 1.00532
C 0.22089 3.52119 1.55816
C -0.90391 3.51778 0.52964
O -1.91559 2.56377 0.88504
H -1.54164 1.69152 0.69282
H -1.38928 4.49343 0.49283
H -0.51715 3.29251 -0.46645
H 0.50712 2.49529 1.79482
H -0.10981 3.99177 2.48491
H 2.28269 1.14817 1.10332
H 4.93604 1.18828 1.23434
H 5.19552 -0.99842 -0.15697
C 4.52154 -3.45880 -0.36860
O 5.65142 -3.36828 0.06010
O 3.95539 -4.61421 -0.72970
H 4.57525 -5.34489 -0.55570
H 2.95481 -2.45563 -1.39910
H 2.10313 -4.24583 1.08143
H 1.02887 -3.38369 -0.02217
C -2.03584 -1.71994 1.27189
C -1.99705 -2.25418 -0.15429
H -2.94133 -2.76790 -0.34099
H -1.20879 -2.99815 -0.26816
C -1.85794 -1.19327 -1.26949
N -2.92250 -0.20811 -1.23112
C -4.20657 -0.50579 -1.51447
O -4.53053 -1.58482 -2.02380
C -5.19622 0.57110 -1.11896
H -4.82351 1.52577 -1.49293
C -5.32123 0.68355 0.45715
C -5.74365 -0.68796 0.94953
C -7.00529 -0.88092 0.55447
C -7.44553 0.35619 -0.20542
H -8.51774 0.49831 -0.31601
C -6.65669 1.44432 0.55236
H -6.99026 1.57651 1.58234
H -6.64377 2.40773 0.03900
C -6.66147 0.33705 -1.56039
H -7.00256 1.14608 -2.20657
H -6.78942 -0.60288 -2.09327
H -7.58263 -1.79244 0.63384
H -5.08015 -1.40761 1.40925

H -4.44797 1.12565 0.92917

H -2.73169 0.66410 -0.76103

C -0.51666 -0.48305 -1.22863

O -0.36728 0.66779 -0.85797

O 0.46470 -1.26028 -1.64748

H 1.34761 -0.77688 -1.62878

H -1.90516 -1.72208 -2.22146

H -2.62562 -0.80195 1.33943

H -2.50208 -2.45115 1.93020

B_p2_S

S 2.88499 -0.95873 2.54461

S 1.87560 -2.74886 2.56843

C 1.06182 -2.84639 0.91925

C -0.43498 -2.57287 1.02712

H -0.60681 -1.63173 1.55127

H -0.91014 -3.36482 1.60954

C -1.15074 -2.49816 -0.33320

N -2.56477 -2.26481 -0.09564

C -3.42593 -1.98334 -1.08702

O -3.04508 -1.94018 -2.26571

C -4.88482 -1.77272 -0.73590

H -5.36840 -2.71920 -0.99616

C -5.27998 -1.36559 0.71855

H -4.43099 -1.18574 1.37681

H -5.88160 -2.14595 1.18221

C -6.12985 -0.08949 0.52028

H -6.75670 0.15738 1.37372

C -6.84864 -0.41673 -0.80068

H -7.44437 0.41078 -1.18728

H -7.46767 -1.31307 -0.73354

C -5.54207 -0.63823 -1.59492

C -4.81913 0.63706 -1.30068

C -5.22194 1.08621 0.06904

H -5.79965 2.01687 0.05465

S -3.83279 1.41825 1.25351

C -3.48699 3.17903 0.92317

C -3.00870 3.48537 -0.48668

O -1.86334 2.70834 -0.87328

H -2.09246 1.76866 -0.78802

H -2.69682 4.52875 -0.54154

H -3.81115 3.33075 -1.21084

H -4.37756 3.77390 1.13520

H -2.71643 3.44646 1.64854

H -5.63084 -0.89836 -2.64590

H -2.88350 -2.25338 0.85996

C -0.50218 -1.39491 -1.15956

O 0.30380 -1.61770 -2.03908

O -0.81736 -0.17425 -0.73782

H -0.18155 0.49138 -1.09551

H -1.00803 -3.42359 -0.89208

H 1.57401 -2.13354 0.27545

H 1.25086 -3.84739 0.52922

C 1.57027 0.33021 2.63493

C 2.16718 1.73529 2.50078

H 3.06566 1.83642 3.11300

H 1.43627 2.43381 2.90743

C 2.50277 2.21320 1.07679

N 3.60282 1.48487 0.48152

C 4.28896 1.96748 -0.57603

O 4.11398 3.12360 -0.98774

C 5.27241 1.04952 -1.26374

H 5.94302 1.71524 -1.80447

C 4.59012 0.10388 -2.33733

C 3.65561 -0.83832 -1.60218

C 4.41192 -1.68666 -0.89949

C 5.86240 -1.32134 -1.14674

H 6.59990 -2.08408 -0.90948

C 5.78054 -0.83907 -2.60990

H 5.52892 -1.63826 -3.30776

H 6.67823 -0.31839 -2.94913

C 6.09933 0.04141 -0.41183

H 7.15492 0.31233 -0.43590

H 5.79611 -0.00440 0.63236

H 4.07216 -2.41817 -0.17938

H 2.58076 -0.74320 -1.57383

H 4.16983 0.65007 -3.17745

H 3.65446 0.49106 0.66330

C 1.27129 2.19523 0.16957

O 1.22404 1.59111 -0.89036

O 0.27159 2.90322 0.65796

H -0.53817 2.85379 0.05516

H 2.79453 3.26202 1.15072

H 0.84838 0.13429 1.84302

H 1.06691 0.22917 3.59507

H -4.01637 1.05550 -1.88779

A_p1_S

S -3.38292 -2.39452 -1.61344

S -1.98286 -3.83620 -1.09208

C -0.45148 -3.19280 -1.86694

H 0.25969 -4.02044 -1.81467

H -0.66141 -3.00610 -2.91880

C -3.99083 -1.70839 -0.03286

H -4.82406 -1.07735 -0.34630

H -4.39504 -2.52387 0.56135

C -3.00201 -0.90762 0.81090

H -2.16497 -1.55868 1.07895

C 0.10820 -1.93813 -1.22130

H -0.69903 -1.25313 -0.99885

C 1.09458 -1.21575 -2.14206

N 0.84138 -2.21797 0.00975

C 0.94782 -1.31894 1.01231

H 1.57618 -2.90559 -0.07891

O 0.18135 -0.35482 1.11290

C 2.06555 -1.45902 2.02262

C 3.04982 -2.66630 1.99446

C 3.01969 -0.22311 1.90767

H 1.57496 -1.40224 2.99474

C 4.44520 -1.97599 1.86632

H 2.88407 -3.37806 1.18686

H 2.98766 -3.22209 2.93085

C 3.60041 -0.20103 0.47200

H 2.54803 0.70713 2.20971

C 4.51305 -1.37481 0.49812

H 5.27482 -2.62530 2.13717

H 2.84133 -0.27182 -0.30547

C 4.22062 -0.71311 2.72785

H 3.96497 -0.93904 3.76371

H 5.06283 -0.02108 2.70393

N -2.41394 0.23517 0.14502

H -1.40144 0.25580 0.16503

C -3.09893 1.36196 -0.12785

O -4.31285 1.45005 0.09776
C -2.31145 2.47566 -0.79102
C -2.88435 3.90295 -0.55897
C -0.80734 2.62655 -0.35384
H -2.33404 2.23655 -1.85802
C -1.63894 4.69485 -0.04608
H -3.69943 3.90907 0.16213
H -3.25432 4.33103 -1.49060
C -0.81807 2.89577 1.14204
H -0.15833 1.82949 -0.70137
C -1.31340 4.12316 1.32170
H -1.74616 5.77536 -0.10375
H -0.57926 2.16387 1.89989
H -1.56191 4.59736 2.26193
C -0.52454 4.04263 -0.89128
H -0.68211 4.13704 -1.96757
H 0.47430 4.39450 -0.63143
O 2.00662 -1.78174 -2.71617
O 0.89143 0.08905 -2.16050
H 1.69171 0.58851 -2.51885
C -3.63901 -0.53234 2.15117
O -4.64043 -1.04146 2.60426
O -2.92099 0.38445 2.81014
H -3.34534 0.56296 3.66758
S 4.48088 1.38958 0.05202
C 3.04703 2.42154 -0.45889
H 2.14038 1.90514 -0.14506
H 3.09579 3.36829 0.07978
C 3.02466 2.70880 -1.95137
H 3.88660 3.31722 -2.23642
H 2.11601 3.25710 -2.20224
O 3.00302 1.51908 -2.76384
H 3.76770 0.97574 -2.51330
H 4.92315 -1.84556 -0.38392

A_p1_R

S 3.17750 -1.93362 1.61635
S 1.52833 -2.78700 2.53910
C 0.65530 -1.36050 3.28302
H -0.13519 -1.82222 3.88122
H 1.33795 -0.85769 3.96376
C 2.98042 -2.41348 -0.13573
H 3.94322 -2.16279 -0.58228
H 2.86042 -3.49254 -0.18121
C 1.84392 -1.73069 -0.89296
H 0.91651 -1.91211 -0.34619
C 0.06577 -0.34078 2.30746
H 0.86376 0.03563 1.66844
C -0.44230 0.85238 3.11255
N -0.96541 -0.92722 1.48352
C -1.24678 -0.46139 0.25655
H -1.42728 -1.75973 1.81625
O -0.70049 0.56912 -0.17333
C -2.06836 -1.32412 -0.66714
C -2.90062 -2.51407 -0.11116
C -3.04264 -0.60942 -1.63414
H -1.28078 -1.73754 -1.30731
C -4.20162 -2.44287 -0.97506
H -3.14172 -2.41956 0.94778
H -2.37833 -3.46064 -0.25517
C -4.29460 -0.03277 -0.90667
H -2.55380 0.10418 -2.29027

C -4.98954 -1.28248 -0.46610
H -4.73689 -3.38814 -1.03020
H -4.88889 0.52761 -1.63988
C -3.66763 -1.85194 -2.30250
H -2.92651 -2.48354 -2.79515
H -4.46303 -1.60811 -3.00731
N 1.99143 -0.29685 -1.01228
H 1.28438 0.27605 -0.57309
C 2.93904 0.26971 -1.77849
O 3.73816 -0.42750 -2.42515
C 3.02965 1.77652 -1.80479
C 1.71303 2.60646 -1.88980
C 3.73712 2.39401 -0.52959
H 3.66170 2.00065 -2.66226
C 1.88125 3.65123 -0.73448
H 0.81013 2.01267 -1.76632
H 1.65054 3.10735 -2.85588
C 2.86023 2.11757 0.67751
H 4.78386 2.11307 -0.44744
C 1.76242 2.86979 0.55822
H 1.23666 4.52083 -0.83177
H 3.05149 1.35364 1.41763
H 0.88179 2.84211 1.18308
C 3.40602 3.88055 -0.77991
H 3.76473 4.24883 -1.74304
H 3.76247 4.52896 0.02106
O -1.60061 1.07227 3.37887
O 0.57423 1.61956 3.52333
H 0.22999 2.34845 4.06914
C 1.61231 -2.40544 -2.24665
O 2.00890 -3.51228 -2.53684
O 0.84743 -1.66499 -3.05708
H 0.69728 -2.15314 -3.88549
S -4.07589 1.12776 0.50155
C -3.98852 2.74978 -0.33225
H -4.80043 2.80900 -1.06143
H -4.19254 3.47894 0.45466
C -2.66819 3.09080 -1.00642
H -2.77085 4.07437 -1.47347
H -2.44247 2.37021 -1.79689
O -1.57958 3.17803 -0.08884
H -1.24228 2.27274 0.04746
H -5.77563 -1.32002 0.27428

A_p2_S

S 2.45444 -3.19762 -1.29328
S 1.37968 -1.68114 -2.16943
C -0.36373 -2.22269 -2.05730
H -0.86927 -1.72773 -2.88918
H -0.41148 -3.29710 -2.22439
C 2.63782 -2.82821 0.49113
H 1.66433 -2.65487 0.94720
H 3.02857 -3.76885 0.88238
C 3.60819 -1.69032 0.82909
H 4.49560 -1.77383 0.20126
C -1.03310 -1.85254 -0.74129
H -0.48963 -2.27130 0.10248
C -2.47577 -2.36431 -0.67409
N -1.10606 -0.41253 -0.55409
C -0.63414 0.24657 0.51529
H -1.69850 0.08397 -1.20093
O 0.08606 -0.30169 1.36379

C -1.02763 1.69456 0.70082
C -1.84096 2.45178 -0.38993
C -1.95829 1.83601 1.96854
H -0.09754 2.22257 0.91020
C -3.10202 2.94003 0.35810
H -2.08254 1.86754 -1.27700
H -1.26618 3.30720 -0.74239
C -3.18749 1.02039 1.73058
H -1.42762 1.63819 2.89603
C -4.02151 1.73996 0.71750
H -3.62898 3.74484 -0.14860
H -4.95824 2.11757 1.14257S
C -2.50677 3.26135 1.74109
H -1.72402 4.02044 1.69776
H -3.25509 3.54845 2.48050
N 2.99433 -0.40459 0.62158
H 1.99209 -0.33194 0.76241
C 3.71003 0.72179 0.43731
O 4.94588 0.70638 0.37358
C 2.87509 1.97575 0.27822
C 3.65609 3.31297 0.28138
C 2.11557 2.02114 -1.10834
H 2.12240 1.96775 1.06734
C 3.23201 3.97294 -1.07356
H 4.73215 3.16515 0.34457
H 3.34614 3.93587 1.12075
C 3.18030 1.95443 -2.18570
H 1.30762 1.30133 -1.18329
C 3.84181 3.11500 -2.16586
H 3.43284 5.04005 -1.12907
H 3.42211 1.06829 -2.75631
H 4.73383 3.36901 -2.72298
C 1.75921 3.51887 -1.15067
H 1.15889 3.84439 -0.29940
H 1.26843 3.80609 -2.08131
O -3.10591 -2.73926 -1.64407
O -2.96632 -2.29136 0.55212
H -3.97357 -2.33429 0.52875
C 4.09096 -1.84473 2.27399
O 3.76746 -1.13299 3.19660
O 4.92580 -2.88603 2.39712
H 5.19468 -2.97628 3.32822
S -4.55866 0.74462 -0.75145
C -6.17339 0.12304 -0.17318
H -6.76884 0.97307 0.16746
H -6.65995 -0.28145 -1.06407
C -6.16481 -0.92829 0.92384
H -7.19955 -1.10194 1.23607
H -5.60103 -0.59455 1.79313
O -5.57972 -2.18191 0.53252
H -5.97326 -2.47371 -0.30159
H -3.33045 -0.00605 2.03046

A_p2_R

S 0.99059 -1.38508 2.98558
S -0.57913 -2.70544 2.84118
C -0.17482 -3.82575 1.44790
H 0.82380 -4.22984 1.59520
H -0.89261 -4.63787 1.58025
C 0.35562 0.07966 2.06817
H -0.01666 -0.24516 1.10053
H -0.46438 0.51057 2.63942

C 1.44010 1.14392 1.88482
H 1.79262 1.50068 2.85271
C -0.30556 -3.28677 0.02043
H -0.27019 -4.16584 -0.62841
C 0.92329 -2.48957 -0.41607
N -1.56747 -2.62701 -0.21178
C -2.12314 -2.57368 -1.43966
H -1.93375 -2.03744 0.52132
O -1.59253 -3.13236 -2.41001
C -3.41345 -1.81562 -1.62689
C -4.41212 -1.63922 -0.44544
C -3.21033 -0.32584 -2.10989
H -3.90726 -2.33692 -2.44713
C -4.82249 -0.14877 -0.53348
H -3.99059 -1.87725 0.53106
H -5.27628 -2.28672 -0.58930
C -2.60485 0.46534 -0.99227
H -2.70356 -0.26298 -3.06975
C -3.65402 0.68637 0.03803
H -5.78726 0.06707 -0.07969
H -3.36269 0.39253 1.04743
C -4.68597 0.12976 -2.04337
H -5.34654 -0.48686 -2.65335
H -4.82155 1.17726 -2.30805
N 2.56092 0.65125 1.10685
H 2.52445 -0.28342 0.73525
C 3.55153 1.47787 0.72827
O 3.55255 2.66451 1.09053
C 4.65373 0.96779 -0.16670
C 4.90334 -0.55362 -0.36308
C 4.47971 1.47925 -1.65270
H 5.55532 1.44023 0.22333
C 4.94824 -0.70500 -1.92171
H 4.14445 -1.19938 0.07340
H 5.86045 -0.83357 0.07674
C 3.24335 0.81471 -2.23212
H 4.53385 2.56062 -1.73876
C 3.53082 -0.47925 -2.41007
H 5.43390 -1.61517 -2.26351
H 2.28985 1.30398 -2.37634
H 2.87095 -1.26387 -2.75599
C 5.58826 0.64083 -2.32191
H 6.57581 0.80230 -1.88585
H 5.63047 0.78647 -3.40159
O 2.04943 -2.92132 -0.29311
O 0.63530 -1.31720 -0.97549
H 1.45942 -0.87899 -1.26377
C 0.73438 2.32920 1.22292
O 0.32999 3.27769 1.86502
O 0.53127 2.15108 -0.07126
H -0.14134 2.80663 -0.43430
S -4.23965 2.46935 0.18497
C -2.81422 3.32380 0.95186
H -3.18979 3.87111 1.81784
H -2.11131 2.57363 1.31125
C -2.12782 4.30469 0.01880
H -1.38129 4.87180 0.57350
H -2.85184 5.00508 -0.40524
O -1.40741 3.65779 -1.04627
H -2.00697 3.03681 -1.49116
H -1.55284 0.66182 -0.85047

TS_A-A_p1_S

S -3.47138 -2.23222 -1.69259
S -2.18848 -3.74756 -1.08370
C -0.59193 -3.20664 -1.80339
H 0.06519 -4.07508 -1.71937
H -0.75187 -3.01772 -2.86357
C -4.11732 -1.48777 -0.15410
H -4.88013 -0.79809 -0.51930
H -4.61635 -2.26538 0.41834
C -3.12129 -0.75897 0.74423
H -2.35136 -1.46954 1.05828
C 0.01886 -1.98163 -1.14547
H -0.75691 -1.25788 -0.93440
C 1.05482 -1.31060 -2.05100
N 0.71834 -2.29757 0.09691
C 0.81934 -1.41983 1.11987
H 1.45247 -2.98585 0.00309
O 0.05415 -0.45702 1.23895
C 1.94811 -1.57039 2.11510
C 2.87142 -2.82012 2.12190
C 2.97111 -0.38879 1.86956
H 1.49957 -1.42060 3.09644
C 4.29825 -2.21324 1.86970
H 2.62210 -3.58168 1.38522
H 2.84511 -3.29845 3.10077
C 3.47189 -0.56766 0.44713
H 2.56470 0.58721 2.11204
C 4.25308 -1.69853 0.45875
H 5.11190 -2.88934 2.11682
H 2.92249 -0.24096 -0.41950
C 4.17959 -0.89773 2.66777
H 3.96197 -1.05900 3.72390
H 5.04924 -0.25017 2.56099
N -2.41263 0.33544 0.11529
H -1.40475 0.28197 0.20139
C -2.99531 1.50996 -0.19269
O -4.21077 1.68656 -0.03960
C -2.08862 2.56405 -0.79932
C -2.57928 4.02727 -0.60642
C -0.61306 2.61503 -0.25290
H -2.05041 2.32226 -1.86522
C -1.32608 4.73421 0.00208
H -3.44484 4.08906 0.05028
H -2.84718 4.47798 -1.56203
C -0.71759 2.88434 1.23912
H 0.00717 1.77731 -0.55478
C -1.14207 4.14208 1.38758
H -1.35643 5.81948 -0.05805
H -0.58629 2.13745 2.00869
H -1.42834 4.63157 2.30906
C -0.19830 4.00908 -0.76164
H -0.26980 4.11316 -1.84613
H 0.80018 4.29340 -0.42846
O 1.93331 -1.92624 -2.62609
O 0.93427 0.00468 -2.05692
H 1.75829 0.45755 -2.42663
C -3.80297 -0.33357 2.04723
O -4.86682 -0.75959 2.43977
O -3.05252 0.52242 2.75024
H -3.50995 0.73601 3.58220
S 4.76719 1.48847 -0.01418
C 3.28849 2.43330 -0.52881
H 2.40656 1.94815 -0.10615

H 3.35267 3.42602 -0.07807
C 3.14899 2.58589 -2.03504
H 3.97523 3.17843 -2.43588
H 2.21161 3.09173 -2.26956
O 3.09806 1.32399 -2.72881
H 3.86605 0.80186 -2.44375
H 4.62659 -2.22186 -0.41022

TS_A-A_p2_S
S 1.05889 -2.49214 -1.02238
S -0.62413 -1.37077 -1.36430
C -2.01837 -2.56661 -1.51071
H -2.45916 -2.42235 -2.49864
H -1.60963 -3.57238 -1.45579
C 1.17293 -2.77137 0.77892
H 0.35480 -3.40566 1.11055
H 2.10002 -3.34195 0.86339
C 1.28450 -1.53163 1.68424
H 1.56988 -1.91988 2.66835
C -3.09673 -2.36196 -0.43225
H -2.66712 -2.52168 0.55173
C -4.18555 -3.40983 -0.63449
N -3.65221 -1.02878 -0.49886
C -3.69725 -0.14601 0.51423
H -4.00557 -0.73245 -1.39853
O -3.32478 -0.42231 1.66506
C -4.22363 1.22674 0.13158
C -4.24804 2.27586 1.27305
C -3.31244 1.92919 -0.96238
H -5.21738 1.08935 -0.29861
C -3.38061 3.44482 0.70682
H -3.84999 1.88190 2.20554
H -5.26536 2.62084 1.45472
C -1.94653 1.96028 -0.34023
H -3.39393 1.48498 -1.95021
C -1.96160 2.90441 0.66180
H -3.51249 4.38969 1.22646
H -1.32946 2.85854 1.53539
C -3.76452 3.39012 -0.78237
H -4.83194 3.52876 -0.95655
H -3.19064 4.08344 -1.39559
N 2.27469 -0.57240 1.25629
H 1.95570 0.25267 0.75894
C 3.59854 -0.80646 1.31383
O 4.06486 -1.82441 1.84190
C 4.44362 0.26502 0.65458
C 5.96649 0.19029 0.92216
C 4.36377 0.18965 -0.92563
H 4.04529 1.23505 0.95381
C 6.57519 0.11924 -0.51880
H 6.24048 -0.67406 1.52354
H 6.31027 1.08795 1.43655
C 4.85452 -1.19003 -1.31949
H 3.40126 0.50044 -1.32135
C 6.16802 -1.23030 -1.07884
H 7.63407 0.36146 -0.56548
H 4.21320 -2.01054 -1.61007
H 6.81724 -2.09378 -1.13747
C 5.59787 1.04177 -1.27705
H 5.55287 2.05492 -0.87274
H 5.78924 1.07858 -2.35021
O -5.29061 -3.18327 -1.07093

O -3.75330 -4.62963 -0.29396
H -4.45094 -5.28323 -0.47886
C -0.03967 -0.81714 1.91549
O -0.16278 0.38998 1.91330
O -1.03244 -1.65942 2.17195
H -1.90658 -1.17860 2.11504
S -0.52704 4.72443 -0.15559
C 0.54258 3.88835 -1.37710
H -0.06452 3.21147 -1.98101
H 0.90658 4.67719 -2.04164
C 1.73965 3.14861 -0.80056
H 2.33794 3.82197 -0.17853
H 2.36864 2.79147 -1.61907
O 1.37444 1.98132 -0.06044
H 0.83229 2.23851 0.69751
H -1.15549 1.25538 -0.53287

TS_B-B_p2_R

S 0.50697 4.07324 -0.31195
S 2.51391 4.09429 -0.74952
C 2.17335 1.51699 -1.86028
H 2.28818 1.87965 -2.88393
C 0.71449 2.72977 2.23635
H 1.79469 2.58681 2.20690
C 0.09647 1.42410 1.69829
H 0.40604 1.29879 0.65981
C 2.46991 -0.00300 -1.80092
H 2.56052 -0.29010 -0.75555
C 3.76937 -0.33619 -2.49618
N 1.39889 -0.78384 -2.38910
C 0.32242 -1.15117 -1.66645
H 1.43630 -0.98698 -3.37689
O 0.23065 -0.88959 -0.46386
C -0.77957 -1.87135 -2.40625
C -1.41681 -3.03876 -1.59898
C -2.02734 -0.92758 -2.67695
H -0.41595 -2.22024 -3.37239
C -2.93486 -2.67128 -1.56652
H -0.99624 -3.12535 -0.59851
H -1.27033 -3.98648 -2.11622
C -2.46222 -0.41822 -1.33300
H -1.84239 -0.18391 -3.44679
C -3.04718 -1.46500 -0.65607
H -3.59051 -3.50367 -1.32818
H -3.08116 -1.54348 0.42031
C -3.09768 -2.00667 -2.94604
H -2.83827 -2.66535 -3.77535
H -4.08896 -1.58367 -3.10721
N 0.53680 0.26224 2.45762
H -0.20601 -0.27448 2.88261
C 1.71938 -0.35121 2.25251
O 2.64466 0.19347 1.64088
C 1.84943 -1.73260 2.86517
C 0.69995 -2.73780 2.51955
C 3.13589 -2.51390 2.43515
H 1.87989 -1.58374 3.94762
C 1.48580 -3.97862 1.98641
H 0.01053 -2.34929 1.77225
H 0.12704 -2.99572 3.41035
C 3.07646 -2.69056 0.92615
H 4.05547 -2.09762 2.83723
C 2.09564 -3.55686 0.66186

H 0.90458 -4.89775 1.97846
H 3.64935 -2.11485 0.21383
H 1.69949 -3.81838 -0.31035
C 2.73329 -3.93162 2.89393
H 2.49708 -3.99349 3.95838
H 3.47810 -4.68512 2.63575
O 3.85734 -0.79904 -3.61376
O 4.83028 -0.03221 -1.74007
H 5.64560 -0.21079 -2.24157
C -1.42633 1.41275 1.69096
O -2.09823 0.67543 2.39213
O -1.93952 2.26707 0.82734
H -2.93591 2.15402 0.73162
H 1.12181 1.61936 -1.59072
C 3.01488 2.33136 -0.88958
H 4.05510 2.40108 -1.21307
H 2.99520 1.88614 0.10169
C 0.35610 4.02709 1.51937
H 0.95846 4.84013 1.92360
H -0.69285 4.28612 1.67045
H 0.43369 2.84505 3.28682
S -5.43517 -0.85183 -0.57436
C -5.37855 -0.04065 1.06256
H -4.44800 -0.32148 1.55728
H -6.20045 -0.43626 1.66396
C -5.49859 1.47261 0.99990
H -5.37443 1.89546 1.99794
H -6.48087 1.76337 0.61760
O -4.47959 2.07522 0.18433
H -4.46097 1.58888 -0.65737
H -2.22190 0.55098 -0.92813

TS_B-B_p1_S

S -0.58977 -1.18749 1.99759
S 0.36926 -2.97811 2.32669
C 2.52057 -2.10240 0.75509
H 2.00423 -1.15150 0.61554
C -2.08327 -2.16615 -0.24952
H -3.02252 -2.67434 -0.47310
C -1.90844 -1.08957 -1.34483
H -1.95637 -1.60249 -2.30567
C 3.46074 -2.26331 -0.46347
H 2.86911 -2.43854 -1.35993
C 4.39599 -3.43870 -0.26746
N 4.25441 -1.06282 -0.65102
C 3.79791 0.02432 -1.28624
H 5.09852 -0.97917 -0.10092
O 2.72449 0.02826 -1.91541
C 4.63780 1.26884 -1.13253
C 4.43960 2.35277 -2.22331
C 4.24702 2.03355 0.19776
H 5.68551 0.98110 -1.05091
C 3.96257 3.60131 -1.40039
H 5.38487 2.57564 -2.71826
H 3.71502 2.05694 -2.97881
C 2.75680 2.30405 0.11408
H 4.59187 1.53618 1.09867
C 2.59803 3.22679 -0.89498
H 4.04266 4.54120 -1.93960
H 2.01342 1.56549 0.37108
C 4.81255 3.42681 -0.12463
H 5.88441 3.42060 -0.32437

H 4.58483 4.16017 0.64863
N -2.95083 -0.08168 -1.30221
H -2.73773 0.78221 -0.82699
C -4.24083 -0.34032 -1.59607
O -4.59310 -1.40623 -2.11396
C -5.19964 0.76400 -1.20076
C -6.66417 0.58839 -1.67012
C -5.34763 0.85832 0.37462
H -4.78775 1.71054 -1.55375
C -7.47078 0.61939 -0.32862
H -6.81617 -0.33977 -2.21712
H -6.96426 1.41701 -2.31180
C -5.82935 -0.50291 0.84019
H -4.46664 1.26053 0.86790
C -7.09030 -0.64315 0.42149
H -8.53484 0.80300 -0.45561
H -5.20151 -1.25274 1.30163
H -7.70249 -1.53334 0.47848
C -6.65556 1.66709 0.45781
H -6.59799 2.63632 -0.04133
H -7.00211 1.79751 1.48371
O 5.50827 -3.35440 0.20607
O 3.83434 -4.59190 -0.64189
H 4.43997 -5.32665 -0.43724
C -0.55329 -0.40503 -1.28777
O -0.38703 0.74476 -0.91659
O 0.41657 -1.19700 -1.70094
H 1.31020 -0.73322 -1.69288
H 3.12765 -2.02073 1.65897
C 1.49592 -3.21457 0.89783
H 1.96785 -4.17306 1.12376
H 0.91168 -3.33322 -0.01272
C -2.16892 -1.65305 1.18222
H -2.64781 -2.39810 1.81557
H -2.76980 -0.74228 1.24422
H -1.29353 -2.91030 -0.35010
S 2.15407 3.43745 2.20960
C 0.53697 2.60407 2.25974
H 0.17151 2.67356 3.28781
H 0.67069 1.54405 2.03686
C -0.48359 3.20225 1.29910
H -0.04524 3.32327 0.30633
H -0.80489 4.18402 1.64941
O -1.65725 2.37962 1.21178
H -1.41759 1.63279 0.64243
H 1.66271 3.50750 -1.3571
